# Cytotoxic Activity of Chemical Constituents of *Clerodendrum glabrum* and *Combretum nelsonii* Root Extracts Against Selected Cancer Cell Lines

**DOI:** 10.3390/plants14182832

**Published:** 2025-09-11

**Authors:** Kopelo V. Mabunda, Douglas Kemboi, Ibukun M. Famuyide, Lyndy J. McGaw, Ntebogeng S. Mokgalaka-Fleischmann, Vuyelwa Jacqueline Tembu

**Affiliations:** 1 Department of Chemistry, Tshwane University of Technology, Private Bag X680, Pretoria 0001, South Africa; kopelov@yahoo.com (K.V.M.); mokgalakans@tut.ac.za (N.S.M.-F.); 2 School of Science and Technology, University of Kabianga, P.O. Box 2030, Kericho 20200, Kenya; 3 Phytomedicine Programme, Department of Paraclinical Sciences, University of Pretoria, Private Bag X04, Onderstepoort 0110, South Africa; adeyerimi@gmail.com (I.M.F.); lyndy.mcgaw@up.ac.za (L.J.M.)

**Keywords:** *Clerodendrum glabrum*, *Combretum nelsonii*, cytotoxicity, Caco-2, MCF-7

## Abstract

Breast and colon cancers are leading causes of death worldwide. There is a need for improved treatment strategies. South African medicinal plants, including *Clerodendrum glabrum* (*C. glabrum*) and *Combretum nelsonii* (*C. nelsonii*), are known for their cytotoxic properties. This study aimed to isolate and characterize terpenoids and stilbenes from the roots of *C. glabrum* and *C. nelsonii* and evaluate their anticancer potential against colorectal adenocarcinoma (Caco-2) and hormone receptor-positive breast cancer (MCF-7) cell lines. Spectroscopic techniques including nuclear magnetic resonance spectroscopy (NMR) were used to characterize the isolated compounds. Repeated column chromatography of *C. glabrum* extract led to the isolation of ferruginol (**1**), royleanone (**2**), and *β*-amyrin palmitate (**3**). *C. nelsonii* extract afforded combretastatin A-1 (**4**), a mixture of combretastatin A-1-2′-*O*-*β*-D-glucopyranoside (**5a**) and combretastatin B-1-2′-*O*-*β*-D-glucopyranoside (**5b**). Compounds **1**, **2**, **4**, **5a,** and **5b** were isolated for the first time from the plant species. *C. glabrum* extract showed good anticancer properties with LC_50_ of 1.30 × 10^3^ µg/mL (CaCo-2) and 2790 µg/mL (MCF-7). Compound (**1**) exhibited high toxicity against the Caco-2 at LC_50_ of 24.3 µg/mL and moderate activity against MCF-7 at 48.4 µg/mL. Compound (**4**) and the mixture (**5a** and **5b**) showed moderate activity against the MCF-7 at LC_50_ 72.0 and 44.1 µg/mL, respectively. These findings highlight *C. glabrum* and *C. nelsonii* as promising sources of anticancer lead compounds.

## 1. Introduction

In 2019, the World Health Organization (WHO) reported that cancer was the second leading cause of death in more than 100 countries, including South Africa [1]. Furthermore, breast and colorectal cancers were among the most frequently diagnosed, with approximately 2.3 million and 1.2 million new cases, respectively, [2,3]. The incidence of breast cancer remains high in many African countries [4]. In South Africa, breast cancer is predominantly diagnosed in women, and in 2022, the Global Cancer Observatory reported that breast cancer accounted for 14.3% of all new cancer cases in both males and females [5].

Colorectal cancer is also a major global health concern, with incidence expected to increase by more than 60% by 2030, reaching an estimated 2.2 million new cases and 1.1 million deaths annually [6]. In South Africa, colorectal cancer is ranked among the ten most common cancers in both men and women [7,8]. A study by Motsuku and co-authors further reported that colorectal cancer is more frequently diagnosed in Black South Africans residing in the North West, Mpumalanga, and Limpopo provinces compared to other regions [9]. Similarly, breast cancer is the most prevalent malignancy among Black South African women, and its high burden has been associated with late-stage diagnosis, limited access to healthcare, socioeconomic disparities, and possible genetic or lifestyle factors [9].

Current treatment options for both breast and colorectal cancers include chemotherapy, radiotherapy, and combined therapeutic approaches [10,11,12,13].

Despite the availability of various cancer treatments options, it is essential to develop new strategies that can target all types of cancer. Current chemotherapeutic agents have devastating side effects and are cytotoxic to normal cells [14,15,16]. Medicinal plants have been proven to produce compounds that could be lead compounds for anticancer drugs that are affordable, safe, and non-toxic. Moreover, crude extracts and some chemical constituents from South African medicinal plants are reported to have cytotoxic properties [17].

**Figure 1:**
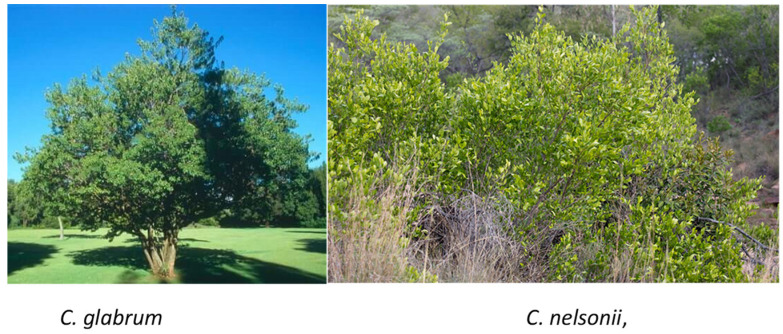
Picture of *C. glabrum* tree and *C. nelsonii* shrub.

The genus *Clerodendrum* (Lamiaceae) has approximately 584 species across Africa, Australia, Asia, South and Central America [18,19]. *C. glabrum* tree (Figure 1), also known as Tinderwood, is a tree with many branches and smooth, ovate leaves. In southern Africa, it is widely distributed along forest margins in regions of Limpopo, Kwa Zulu Natal, Eastern Cape, Mpumalanga, Swaziland, and Zimbabwe. Only a variety of *glabrum* is found in southern Africa [20]. *C. glabrum* leaves are traditionally prepared as decoctions to treat cough, diarrhea, colic, and fever, while root infusions are used to treat snakebites, arthritis, and worms in donkeys. Infused leaves are also reported to be effective against malaria [21,22]. Phytochemical investigation of different *Clerodendrum* species has been reported. For instance, Jadeja and co-authors reported the presence of polyphenols, steroids saponins and flavonoids from the leaves extracts of *C. glabrum* leaves [23]. Furthermore, five compounds isolated from *C. glabrum* leaves were n-butyl-*β*-D-fructofuranoside, n-butyl-α-D-fructofuranoside, 2′-*O*-(*β*-D-apiofuranosyl)-mussaenosidic acid, n-butyl-α-D-fructopyranoside, and ferulic acid [24]. Triterpenoids such as 3*β*-olean-12-en-3-yl palmitate, 3*β*-hydroxy-5-glutinene, 3*β*-lup-20(29)-en-3-palmitate, 3*β*-lup-20(29)-en-3-ol, and stigmasta-5,22-dien-3*β*-ol were isolated from the stem bark of *C. glabrum* [25,26]. Extracts from *Clerodendrum* were also reported to possess different biological activities including antimalaria [27], anti-inflammation [28], antimicrobial [29] antioxidant [30], and anticancer [31] activities.

*Combretum* is the most well-known genus of the Combretaceae family and contains approximately 370 species. Most of these species are found in Asia, America, India, Madagascar, Malesia, and Australia. Traditionally, the roots of *C. nelsonii* (Figure 1) are used as analgesics, diuretics, antiseptics, and to treat eye infections and wounds [32,33,34]. *Combretum* species includes trees and shrubs [35]. This tree is found in Limpopo, Mpumalanga and Northwest provinces in South Africa [36]. *C. nelsonii* was investigated against fungal pathogens and showed activity in the range of 0.02–0.08 mg/mL [37]. Two related triterpenoids from *C. nelsonii* leaves, asiatic and arjunolic acid were reported to have antifungal properties [38].

*C. glabrum* and *C. nelsonii*, were selected due to preliminary evidence of bioactivity and underexplored anticancer potential. The phytochemistry and evaluation of isolated chemical constituents from both plant species have not been extensively studied. Although both species have been investigated for their antimicrobial and anti-inflammatory properties, reports on their anticancer potential are insufficient. The focus of the study was to investigate the phytochemistry and anticancer activities of *C. glabrum* and *C. nelsonii* against Caco-2 and MCF-7 cancer cells. The anticancer studies of both plant species against heterogeneous human epithelial colorectal adenocarcinoma (Caco-2) and hormone receptor positive breast cancer (MCF-7) cell lines were investigated for the first time.

## 2. Results and Discussion

### 2.1. Compounds Isolated from C. glabrum and C. nelsonii

**Figure 2:**
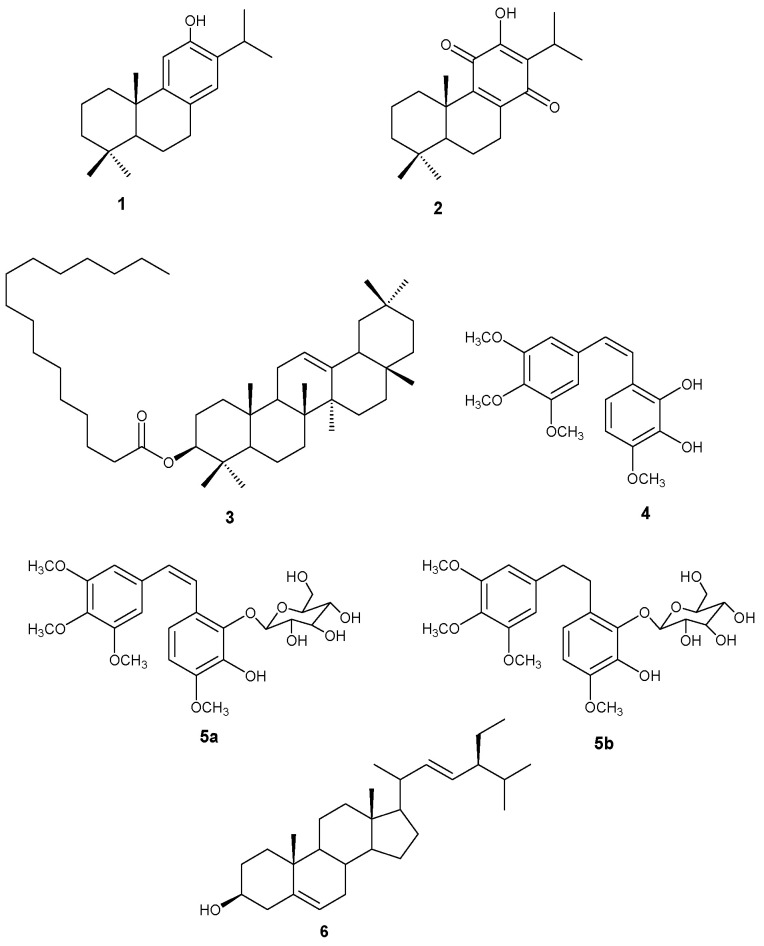
Isolated compounds from the roots of *C. glabrum* and *C. nelsonii*.

In this study, a sequential extraction method was used to extract compounds from *C. glabrum* and *C. nelsonii*. Repeated column chromatography of extracts led to the isolation of six compounds namely 12-hydroxy-abieta-8,11,13-triene (ferruginol **1**), 12-hydroxy-8,12-abietadiene-11,14-dione (royleanone **2**) and 3β-olean-12-en-3yl-palmitate (*β*-amyrin palmitate **3**), 3,4,5-trimethoxy-2′,3′hydroxyl-4′methoxy-stilbene (combretastatin A-1 **4**), a mixture of two stilbene derivatives, combretastatin A-1-2′-O-*β*-D-glucopyranoside **5a** and combretastatin B-1-2′-O-*β*-D-glucopyranoside (**5b**) and stigmasta-5,22-dien-3β-ol (**6**) as shown in Figure 2, which were further evaluated for MCF-7, Caco-2 and Vero cancer cells.

### 2.2. Structure Elucidation of Compounds Isolated from C. glabrum and C. nelsonii

12-hydroxy-abieta-8,11,13-triene (ferruginol) (**1**) showed the characteristic signals of an isopropyl group appearing at δ 3.08 (1H, m), δ 1.23 (3H, d, *J* = 4.0 Hz), and 1.22 ppm (3H, d, *J* = 4.0 Hz), which is atypical of an abietane diterpenoid. Three methyl singlets appearing at δ 0.92 (3H, s), δ 0.90 (3H, s), and δ 1.16 ppm (3H, s) were observed in the ^1^H NMR spectrum (Figure S1). Conjugated carbons were seen on the carbon resonances δ 127.2, δ 110.9, and δ 131.4 ppm ascribed to C-8, C-11, and C-13 in the ^13^C NMR spectrum (Figure S2). At the assigned C-12, an exchange proton peak of an (O-H) group was observed on the ^1^H NMR spectrum appearing at δ 4.52 ppm as a singlet, and this was supported by the sharp peak on the IR spectrum (Figure S6) appearing at 3333.3 cm^−1^ as a primary (O-H) stretch. The ^1^H NMR and ^13^C NMR spectral data were compared to that of the literature [39] to confirm the structure of the isolated compound. Ferruginol was isolated for the first time [40], from the roots of *Salvia miltorrhiza*, and was also isolated from *C. eriophyllum* [41]. Furthermore, compound (**1**) was isolated for the first time from *C. glabrum* roots.

12-hydroxy-8,12-abietadiene-11,14-dione (**2**) known as royleanone was obtained as the derivative of Compound (**1**). The IR spectra (Figure S13) exhibited differences between Compound (**1**) and (**2**), through a conjugated absorption of the para-quinone moiety appearing at 1648.5, 1629.6 and 1603.1 cm^−1^. A sharp peak appearing in the IR spectrum displayed a primary (O-H) at 3337.0 cm^−1^. A singlet proton resonance downfield at δ 7.20 ppm was hydroxyl group proton ascribed to H-12 in the ^1^H NMR spectrum (Figure S8). Compound (**2**) was isolated for the first time from *C. glabrum* roots. The spectral data obtained was compared to that of literature [41], and previously isolated from the roots of *Inula royleana* [42], and *Salvia mellifera* [43].

Compound (**3**), an oleanane type pentacyclic triterpenoid, 3*β*-olean-12-en-3-yl-palmitate commonly known as *β*-amyrin palmitate was obtained as a white powder. Eight methyl singlet resonances were observed on the ^1^H NMR spectrum (Figure S15) at δ 0.85, δ 0.85, δ 0.82, δ 0.96, δ 1.22, δ 0.82, δ 0.85 and δ 0.81 ppm assigned to 3H-23, 3H-24, 3H-25, 3H-26, 3H-27, 3H-28, 3H-29 and 3H-30, respectively. The carbon resonance δ 173.7 ppm ascribed as C-1′ is supported by the carbonyl stretch present in IR spectrum (Figure S20) at 1732.4 cm^−1^ (C=O) and 1063.0 cm^−1^ (C-O) which is a typical ester group. The carbon resonances at δ 22.7–δ 34.8 ppm ascribed to C-2′ to C-15′ indicated methylene palmitate chain. The ^13^C NMR spectrum (Figure S16) where the double bond was seen at the carbon resonances δ 121.6 and δ 145.1 ppm were ascribed to C-12 and C-13, respectively. The spectral data obtained of *β*-amyrin palmitate was compared to that of literature [25], and previously isolated from different species [44,45].

Compound (**4**), a stilbene namely 3,4,5-trimethoxy-2′,3′ hydroxyl-4′methoxy-stilbene known as combretastatin A-1 showed two aromatic rings connected by an ethylene moiety were observed with the proton doublet resonances appearing downfield at δ 6.39 (1H, d, *J* = 3.6 Hz) and δ 6.52 ppm (1H, d, *J* = 3.6 Hz) ascribed to H-1a and H-1′a. The ^13^C NMR spectrum (Figure S23) displays 18 carbon resonances with four methoxy groups appearing at the carbon resonances δ 55.9, δ 60.9, δ 55.9, δ 56.2 ppm ascribed to 3-OCH_3_, 4-OCH_3_, 5-OCH_3_, and 4′-OCH_3_, respectively. Moreover, ^1^H NMR spectrum (Figure S22) showed the proton resonance at δ 5.43 ppm appearing as a broad singlet of an (O-H) assigned to H-2′ and H-3′. The interpreted data obtained confirmed that the compound belong to the stilbene class and was compared to the literature data. First isolation of combretastatin A-1 was from *C. caffrum* [46] and later isolated from *C. kraussii* [47].

Compound (**5**) was obtained as a mixture of stilbenes, namely combretastatin A-1-2′-O-*β*-D-glucopyranoside (**5a**) and combretastatin B-1-2′-O-*β*-D-glucopyranoside (**5b**). The mixture showed the characteristics of a stilbene type with two aromatic rings connected with an ethylene moiety. The ^1^H NMR spectrum (Figure S29) of compound (**5a**) displayed the ethylene moiety where the proton resonances appearing at δ 6.30 (1H, d, *J* = 5.2 Hz) and δ 6.36 ppm (1H, d, *J* = 5.6 Hz) were observed. Compound (**5b**) showed two methylene proton resonances appearing at δ 2.69 (2H, m) and δ 2.71 ppm (2H, m). The ^13^C NMR spectrum (Figure S30) further showed a double bond appearing at the carbon resonances δ 127.9 and δ 125.3 ppm assigned to C-1a and C-1′a in Compound (**5a**). The difference was seen in Compound (**5b**) where there was no double bond in the assigned C-1a and C-1′a with the methylene carbon resonances at δ 37.0 and δ 31.0 ppm. The carbon resonance at δ 105.2 ppm ascribed to C-1″ indicated the sugar moiety on both Compounds. Both aromatic rings showed similarities where four methoxy groups appearing at the carbon resonances δ 55.8, δ 56.0, δ 60.4, and δ 60.8 ppm ascribed to 3-OCH_3_, 4-OCH_3_, 5-OCH_3_, and 4′-OCH_3_, were observed. In addition, a broad singlet with a proton resonance at δ 5.49 (OH, bs) was observed in the ^1^H NMR spectrum (Figure S29). A mixture of both compounds was isolated from *Combretum kraussii* [48]. The spectral data of a mixture obtained was compared to that of literature [47], to confirm the structure.

Compound (**6**), a common phytosterol known as stigmasterol, was isolated from both *C. glabrum* and *C. nelsonii*. The ^13^C NMR spectrum (Figure S35) showed 29 carbon resonances, six methyl resonances and three quaternary carbons. The alkene double bonds were observed at the carbon resonances at δ 140.7, δ 121.6, δ 138.3, and δ 129.3 ppm assigned to C-5, C-6, C-22 and C-23, respectively. The ^13^C NMR spectrum (Figure S35) showed the presence of a (C-O) appearing at the carbon resonance δ 71.7 ppm with the (O-H) proton resonance at δ 3.22 ppm (1H, m) assigned to H-3. The spectral data obtained was compared to that of the literature [49]. Stigmasterol was isolated previously from many plant species including *C. fragnans* [50].

### 2.3. Spectral Data of Isolated Compounds

#### 2.3.1. Ferruginol (**1**)

Yellow powder, (38.9 mg) 0.0024% *w*/*w*, melting point: 173–176 °C, ${\left[\alpha \right]}_{23}^{D}$ = +39.42°, c = 0.25 in CHCl_3_, mass spectrum: [M+H]^+^ at *m*/*z* 287.0488, (calculated for C_20_H_30_O, M^+^ at *m*/*z* 286.4625), FT-IR: 2921.5, 2853.5 cm^−1^ (C-H stretch), 1648.5, 1629.6, 1603.1 cm^−1^ (C=C aromatic benzene) 3333.3 cm^−1^ (phenolic absorption O-H), ^1^H NMR (400 MHz, CDCl_3_): δ 0.90 (3H, s, H-19), 0.92 (3H, s, H-18), 1.16 (3H, s, H-20), 1.20 (1H, m, H-3), 1.22 (3H, d, *J* = 1.22, 4.0 Hz), 1.23 (3H, d, *J* = 1.24, 4.0 Hz), 1.32 (1H, t, H-5), 1.37 (1H, m, H-1), 1.47 (1H, m, H-3), 1.62 (1H, m, H-2), 1.70 (1H, m, H-6), 1.82 (1H, m, H-2), 1.87 (1H, m, H-6), 2.16 (1H, m, H-1), 2.83 (2H, m, H-7), 3.08 (1H, m, H-15), 4.52 (1H, s, H-12), 6.61 (1H, s, H-11), 6.81 (1H, s, H-14); ^13^C NMR (100.6 MHz, CDCl_3_): δ 19.2 (C-2), 19.3 (C-6), 21.6 (C-19), 22.5 (C-16), 22.7 (C-17), 24.7, (C-20), 26.8 (C-15), 29.7 (C-7), 33.3 (C-4, C-17), 36.2 (C-1), 37.5 (C-10), 41.7 (C-3), 50.3 (C-5), 110.9 (C-11), 126.6 (C-14), 127.2 (C-8), 131.4 (C-13), 148.6 (C-9), 150.7 (C-12) as in Table S1.

#### 2.3.2. Royleanone (**2**)

Yellow powder, (20.8 mg) 0.0013% *w*/*w*, melting point: 178.4–180.8 °C, ${\left[\alpha \right]}_{24}^{D}$ = +17.3°, c = 0.24 in CHCl_3_, mass spectrum: [M+Na]^+^ at *m*/*z* 339.9256, (calculated for C_20_H_28_O_3_Na, M^+^ at *m*/*z* 339.4351), FTIR: 3337.0 cm^−1^ (O-H band), 1648.5, 1629.3, 1603.1 cm^−1^ (C=C para-quinone moiety), 2925.3, 2849.7 cm^−1^ (C-H stretch), ^1^H NMR (400 MHz, CDCl_3_): δ 0.91 (1H, s, H-18), 0.93 (3H, s, H-19), 1.06 (1H, t, H-5, *J* = 21.2 Hz), 1.11 (1H, m, H-1), 1.19 (1H, m, H-3), 1.21 (2H, m), H-2, 1.22 (3H, d, *J* = 22.7 Hz), 1.24 (3H, d, *J* = 22.5, 8.0 Hz), 1.41 (1H, ddd, H-6, *J* = 5.2, 4.4, 4.5 Hz), 1.46 (1H, m, H-3), 1.87 (1H, q, H-6, *J* = 20.8 Hz, H-20), 2.37 (H, dd, H-7, *J* = 0.8, 1.6 Hz), 2.71 (1H, dd, H-7, *J* = 1.2, 0.8 Hz), 2.75 (1H, m, H-1), 3.15 (1H, sept, H-15, *J* = 28.4 Hz), 7.20 (1H, s, H-20), ^13^C NMR (100.6 MHz, CDCl_3_): δ 17.4 (C-6), 19.2 (C-2), 19.8 (C-17), 19.9 (C-16), 20.0 (C-20), 21.7 (C-18), 24.1 (C-15), 26.6 (C-7), 33.4 (C-4, C-19), 36.2 (C-1), 38.4 (C-10), 41.7 (C-3), 51.7 (C-5), 123.7 (C-13), 146.0 (C-9), 146.5 (C-8), 183.4 (C-11), 150.5 (C-12), 187.5 (C-14) as in Table S1.

#### 2.3.3. *β*-Amyrin-Palmitate (**3**)

White powder, (21.4 mg) 0.0013% *w*/*w*, melting point: 70.0–73.0 °C, ${\left[\alpha \right]}_{24}^{D}$ = +58.1°, c = 0.40 in CHCl_3_, mass spectrum: [M+H]^+^ at *m*/*z* 666.7625, (calculated for C_46_H_80_O_2_, M^+^ at *m*/*z* 665.1368), FT-IR: 1063.0 cm^−1^ (C-O stretch), 1474.7, 1463.4 cm^−1^ (C=C stretch), 2917.7, 2849.7 cm^−1^ (C-H stretch), ^1^H NMR (400 MHz, CDCl_3_): δ 0.81 (3H, s, H-30), 0.82 (3H, s, H-25, H-28), 0.85 (3H, s, H-23, H-24, H-29) 0.86 (1H, t, H-5, *J* = 10.8 Hz), 0.96 (3H, s, H-26), 1.00 (1H, m, H-19), 1.12 (1H, m, H-16), 1.18–1.27 (10H, m, H-4′-13′), 1.21 (1H, m, H-7), 1.22 (3H, s, H-27), 1.23 (1H, m, H-22), 1.24 (2H, m, H-15), 1.26 (1H, m, H-19), 1.27 (1H, m, H-1, H-14′-15′), 1.42 (1H, m, H-6), 1.54 (1H, t, *J* = 10.8 Hz), 1.62 (1H, m, H-1), 1.60 (1H, m, H-2), 1.85 (1H, m, H-2), 1.86 (1H, m, H-11), 1.92 (1H, m, H-3′), 1.94 (1H, t, *J* = 1.3 Hz, H-18), 2.26 (1H, m, H-21), 2.29 (1H, m, H-2′), 4.49 (1H, t, *J* = 4.0 Hz, H-3), 5.17 (1H, s, H-12), ^13^C NMR (100.6 MHz, CDCl_3_): δ 14.1 (C-16′), 15.5 (C-26), 16.7 (C-24), 18.2 (C-6), 19.7 (C-25), 22.7 (C-15′), 23.5 (C-11), 23.6 (C-2), 23.7 (C-30), 25.2 (C-3′), 25.9 (C-27), 26.9 (C-15), 26.1 (C-16), 28.0 (C-28), 28.4 (C-23), 31.1 (C-20), 31.9 (C-14′), 32.5 (C-17), 32.6 (C-7), 33.3 (C-29), 34.7 (C-21), 34.8 (C-2′), 29.1–29.7 (C-4′-13′), 36.8 (C-10), 37.1 (C-22), 37.7 (C-4), 38.2 (C-1), 39.8 (C-8), 41.7 (C-14), 46.8 (C-18), 47.2 (C-19), 47.5 (C-9), 55.2 (C-5), 80.6 (C-3), 121.6 (C-12), 145.2 (C-13), 173.3 (C-1′) as in Table S2.

#### 2.3.4. Combretastatin A-1 (**4**)

White powder, (50.0 mg) 0.0036% *w*/*w*, melting point: 108.0–110.0 °C, ${\left[\alpha \right]}_{24}^{D}$ = +12.8°, c = 0.78 in CHCl_3_, mass spectrum: [M+H]^+^ at *m*/*z* 332.9287, (calculated for C_18_H_18_O_6_ M^+^ at *m*/*z* 330.3366), FT-IR: 3329.5 cm^−1^ (broad O-H), 1595.6, 1505.0 cm^−1^ (C=C stretch of a benzene ring), 1240.5, 1092.0 cm^−1^ (C-O stretch of a methoxy), ^1^H NMR (400 MHz, CDCl_3_): δ 3.66 (3H, s, 3-OCH_3_,5-OCH_3_), 3.70 (3H, s, 4-OCH_3_, 4′-OCH_3_), 3.66 (3H, s, 3-OCH_3_), 4.44 (1H, d, *J* = 6.8 Hz, H-1″), 5.49 (1H, bs, H-2′,H-3′), 6.36 (1H, d, *J* = 8.8 Hz, H-5′), 6.39 (1H, d, *J* = 3.6 Hz, H-1a), 6.52 (1H, d, *J* = 3.6 Hz, H-1′a), 6.75 (1H, d, *J* = 2.8 Hz, H-6′), ^13^C NMR (100.6 MHz, CDCl_3_): δ 55.9 (3,5-OCH_3_), 56.2 (4′-OCH_3_),60.9 (4-OCH_3_), 61.0 (C-6″), 69.2 (C-4″), 73.8 (C-2″), 76.2 (C-3″), 76.7 (C-5″), 102.9 (C-5′), 105.0 (C-1″), 105.5 (C-6), 106.2 (C-2), 117.9 (C-1′), 120.3 (C-6′), 125.3 (C-1′a), 130.3 (C-1a), 132.5 (C-1), 132.6 (C-4), 137.3 (C-3′), 141.6 (C-2′), 146.3 (C-4′), 152.8 (C-3), 153.0 (C-5) as in Table S3.

#### 2.3.5. Combretastatin A-1-2′-*O*-*β*-D-Glucopyranoside (**5a**) and Combretastatin B-1-2′-*O*-*β*-D-Glucopyranoside (**5b**)

Reddish-brown powder, (45.5 mg) 0.0033% *w*/*w*, melting point: 71–73 °C, ${\left[\alpha \right]}_{24}^{D}$ = +27.2°, c = 1.4 in CHCl_3_, mass spectrum: [M+H]^+^ 495.9207 at *m*/*z*, (calculated for C_24_H_30_O_11_, M^+^ at *m*/*z* 494.5013) and [M+H]^+^ at *m*/*z* 497.9348, (calculated for C_24_H_32_O_11_, M+ at *m*/*z* 496.5172), FT-IR: 3350.0 cm^−1^ (broad O-H), 1591.8 cm^−1^ (C=C aromatic ring stretch), 1240, 1078, 1010 cm^−1^ (C-O methoxy), 2932.8 and 2853.5 cm^−1^ (C-H stretch), ^1^H NMR (400 MHz, CDCl_3_): (5a): δ 3.56 (3H, s, 4′-OCH_3_), 3.69 (3H, s, 4-OCH_3_), 3.71 (6H, s, 3-OCH_3_, 5-OCH_3_), 6.30 (1H, d, *J* = 3.6 Hz, H-1a), 6.36 (1H, d, *J* = 5.6 Hz, H-1a’), (5b): δ 2.70 (2H, m, H-1a), 2.71 (2H, d, *J* = 6.4 Hz, H-1′a), 3.56 (3H, s, 4′-OCH_3),_ 3.70 (3H, s, 5-OCH_3_), 3.73 (6H, s, 3-OCH_3_, 5-OCH_3_), ^13^C NMR (100.6 MHz, CDCl_3_): (5a): δ 55.8 (3,5-OCH_3_), 56.0 (4′-OCH_3_), 60.4 (4-OCH_3_), 61.0 (C-6″), 69.2 (C-4″), 73.8 (C-2″), 76.2 (C-3″), 76.7 (C-5″), 105.0 (C-1″), 105.7 (C-6), 106.2 (C-2), 108.6 (C-5′), 119.5 (C-6′), 124.1 (C-1′), 125.3 (C-1a’), 127.9 (C-1a), 132.5 (C-1), 135.8 (C-4), 138.9 (C-3′), 143.5 (C-2′), 147.8 (C-4′), 152.7 (C-3), 152.8 (C-5), (5b): δ: 31.0 (C-1a’), 37.0 (C-1a), 55.8 (3,5-OCH_3_), 56.0 (4′-OCH_3_), 60.4 (4-OCH_3_), 61.0 (C-6″), 69.2 (C-4″), 73.8 (C-2″), 76.1 (C-3″), 76.7 (C-5″), 105.0 (C-1″), 105.5 (C-2), 105.7 (C-6), 108.2 (C-5′), 119.5 (C-6′), 129.9 (C-1′), 135.8 (C-4), 138.1 (C-1), 138.8 (C-3′), 146.7 (C-4′), 143.5 (C-2′), 152.7 (C-3), 152.8 (C-5) as in Table S3.

#### 2.3.6. Stigmasterol (**6**)

White crystals (66.5 mg) 0.0022% *w*/*w*, melting point: 171.4–175.4 °C, ${\left[\alpha \right]}_{24}^{D}$ = +33.7°, c = 0.32 in CHCl_3_, mass spectrum: [M+H]^+^ at *m*/*z* 413.2500, (calculated for C_29_H_48_O, M^+^ at *m*/*z* 412.6976), FT-IR: 3456.0 cm^−1^ (O-H stretch), 2923.7, 2851.0 cm^−1^ (C-H stretch), 1457.3 cm^−1^ (C=C stretch), ^1^H NMR (400 MHz, CDCl_3_): δ 0.68 (3H, s, H-18), 0.74 (3H, t, *J* = 15.6 Hz, H-29), 0.78 (3H, d, *J* = 8.8 Hz, H-27), 0.81 (3H, d, *J* = 6.0 Hz, H-26), 0.92 (1H, dt, *J* = 6.8, 5.8 Hz, H-9), 0.95 (3H, s, H-19), 0.98 (3H, s, H-21), 1.01 (1H, m, H-14), 1.03 (1H, m, H-15), 1.04 (1H, dd, *J* = 7.6, 4.0 Hz, H-1), 1.10 (1H, m, H-17), 1.17 (1H, m, H-12), 1.18 (1H, m, H-25), 1.21 (IH, m, H-16, H-28), 1.37 (1H, m, H-28), 1.46 (2H, m, H-11), 1.48 (1H, m, H-24), 1.51 (H, m, H-15), 1.67 (1H, m, H-16), 1.80 (2H, dd, *J* = 4.0, 3.2 Hz, H-7), 1.85 (1H, dd, *J* = 4.0, 3.2, H-1), 1.90 (1H, m, H-2), 1.94 (1H, m, H-2), 1.98 (1H, m, H-12), 2.15 (1H, m, H-8), 2.00 (1H, dd, *J* = 4.8, 8.8 Hz, H-20), 2.20 (1H, m, H-4), 3.22 (1H, m, H-3), 4.97 (1H, d, *J* = 4.0 Hz, H-23), 5.05 (1H, d, *J* = 8.8 Hz, H-22), 5.32 (1H, d, *J* = 3.2 Hz, H-6), ^13^C NMR (100.6 MHz, CDCl_3_): δ 15.5 (C-26), 16.7 (C-24), 18.2 (C-6), 19.7 (C-25), 23.5 (C-11), 23.6 (C-2), 23.7 (C-30), 25.9 (C-27), 26.1 (C-16), 26.9 (C-15), 28.0 (C-28), 28.4 (C-23), 31.1 (C-20), 32.5 (C-17), 32.6 (C-7), 33.3 (C-29), 34.7 (C-21), 37.1 (C-22), 37.2 (C-1), 37.7 (C-4), 37.8 (C-10), 39.8 (C-8), 41.7 (C-14), 46.8 (C-18), 47.2 (C-19), 47.5 (C-9), 55.2 (C-5), 80.6 (C-3), 121.6 (C-12), 145.2 (C-13) as in Table S2.

### 2.4. Selective Cytotoxic Activity of Extracts and Isolated Compounds

The antiproliferative activity of the crude extracts and compounds isolated from *C. glabrum* and *C. nelsonii* were tested against heterogenous human epithelial colorectal adenocarcinoma (Caco-2) and hormone receptor positive breast cancer (MCF-7) cells using the MTT assay. The Vero African green monkey kidney cells were used as the non-cancerous cell line for comparison. Doxorubicin was used as the positive control.

Ferruginol (**1**) was highly toxic to the normal cell line with low LC_50_ value of 0.0002 µg/mL as indicated in Table 1. Moreover, ferruginol had more distinctive cytotoxicity towards Caco-2 with LC_50_ 24.3 µg/mL and moderately cytotoxic towards MCF-7 with LC_50_ value of 48.4 µg/mL. Royleanone (**2**) showed no toxicity to Caco-2 and MCF-7 cells at the maximum tested concentration (200 µg/mL) as the LC_50_ was greater than 200 µg/mL. Ferruginol showed low SI for Caco-2 (8.23 × 10^−6^) and MCF-7 (4.13 × 10^−6^) cancer cell lines. The selectivity index of royleanone was <0.15 on both MCF-7 and Caco-2 cancer cell lines. Combretastatin A-1 (**4**) isolated from *C. nelsonii* was highly toxic to the Vero cells with LC_50_ value of 15.3 µg/mL and moderately toxic on the MCF-7 cancer cell line with LC_50_ of 72.0 µg/mL. A mixture of stilbene derivatives, combretastatin A-1-2′-*O*-*β*-D-glucopyranoside (**5a**) and combretastatin B-1-2′-*O*-*β*-D-glucopyranoside (**5b**) had good selectivity index at 1.90 which can further be investigated as a promising anticancer lead drug. The higher selectivity index is an indication that the extract is more toxic to cancer cells than to normal cells, thus suggesting potential as a selective anticancer agent.

The mixture of stilbene derivatives (combretastatin A-1-2′-*O*-*β*-D-glucopyranoside and combretastatin B-1-2′-*O*-*β*-D-glucopyranoside) had an LC_50_ at 44.1 µg/mL and 83.8 µg/mL against the MCF-7 and Vero cells, respectively, showing moderate toxicity to the cells. The stilbene mixture was selectively toxic to the cancer cell with a selectively index of 1.90. This indicates that a mixture of two compounds can be used to further explore the MCF-7 breast cancer therapies. Stigmasterol (**6**) was not toxic to Caco-2, MCF-7, and Vero cells were at the highest tested concentration of 200 µg/mL and 137.0 µg/mL, respectively, and hence the low SI.

The DCM extracts of *C. glabrum* showed no activity against MCF-7 and Caco-2 with LC_50_ values of 2790 and 1300 µg/mL and selectivity indices (SI) of 1.54 and 3.31, respectively. The EtOAc extract of *C. nelsonii* had low cytotoxicity against MCF-7 cells, with LC_50_ of 252 µg/mL and a low selectivity index of 0.46. To date, no anticancer studies have investigated the cytotoxic effects of DCM extracts from *C. glabrum* against MCF-7 cells. However, other studies showed that the DCM extracts from *C. villosum* and *C. indicum* were active against the human colon carcinoma SW620 cell lines with IC_50_ values > 200 µg/mL [40]. In comparison to the literature on the cytotoxicity studies in MCF-7 cancer cell lines, the EtOAc extract from the leaves of *C. rupicola* exhibited moderate activity against MCF-7 with IC_50_ of 65.9 µg/mL [41]. The hexane and methanol extracts were not investigated due to low extract yield. The TLC profiling for hexane also showed minimum secondary metabolites.

### 2.5. Previous Studies and Mechanism of Action

Various biological activities have been investigated previously for all the isolated compounds including anticancer studies. Table 2 below compares the cytotoxicity of extracts and isolated compounds from *C. glabrum* and *C. nelsonii* with the literature reports. For instance, ferruginol was established to be a potential therapeutic agent for breast cancer. Ferruginol was reported to have anticancer properties against human gastric fibroblast AGS cells, human liver hepatocellular carcinoma HepG2 cells and colon cancer cells [42,43,44]. Further findings by Ho et al. [43] gave insights into the molecular mechanisms of ferruginol-induced apoptosis in non-small cell lung cancer (NSCLC) cells, which revealed that ferruginol could be a potential candidate drug for anti-NSCLC.

Royleanone inhibited the growth of prostate cancer cells (LNCaP) with IC_50_ of 12.5 µM [45]. Moreover, royleanone was reported to have anticancer activities against the gastric adenocarcinoma AGS cells with IC_50_ of 18 µM [46], breast MDA-MB-231 cancer cell lines with IC_50_ > 250 µM, and human colon HCT116 [47]. Combretastatins have been extensively investigated for their antitumor properties [48] by inhibiting tubulin polymerization agents with selective activity against the tumor vasculature [49]. In addition, combretastatin A-1 was reported as a potent inhibitor of tubulin with IC_50_ of 1.1 µM. More anticancer studies on combretastatin A-1 showed high cytotoxicity to human cancer cell lines such as pancreas-a (BXPC-3) with (GI_50_) of 4.4 µg/mL, prostate (DU-145) with GI_50_ of 0.017 µg/mL, leukemia (P388) with GI_50_ of 0.3 µg/mL, colon (KM20L2) with GI_50_ of 0.061 µg/mL, and lung-NCS (NCI-H460) with GI_50_ of 0.74 µg/mL [50]. Anticancer studies of stigmasterol were also evaluated and reports showed that this compound exhibited anticancer properties against colon cancer cell SW620 with IC_50_ of 2.79 µmol/L, and colon cancer cell Caco-2 with IC_50_ of 132.5 ± 33.3 µM. Stigmasterol showed no activity against MCF-7 and triple negative breast cancer HCC70 cells with IC_50_ > 500 µM [51].

**Figure 3:**
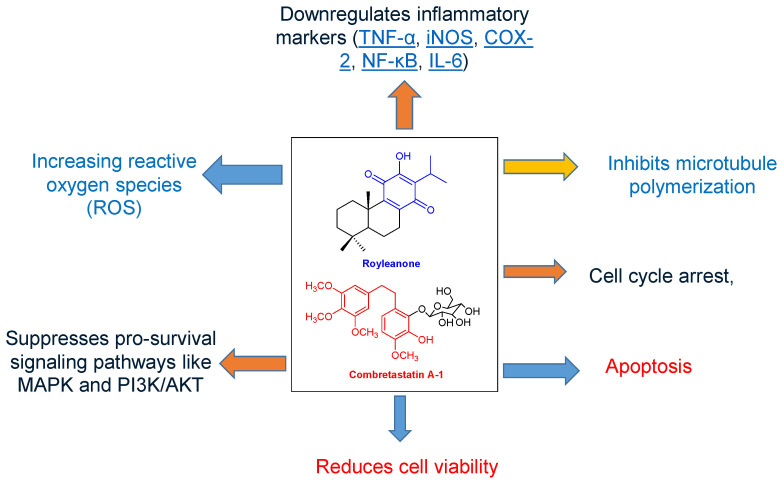
Pictorial representation of possible mechanisms of action of royleanone and combretastatin A-1.

Royleanone diterpenoid is known to exert different mechanisms of action, mainly through P-glycoprotein (P-gp) inhibition, which can improve the effectiveness of chemotherapy drugs in resistant cancer cells. On the other hand, combretastatin can act by targeting and destroying tumor blood vessels, leading to central tumor necrosis, but also by directly inhibiting tumor cell growth and spread. The main mechanism involves binding to tubulin at the colchicine binding site, which destabilizes microtubules and disrupts cell division and integrity [52,53,54] as summarized in Figure 3.

Ferruginol has been reported to induce apoptosis through mitochondrial dysfunction, excessive reactive oxygen species (ROS) generation, and regulation of the BAX/Bcl-2 apoptotic pathway, contributing to its potent cytotoxicity against colorectal cancer cells [55,56]. In androgen-independent prostate cancer PC3 cells, ferruginol further promoted apoptosis by means of caspase activation and nuclear translocation of apoptosis-inducing factor (AIF), alongside inhibition of Ras/PI3K, STAT 3/5, and protein kinase signaling pathways [57].

Royleanone demonstrated cytotoxic effects against LNCaP prostate cancer cells (IC_50_ = 12.5 μM), primarily by inducing cell cycle arrest, triggering mitochondrial-mediated apoptosis and suppressing migration potential through inhibition of the mTOR/PI3K/AKT signaling pathway [58]. Combretastatins and their glucosides act as classic tubulin-binding agents, targeting the colchicine-binding site on the *β*-subunit of tubulin. This prevents tubulin polymerization, induces G_2_/M cell cycle arrest, and activates apoptosis [59].

In contrast, *β*-amyrin palmitate, though less potent, contributes through documented anti-inflammatory and cytotoxic activities, which may act synergistically within crude extracts [25]. Stigmasterol, a common phytosterol identified in many medicinal plants, exerts broad anticancer effects by inducing apoptosis and autophagy, enhancing ROS production, and downregulating PI3K/Akt/mTOR and JAK/STAT pathways, all of which are critical in cancer cell survival and proliferation [60].

Overall, the crude extracts exhibited broader but weaker cytotoxicity compared to purified compounds, likely due to complex synergistic and antagonistic interactions among multiple phytochemicals. Purified isolates, however, demonstrated lower LC_50_ values and more targeted mechanisms of action, underscoring their promise as lead anticancer agents. Future studies should further validate these findings through functional assays focused on ROS generation, mitochondrial integrity, caspase activation, and tubulin interactions.

## 3. Materials and Methods

### 3.1. General Experimental Procedure

Column chromatography was performed on polyamide columns (Sigma-Aldrich) over silica gel (Kieselgel 60, Macherey-Nagel, Sigma-Aldrich). TLC analysis was carried out on a pre-coated aluminum-packed silica plates (ALUGRAM Xtra SIL G/UV 254 20 cm × 20 cm) and viewed under the UV light at wavelengths 254 and 366 nm. The 1 and 2-dimensional NMR experiments were performed using a Varian 400 MHz premium-shielded spectrometer (400/54/ASP, Concord, MA, USA). The CDCI_3_ solvent chemical shift in the ^1^H NMR was recorded as δ 7.2 ppm and that of ^13^C NMR as δ 77.0 ppm. The spectral data obtained from the NMR was interpreted for structural elucidation. A quadrupole time-of-flight (Q-TOF) mass spectrometer (Bruker Daltonics Compact, Billerica, MA, USA) was used for the determination of molecular ions of compounds. A Two Fourier transform infrared (FT-IR) spectrometer with attenuated total reflectance (ATR) transmission (Perkin Elmer, Shelton, CT, USA) was used for chemical profiling of pure compounds. Melting point was determined by the SMP 20 apparatus (Stuart^®^ SMP 20, Cole-Parmer, Staffordshire, UK) operated at 75 W and 50 Hz. The Jasco P-2000 polarimeter (JASCO, Tokyo, Japan) was used for the determination of the specific optical rotation, [α]^D^. The cytotoxicity of pure compounds was determined by using the MTT colorimetric assay.

### 3.2. Plants Samples

Mature *C. glabrum* and *C. nelsonii* were purchased from Wild Harvest nursery in Hartbeespoort, North West Province, South Africa. The plants were identified and the specimen were deposited at the South African National Biodiversity Institute (SANBI) under the voucher number 18044. The roots were cut from each plant species, washed, rinsed with water to remove soil, and air-dried for 3–4 weeks in the laboratory. The roots were then crushed into small pieces and milled into a fine powder using a Polymix^®^ PX-MFC 90D mill (Kinematica, Waltham, MA, USA). The resulting mass of the fine powdered plant material was 1602 g for *C. glabrum* and 1389 g for *C. nelsonii*.

### 3.3. Extraction and Isolation of Compounds

**Figure 4:**
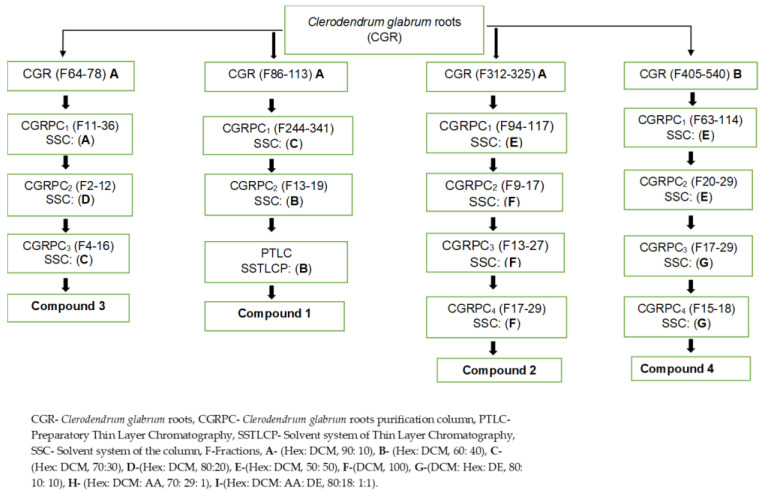
Overview of isolation procedure of compounds from *C. glabrum* roots.

**Figure 5:**
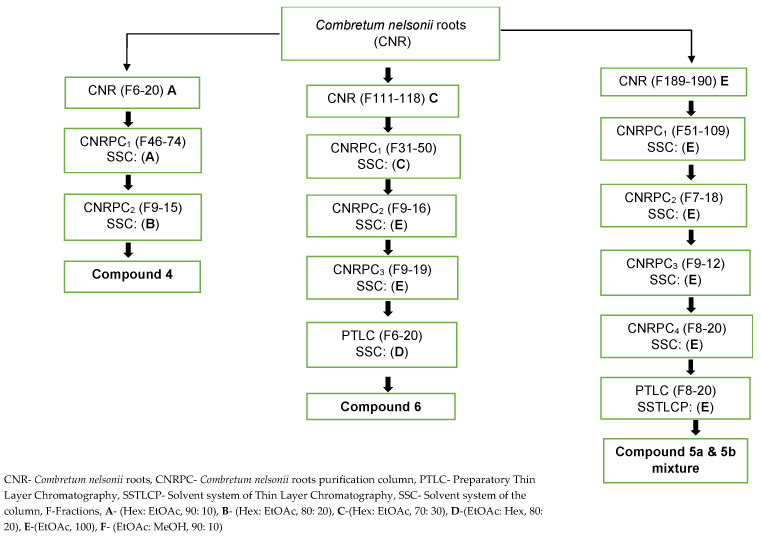
Overview of isolation procedure of compounds from *C. nelsonii* roots.

A sequential extraction method [53] was used to extract crude samples from the roots of *C. glabrum* and *C. nelsonii*. Fractionation of 18.65 g of dried extract of *C. glabrum* was then performed using column chromatography on silica gel and eluted using solvent system of increasing polarity, Hex: DCM (90:10) A, (60:40) B, (50:50) C, (10:90) D and 100% DCM E (*v*/*v*). All fractions were collected in a 100 mL beaker and left in the fume hood to dry. The figures below clearly show the process of obtaining all compounds isolated, Figure 4 (*C. glabrum*) and Figure 5 (*C. nelsonii*).

### 3.4. Cytotoxicity Assay

The cytotoxic activity of extracts and isolated compounds was evaluated using the MTT assay against colorectal adenocarcinoma (Caco-2, ATCC^®^ HTB-37™), breast adenocarcinoma (MCF-7, ATCC^®^ HTB-22™), and non-cancerous monkey kidney cells (Vero, ATCC^®^ CCL-81™). Cell culture and maintenance were carried out following standard protocols [54]. The cells were seeded in 96-well plates and exposed to varying concentrations of extracts (0.025–1 mg/mL) and compounds (0.2–200 µg/mL) for 48 h. Doxorubicin was used as a positive control, and DMSO as a negative control. Cell viability was determined by MTT reduction and absorbance was measured at 570 nm. LC_50_ values were calculated, and the selectivity index(SI) was obtained by dividing LC_50_ values of Vero cells by those of the cancer cell lines.

## 4. Conclusions

The phytochemical investigation of *C. glabrum* and *C. nelsonii* led to the isolation of ferruginol, royleanone, β-amyrin-palmitate, combretastatin A-1, a mixture of combretastatin A-1-2′-*O*-*β*-D-glucopyranoside and combretastatin B-1-2′-*O*-*β*-D-glucopyranoside. Stigmasterol was isolated from both plant species. Ferruginol and royleanone were isolated for the first time from the roots of *C. glabrum*. *C. glabrum* extracts were found to possess good anticancer properties with the SI > 1. Ferruginol was cytotoxic to both Caco-2 and MCF-7 cell lines while royleanone and stigmasterol showed no selective activity. This is an indication that royleanone has minimal potential to be a good anticancer agent against the tested cells. *C. nelsonii* is not fully investigated. Since *C. nelsonii* is not fully investigated, this study provides new information showing that combretastatin A-1 displayed moderate toxicity against MCF-7 cancer cells and could be further explored and structurally modified. Similarly, the mixture of combretastatin A-1-2′-*O*-*β*-D-glucopyranoside and combretastatin B-1-2′-*O*-*β*-D-glucopyranoside exhibited moderate toxicity against MCF-7 cancer cells. The cytotoxicity studies and isolation of compounds supports the reported phytochemical studies and biological activities on the genera *Combretum* and *Clerodendrum*. Mechanistic insights help explain these differential activities.

Overall, this study expands the chemical and biological understanding of Clerodendrum and Combretum species and underscores their potential as reservoirs of anticancer lead compounds warranting further pharmacological and structural optimization.

## Figures and Tables

**Table 1:** Cytotoxicity and selectivity index of compounds and extracts against mammalian cells using MTT assay.

Compounds	Compound Name	Vero (LC_50_) µg/mL	Caco-2 (LC_50_) µg/mL	MCF-7 (LC_50_) µg/mL	Selectivity Index	
					Caco-2	MCF-7
**1**	Ferruginol	2.00 × 10^−4^	24.3	48.4	8.23 × 10^−6^	4.13 × 10^−6^
**2**	Royleanone	30.7	>2.00 × 10^2^	>2.00 × 10^2^	<0.150	<0.150
**3**	β-amyrin palmitate	* ND	* ND	* ND	* ND	* ND
**4**	Combretastatin A-1	15.3	* ND	72.0	* ND	0.210
**5a**	Combretastatin A-1-2′-*O*-*β*-D-glucopyranoside	83.8	* ND	44.1	* ND	1.90
**5b**	Combretastatin B-1-2′-*O*-*β*-D-glucopyranoside					
**6**	Stigmasterol	137	>2.00 × 10^2^	>2.00 × 10^2^	<0.690	<0.690
**Extracts**						
DCM	*C. glabrum*	4.30 × 10^3^	1.30 × 10^3^	2790	3.31	1.54
DCM	*C. nelsonii*	1.70 × 10^3^	2390	6610	0.710	0.260
EtOAc	*C. nelsonii*	117	* ND	252	* ND	0.460
Positive control	Doxorubicin	6.90	0.760	3.10	9.08	2.20

* ND: Not determined.

**Table 2:** Comparative cytotoxicity of extracts and isolated compounds from *C. glabrum* and *C. nelsonii* versus the literature reports.

Compound Name/ Extract	Cell Line (LC_50_) µg/mL	Selectivity Index	Literature Report (Cell Line & IC_50_/GI_5_)	References
Ferruginol	Caco-2: 24.3 µg/mL; MCF-7: 48.4 µg/mL; Vero: 0.0002 µg/mL	Low SI (8.23 × 10^−6^, and 4.13 × 10^−6^), highly toxic to Vero, moderate to MCF-7	MCF-7: 12 µM; AGS: 27 µM; HepG2: 68.5 µM; PC3: 55 µM; colon (COL-2): ED_50_ > 20 µg/mL	[39,42,43,44]
Royleanone	Caco-2, MCF-7: LC_50_ > 200 µg/mL	SI < 0.15, inactive	Prostate (LNCaP): 12.5 µM; AGS: 18 µM; breast MDA-MB-231: >250 µM; colon HCT116: variable	[45,46,47]
Combretastatin A-1	Vero: 15.3 µg/mL; MCF-7: 72.0 µg/mL	Moderately cytotoxic to MCF-7, highly toxic to Vero	Potent tubulin inhibitor IC_50_: 1.1 µM; BXPC-3: GI_50_ = 4.4 µg/mL; DU-145: GI_50_ = 0.017 µg/mL; P388: GI_50_ = 0.3 µg/mL; KM20L2: GI_50_ = 0.061 µg/mL; NCI-H460: GI_50_ = 0.74 µg/mL	[48,49,50]
Stigmasterol	Caco-2, MCF-7: >200 µg/mL; Vero: 137 µg/mL	Low SI, inactive	SW620: IC_50_ = 2.79 µM; Caco-2: 132.5 ± 33.3 µM; MCF-7 and HCC70: >500 µM	[51]
Combretastatin A-1-2′-*O*-β-D-glucopyranoside 5a and Combretastatin B-1-2′-*O*-β-D-glucopyranoside 5b	MCF-7: 44.1 µg/mL; Vero: 83.8 µg/mL	SI = 1.90, selectively toxic to cancer cells	Moderate cytotoxicity; promising for selective activity in breast cancer therapy	This study
*C. glabrum* DCM extract	MCF-7: 2790 µg/mL; Caco-2: 1300 µg/mL	SI: 1.54 (MCF-7), 3.31 (Caco-2) Selectively toxic to cancer cells	No prior report on MCF-7; *C. villosum & C. indicum* active against SW620: IC_50_ > 200 µg/mL	[40]
*C. nelsonii* EtOAc	MCF-7: 252 µg/mL	SI = 0.46, weak cytotoxicity	*C. rupicola* leaf extract: IC_50_ = 65.9 µg/mL (MCF-7)	[41]

## Data Availability

The original data presented in the study are openly available in repository as supplementary materials.

## Supplementary Materials

The following supporting information can be downloaded at: https://www.mdpi.com/article/10.3390/plants14182832/s1, Supplementary Figure S1 to Supplementary Figure S40 and Supplementary Table S1 to Supplementary Table S3.

## Abbreviations

The following abbreviations are used in this manuscript:

Caco-2	Colorectal adenocarcinoma
MCF-7	Hormone receptor-positive breast cancer
NMR	Nuclear magnetic resonance
WHO	World Health Organization
MTT	3-(4,5-dimethylthiazol-2-yl)-2,5-diphenyl tetrazolium bromide
DCM	Dichloromethane
EtOAc	Ethyl acetate
HepG2	Human liver hepatocellular carcinoma
COL-2	Colon cancer
DMSO	Dimethyl sulfoxide
SI	Selectivity index
LC_50_	50% Lethal concentration
