# Supplementary material for: Cytotoxic Activity of Chemical Constituents of Clerodendrum glabrum and Combretum nelsonii Root Extracts Against Selected Cancer Cell Lines
Source: Plants (Basel). 2025 Sep 11;14(18):2832. doi: 10.3390/plants14182832 (PMC12473540; doi:10.3390/plants14182832)
Supplement: Supplementary file 1 [file plants-14-02832-s001.zip › plants-3807977-Supplementary materials.pdf]

Supplementary Materials

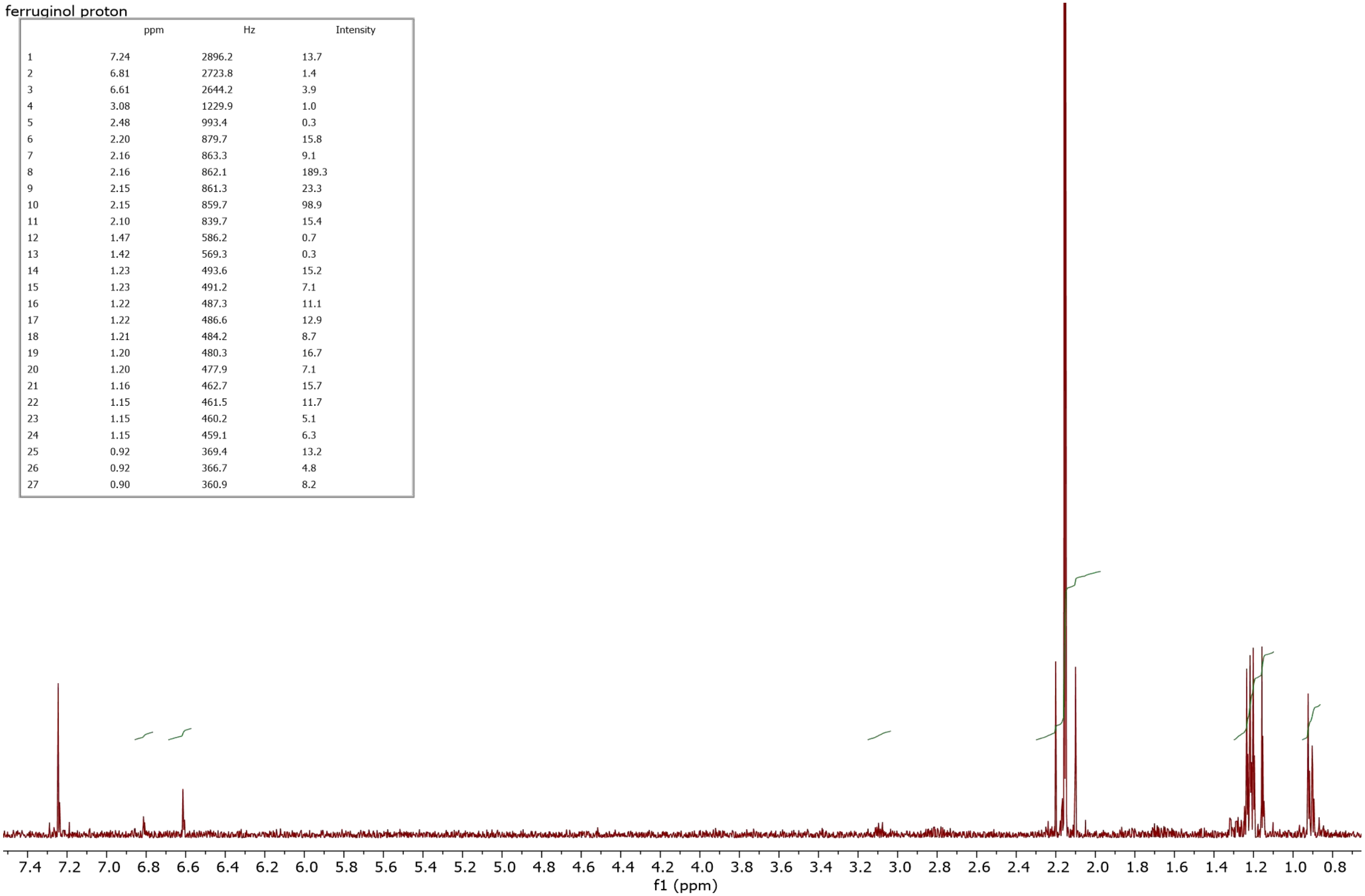

ferruginol proton

	ppm	Hz	Intensity
1	7.24	2896.2	13.7
2	6.81	2723.8	1.4
3	6.61	2644.2	3.9
4	3.08	1229.9	1.0
5	2.48	993.4	0.3
6	2.20	879.7	15.8
7	2.16	863.3	9.1
8	2.16	862.1	189.3
9	2.15	861.3	23.3
10	2.15	859.7	98.9
11	2.10	839.7	15.4
12	1.47	586.2	0.7
13	1.42	569.3	0.3
14	1.23	493.6	15.2
15	1.23	491.2	7.1
16	1.22	487.3	11.1
17	1.22	486.6	12.9
18	1.21	484.2	8.7
19	1.20	480.3	16.7
20	1.20	477.9	7.1
21	1.16	462.7	15.7
22	1.15	461.5	11.7
23	1.15	460.2	5.1
24	1.15	459.1	6.3
25	0.92	369.4	13.2
26	0.92	366.7	4.8
27	0.90	360.9	8.2

Figure S1: ^{1}H NMR spectrum of compound 1: 12-hydroxy-abieta-8,11,13-triene (Ferruginol)

ferruginol carbon

	ppm	Hz	Intensity
1	150.71	15148.9	4.4
2	148.61	14938.3	3.8
3	131.37	13205.3	5.2
4	127.19	12785.3	4.9
5	126.57	12722.7	12.5
6	110.93	11150.7	12.1
7	77.29	7769.2	57.8
8	76.97	7737.2	61.1
9	76.65	7705.2	60.8
10	50.33	5059.3	13.4
11	41.67	4188.7	15.7
12	38.84	3904.5	15.0
13	37.47	3766.5	6.4
14	33.40	3357.2	9.4
15	33.26	3343.4	15.3
16	30.86	3101.6	35.6
17	29.71	2986.7	14.4
18	29.66	2981.1	4.3
19	26.78	2691.8	12.5
20	24.73	2486.2	14.2
21	22.69	2281.0	14.7
22	22.51	2263.1	14.5
23	21.57	2168.6	15.4
24	19.28	1938.4	14.8
25	19.20	1930.4	14.7

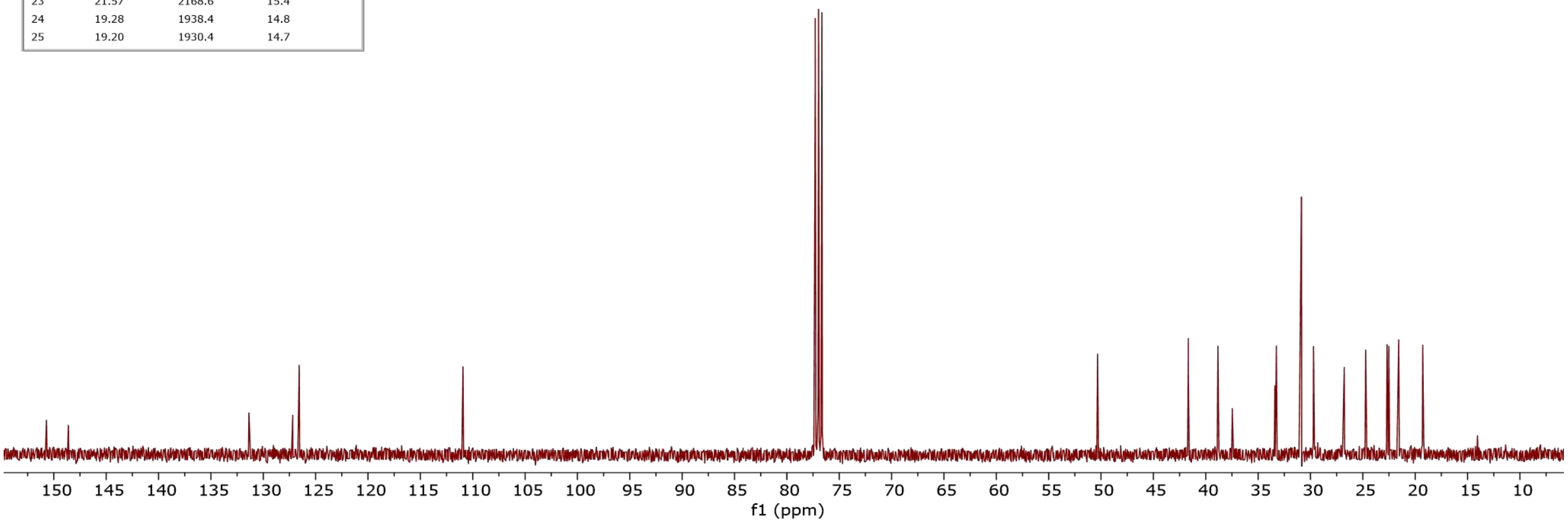

Figure S2: ^{13}C NMR spectrum of compound 1: 12-hydroxy-abieta-8,11,13-triene (Ferruginol)

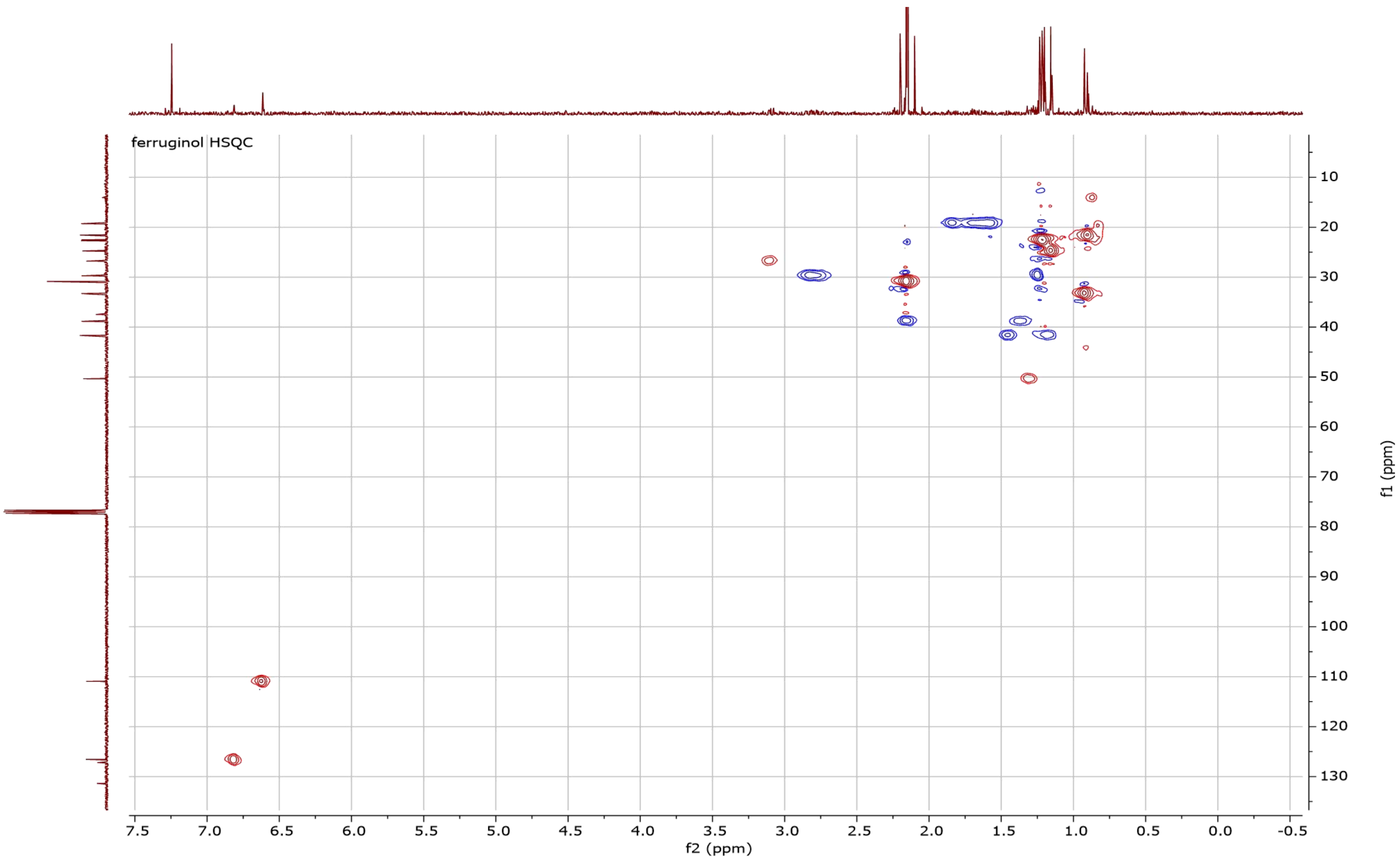

Figure S3: HSQC spectrum of compound 1: 12-hydroxy-abieta-8,11,13-triene (Ferruginol)

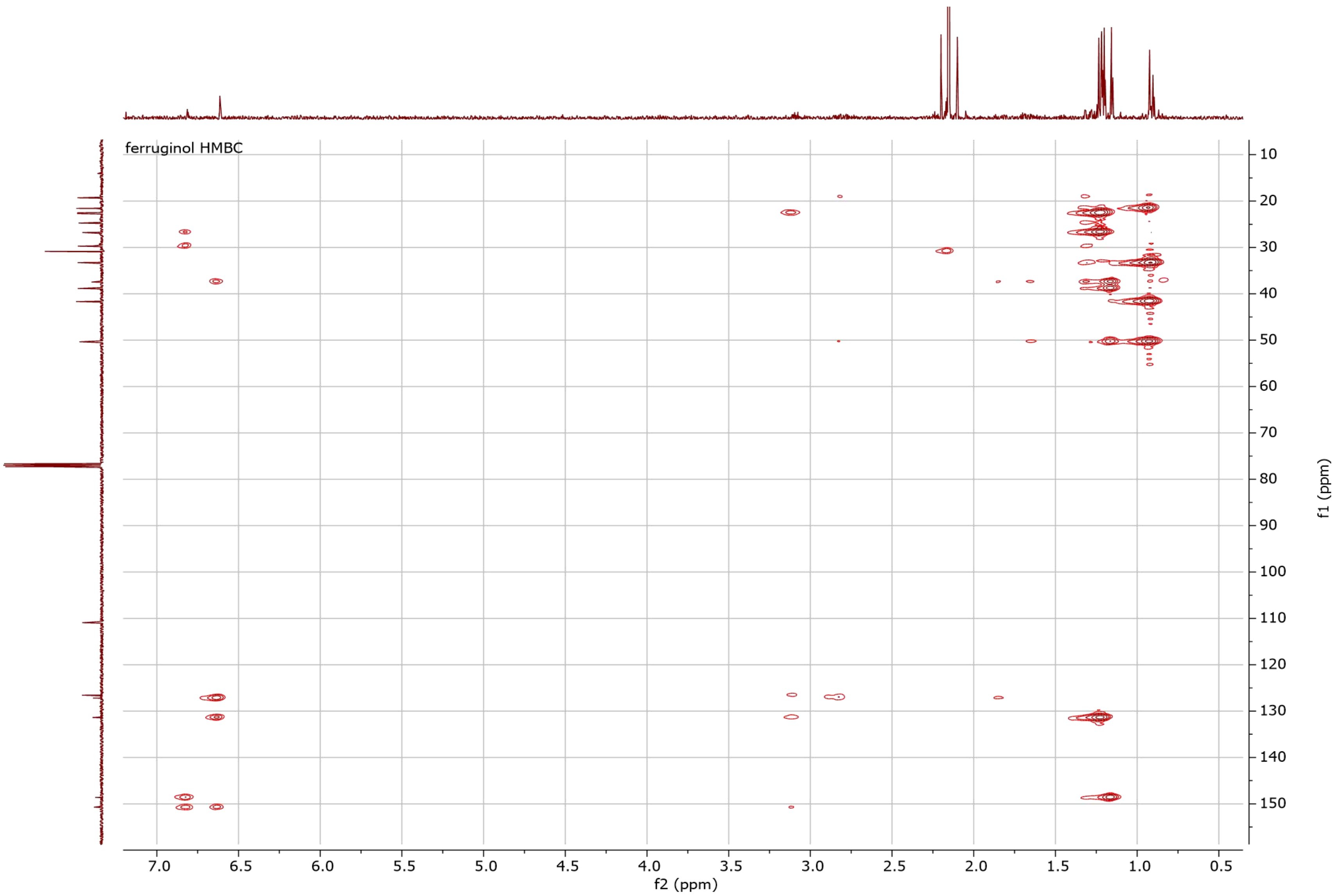

Figure S4: HMBC spectrum of compound 1: 12-hydroxy-abieta-8,11,13-triene (Ferruginol)

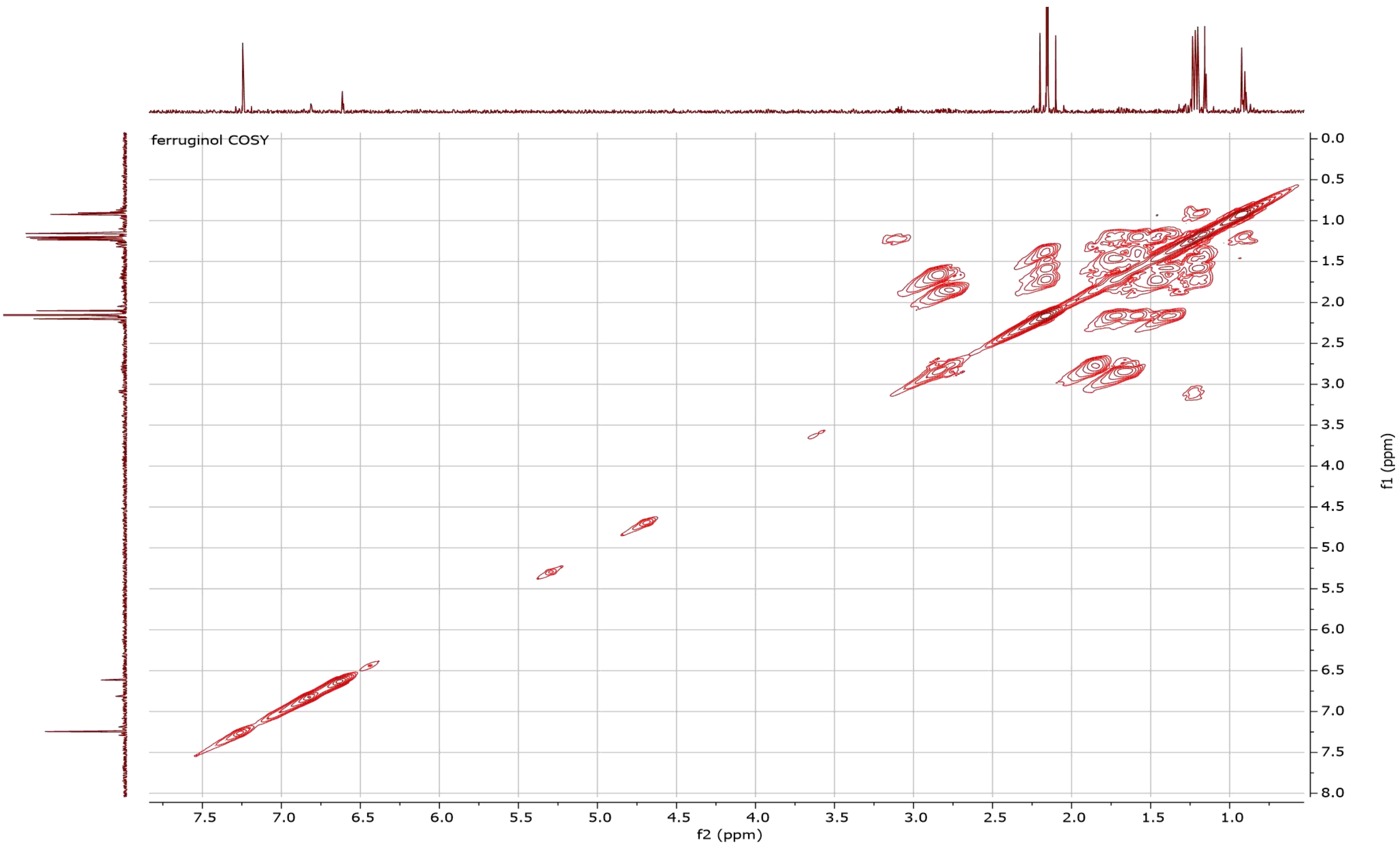

Figure S5: COSY spectrum of compound 1: 12-hydroxy-abieta-8,11,13-triene (Ferruginol)

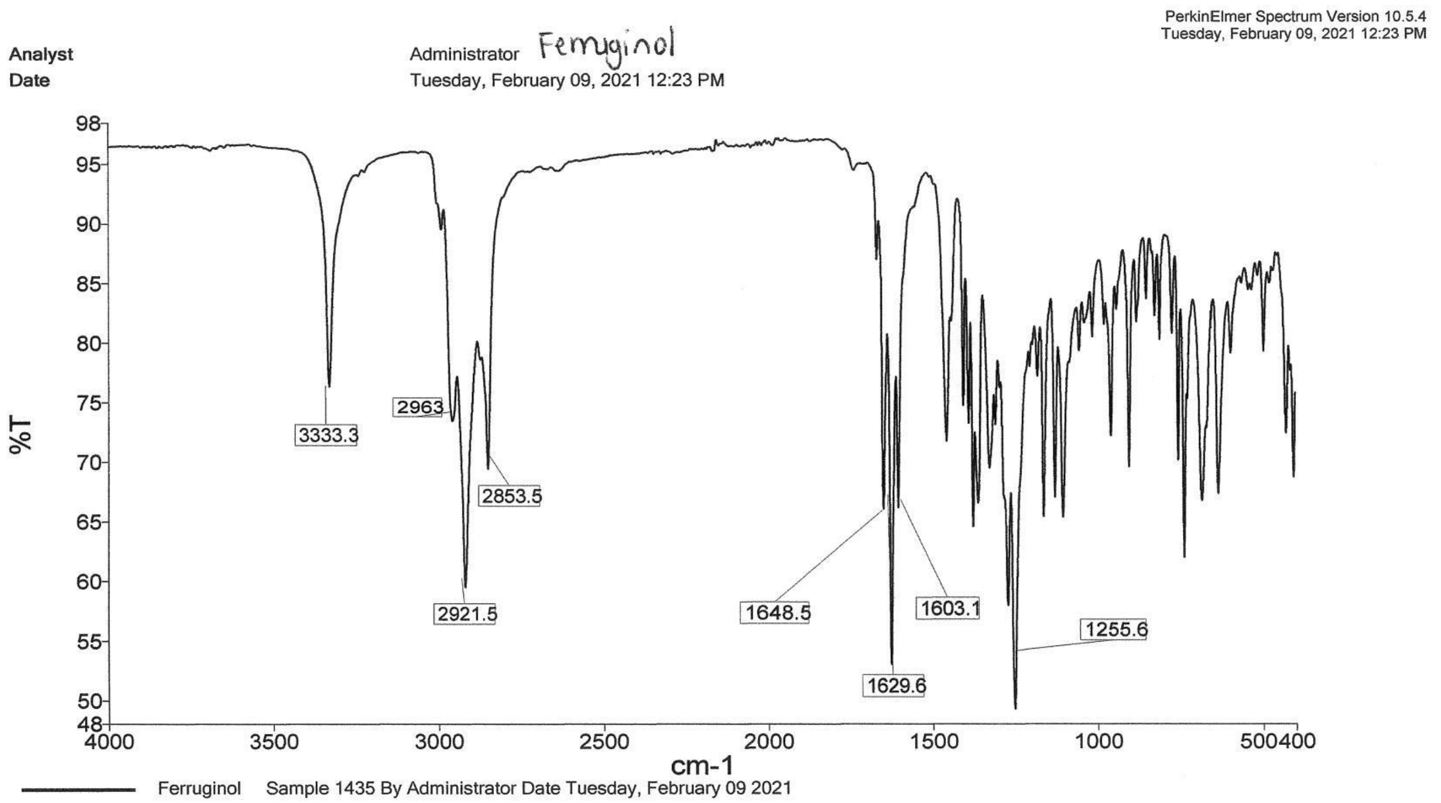

Figure S6: IR spectrum of compound 1: 12-hydroxy-abieta-8,11,13-triene (Ferruginol)

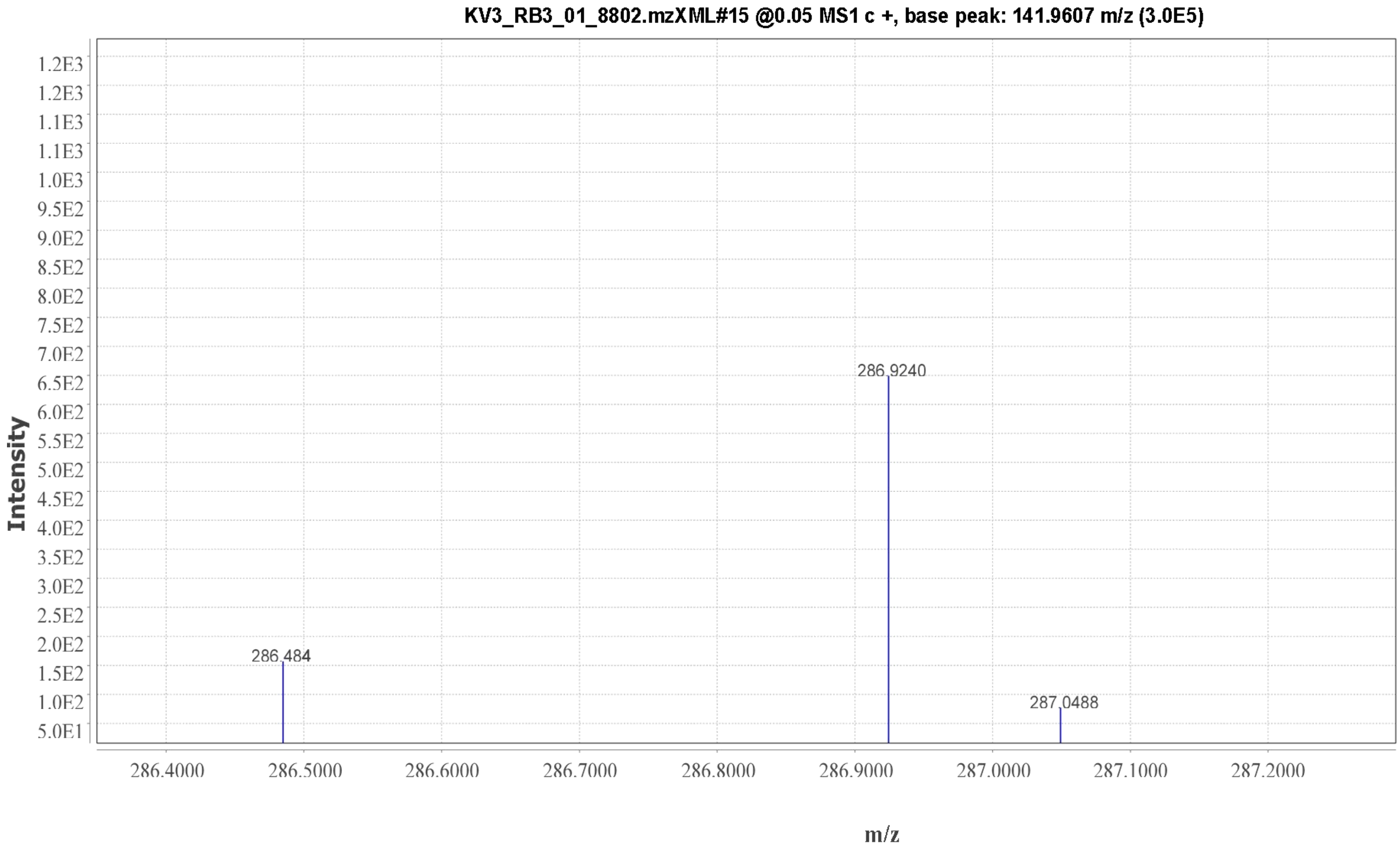

Figure S7: MS spectrum of compound **1**: 12-hydroxy-abieta-8,11,13-triene (Ferruginol)

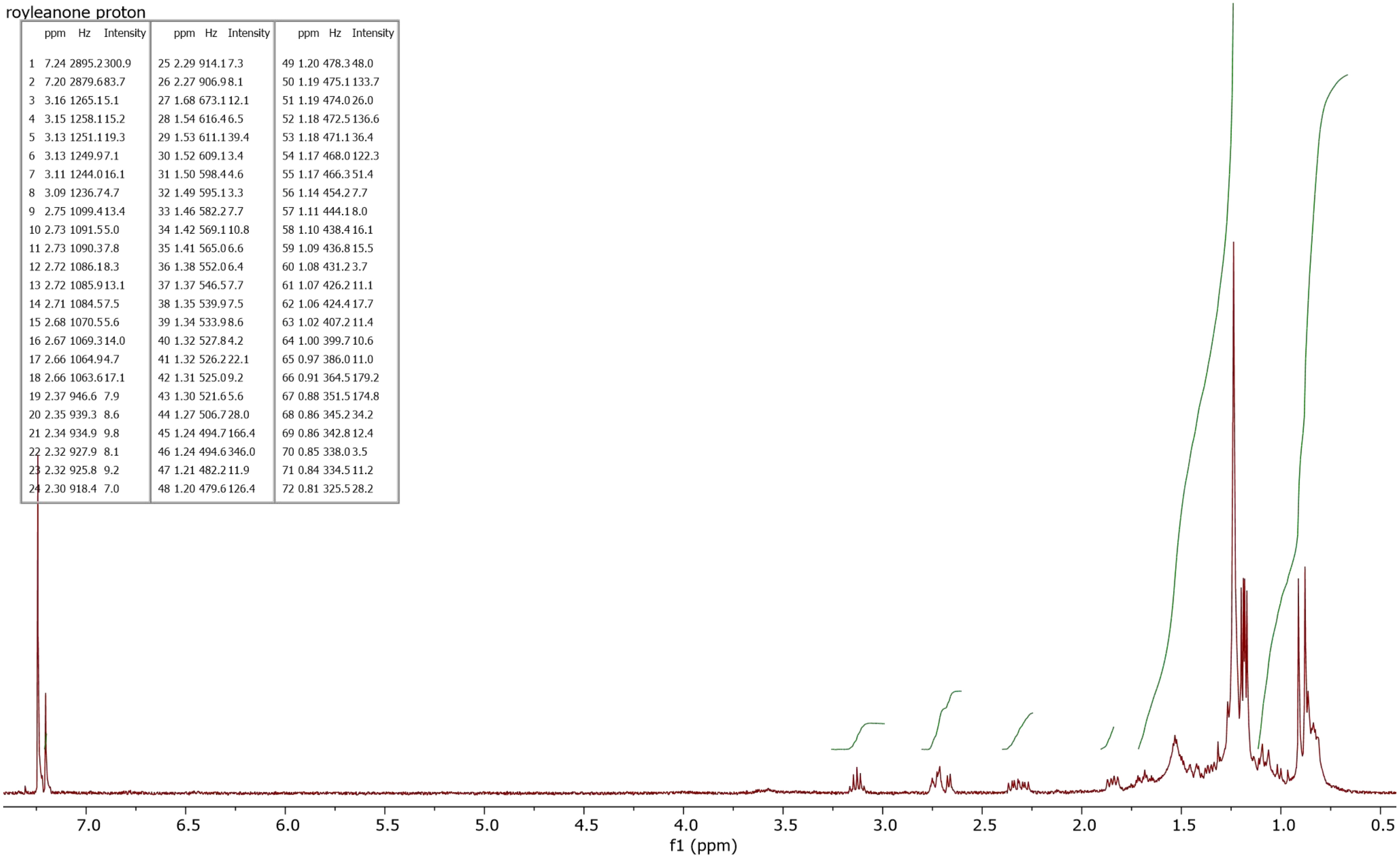

	ppm	Hz	Intensity		ppm	Hz	Intensity		ppm	Hz	Intensity
1	7.24	2895.2	300.9	25	2.29	914.1	7.3	49	1.20	478.3	48.0
2	7.20	2879.6	83.7	26	2.27	906.9	8.1	50	1.19	475.1	133.7
3	3.16	1265.1	5.1	27	1.68	673.1	12.1	51	1.19	474.0	26.0
4	3.15	1258.1	15.2	28	1.54	616.4	6.5	52	1.18	472.5	136.6
5	3.13	1251.1	19.3	29	1.53	611.1	39.4	53	1.18	471.1	36.4
6	3.13	1249.9	7.1	30	1.52	609.1	3.4	54	1.17	468.0	122.3
7	3.11	1244.0	16.1	31	1.50	598.4	4.6	55	1.17	466.3	51.4
8	3.09	1236.7	4.7	32	1.49	595.1	3.3	56	1.14	454.2	7.7
9	2.75	1099.4	13.4	33	1.46	582.2	7.7	57	1.11	444.1	8.0
10	2.73	1091.5	5.0	34	1.42	569.1	10.8	58	1.10	438.4	16.1
11	2.73	1090.3	7.8	35	1.41	565.0	6.6	59	1.09	436.8	15.5
12	2.72	1086.1	8.3	36	1.38	552.0	6.4	60	1.08	431.2	3.7
13	2.72	1085.9	13.1	37	1.37	546.5	7.7	61	1.07	426.2	11.1
14	2.71	1084.5	7.5	38	1.35	539.9	7.5	62	1.06	424.4	17.7
15	2.68	1070.5	5.6	39	1.34	533.9	8.6	63	1.02	407.2	11.4
16	2.67	1069.3	14.0	40	1.32	527.8	4.2	64	1.00	399.7	10.6
17	2.66	1064.9	4.7	41	1.32	526.2	22.1	65	0.97	386.0	11.0
18	2.66	1063.6	17.1	42	1.31	525.0	9.2	66	0.91	364.5	179.2
19	2.37	946.6	7.9	43	1.30	521.6	5.6	67	0.88	351.5	174.8
20	2.35	939.3	8.6	44	1.27	506.7	28.0	68	0.86	345.2	34.2
21	2.34	934.9	9.8	45	1.24	494.7	166.4	69	0.86	342.8	12.4
22	2.32	927.9	8.1	46	1.24	494.6	346.0	70	0.85	338.0	3.5
23	2.32	925.8	9.2	47	1.21	482.2	11.9	71	0.84	334.5	11.2
24	2.30	918.4	7.0	48	1.20	479.6	126.4	72	0.81	325.5	28.2

Figure S8: ^{1}H NMR spectrum of compound 2: 12-hydroxy-8,12-abietadiene-11,14-diol (Royleanone)

Royleanone Carbon

	ppm	Hz	Intensity
1	187.49	18845.8	28.8
2	183.36	18430.7	26.8
3	150.49	15126.7	38.7
4	145.96	14671.3	48.3
5	123.68	12432.5	28.6
6	77.28	7767.7	1154.8
7	76.96	7735.7	1165.7
8	76.64	7703.7	1159.5
9	51.69	5195.9	116.5
10	41.28	4149.0	111.9
11	38.38	3858.4	49.4
12	36.23	3641.7	112.6
13	33.38	3355.6	117.4
14	29.65	2980.7	208.1
15	26.62	2676.2	121.4
16	24.05	2417.2	96.3
17	22.64	2276.1	49.2
18	21.72	2183.0	119.2
19	20.04	2014.4	118.9
20	19.91	2001.7	112.3
21	19.80	1989.9	113.5
22	18.86	1896.1	140.2
23	17.39	1748.5	113.7

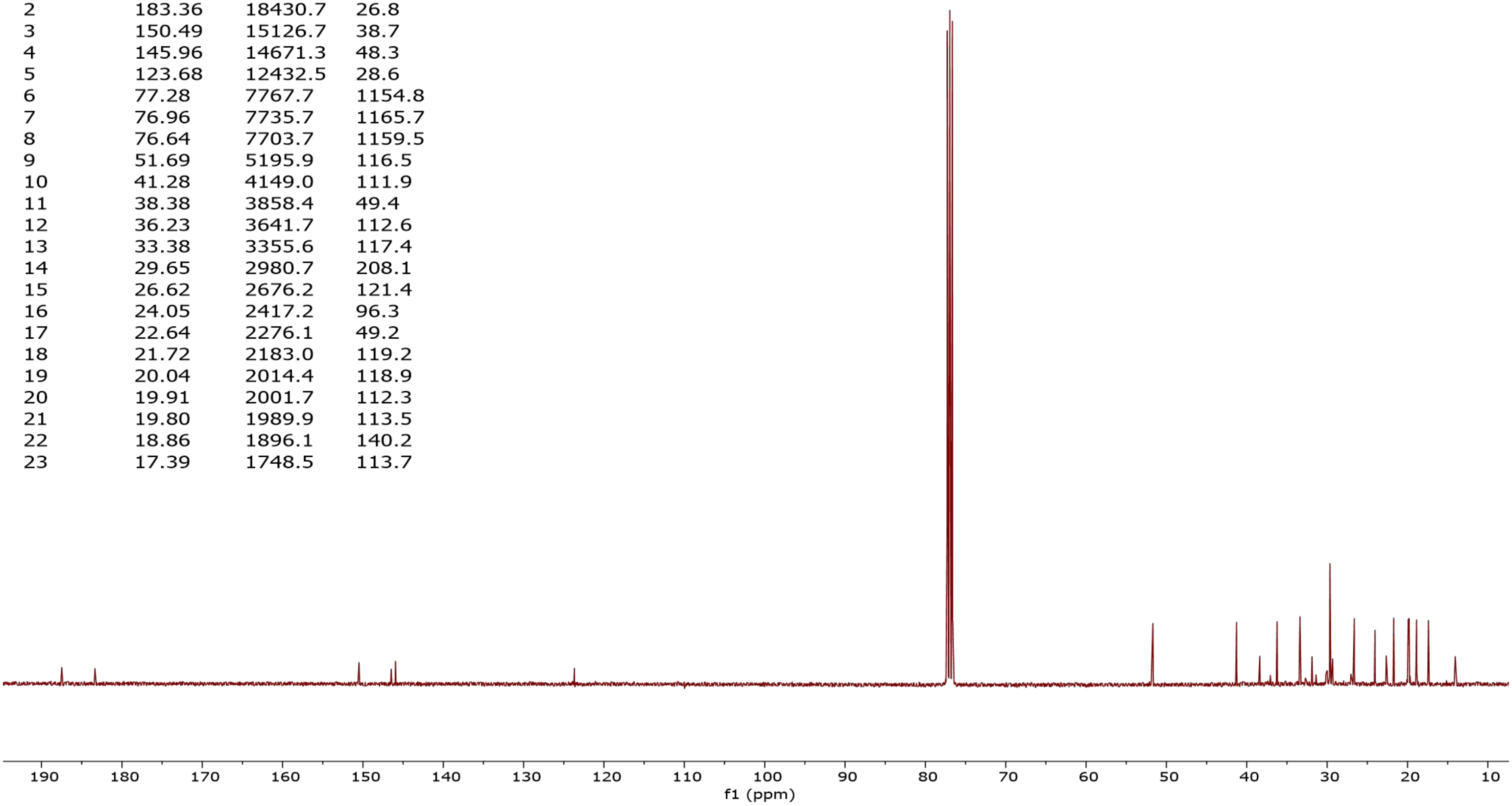

Figure S9: ^{13}C NMR spectrum of compound **2**: 12-hydroxy-8,12-abietadiene-11,14-diol (Royleanone)

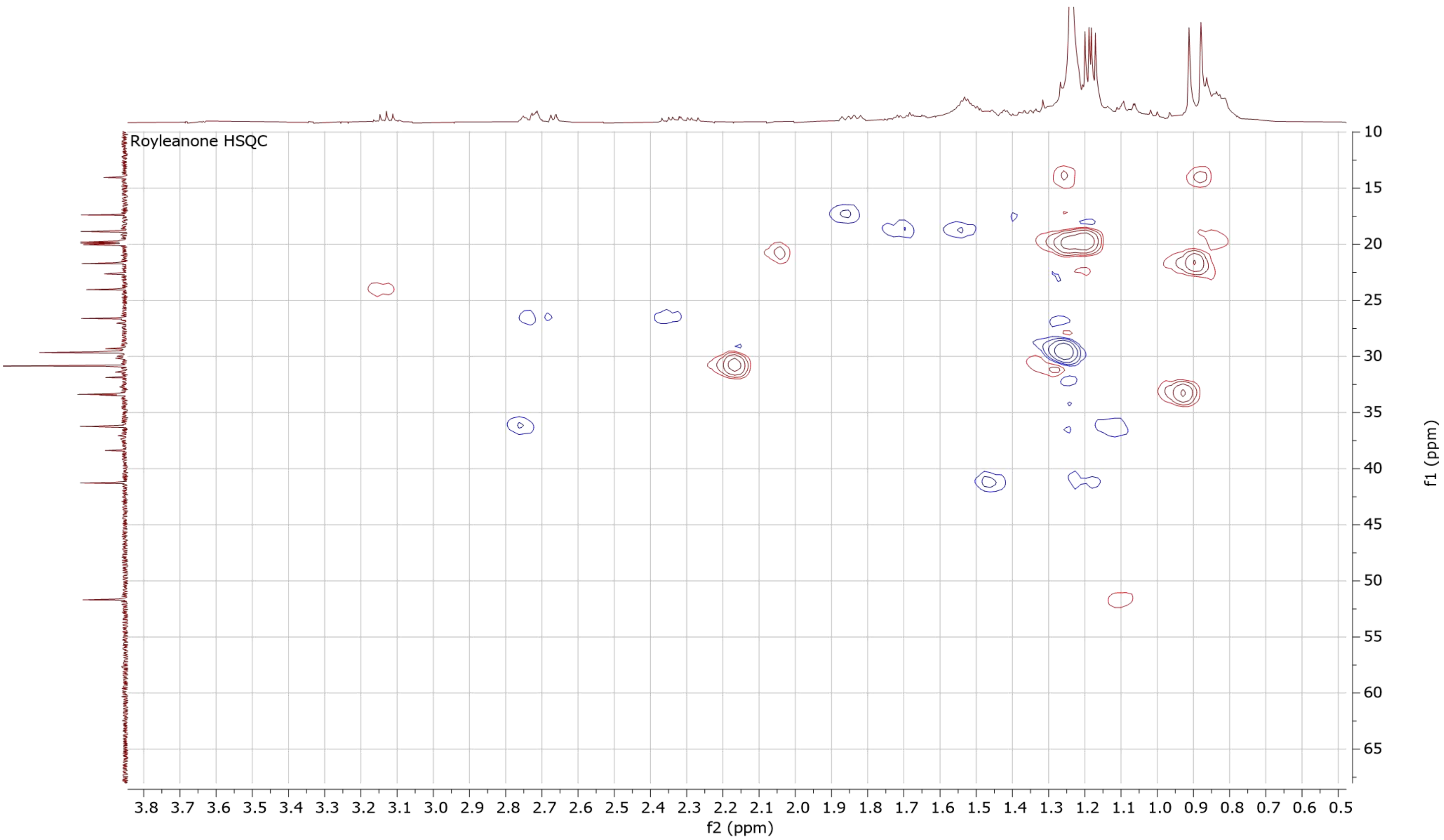

Figure S10: HSQC spectrum of compound 2: 12-hydroxy-8,12-abietadiene-11,14-diol (Royleanone)

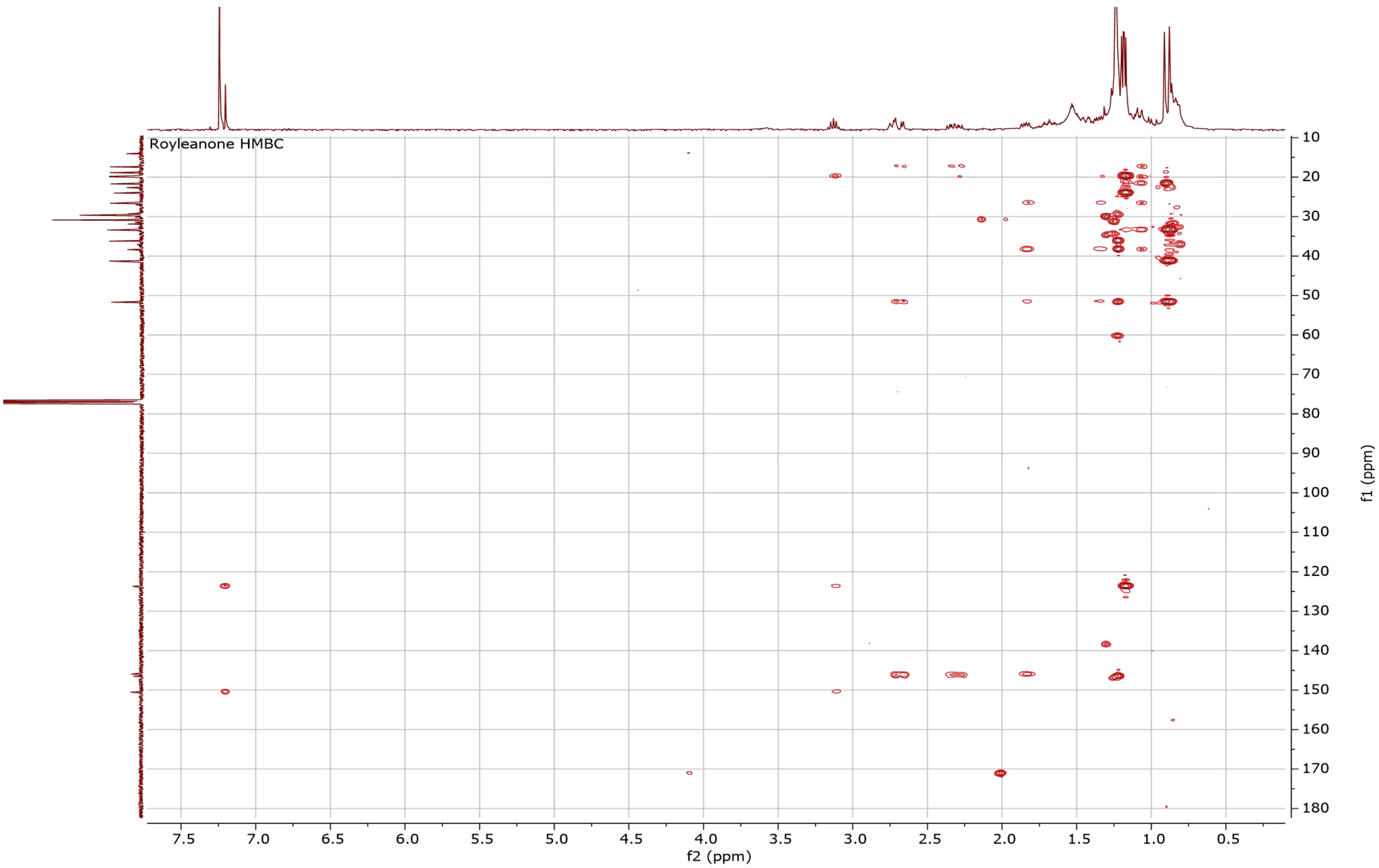

Figure S11: HMBC spectrum of compound 2: 12-hydroxy-8,12-abietadiene-11,14-diol (Royleanone)

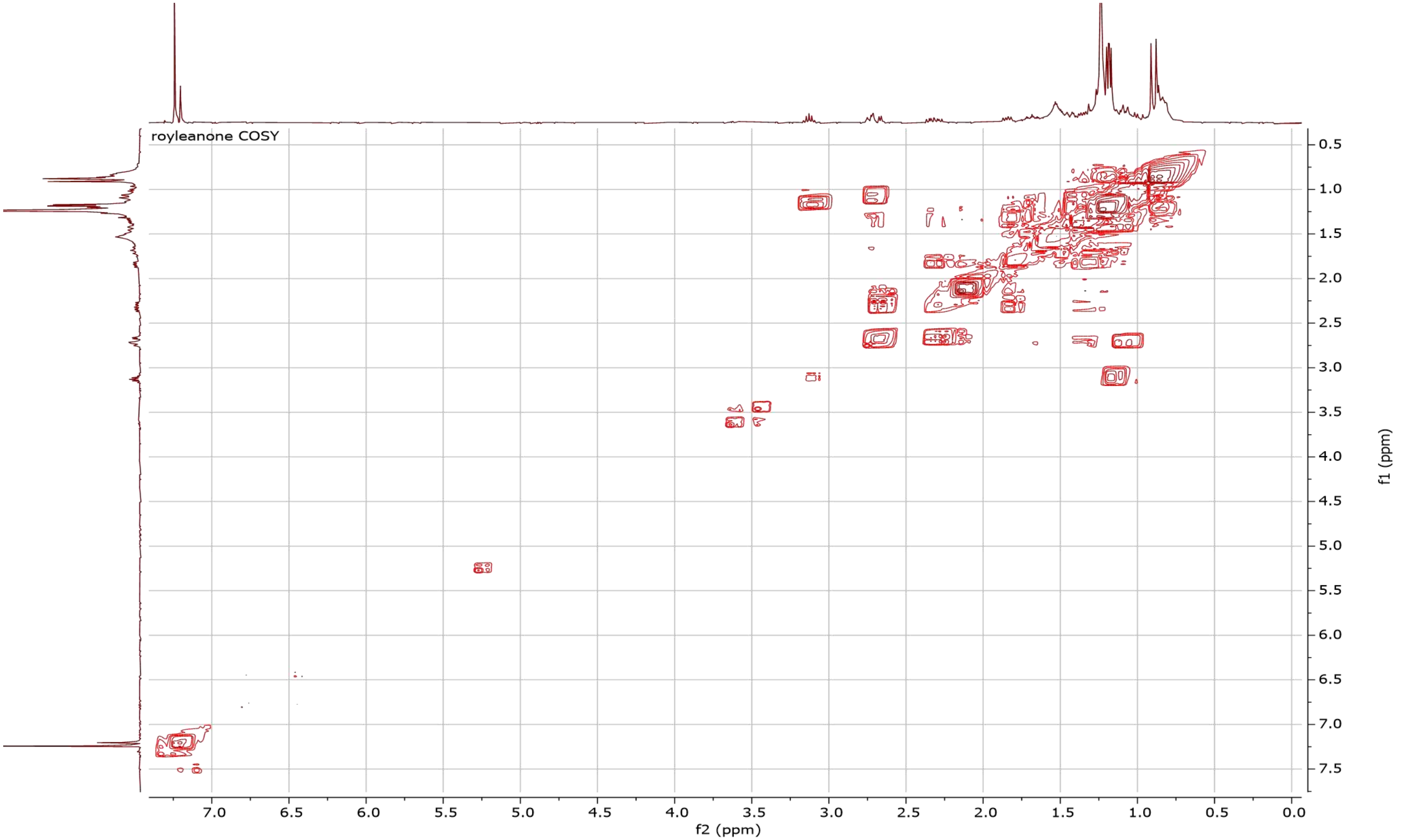

Figure S12: COSY spectrum of compound 2: 12-hydroxy-8,12-abietadiene-11,14-diol (Royleanone)

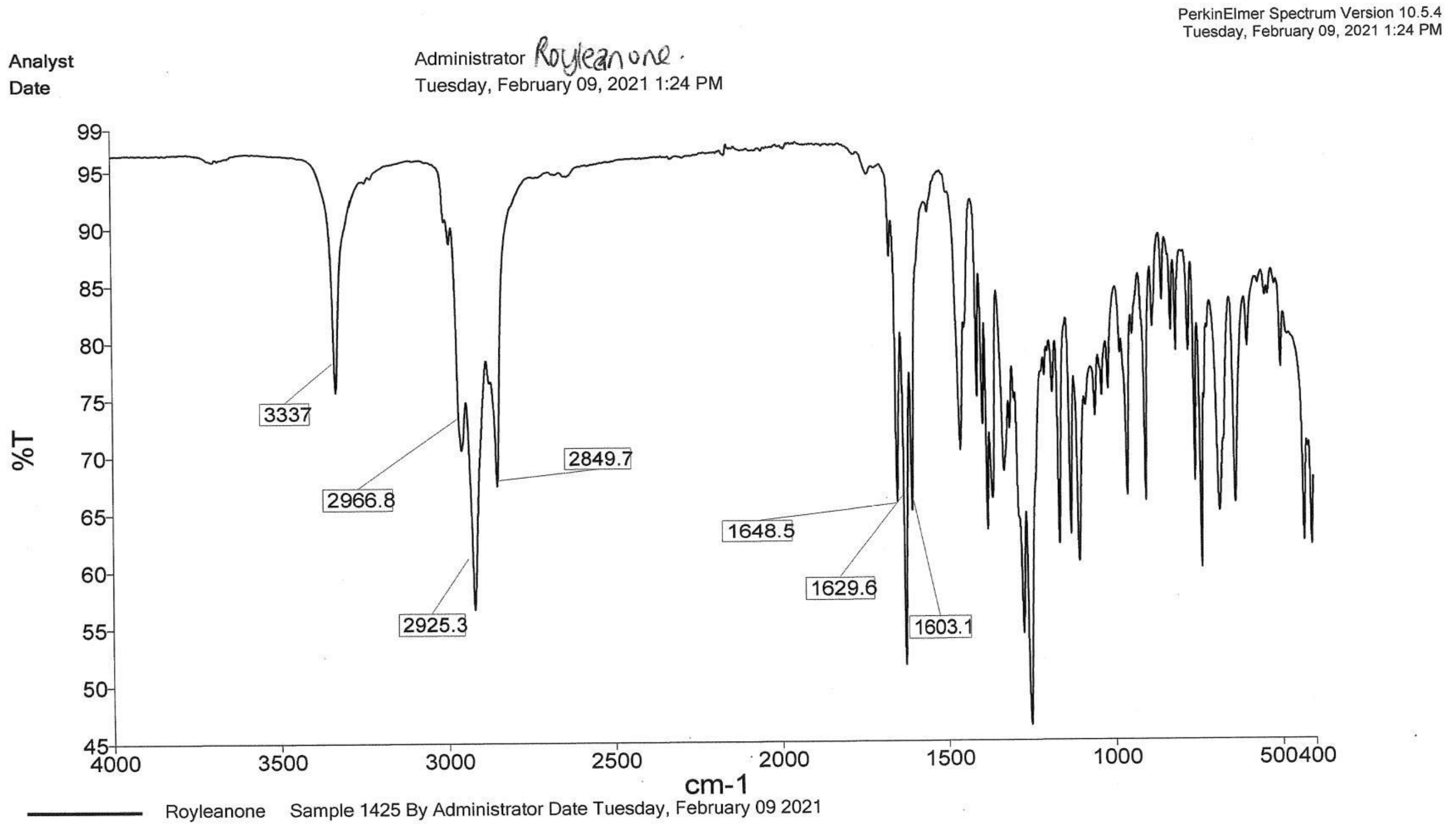

Figure S13: IR spectrum of compound 2: 12-hydroxy-8,12-abietadiene-11,14-diol (Royleanone)

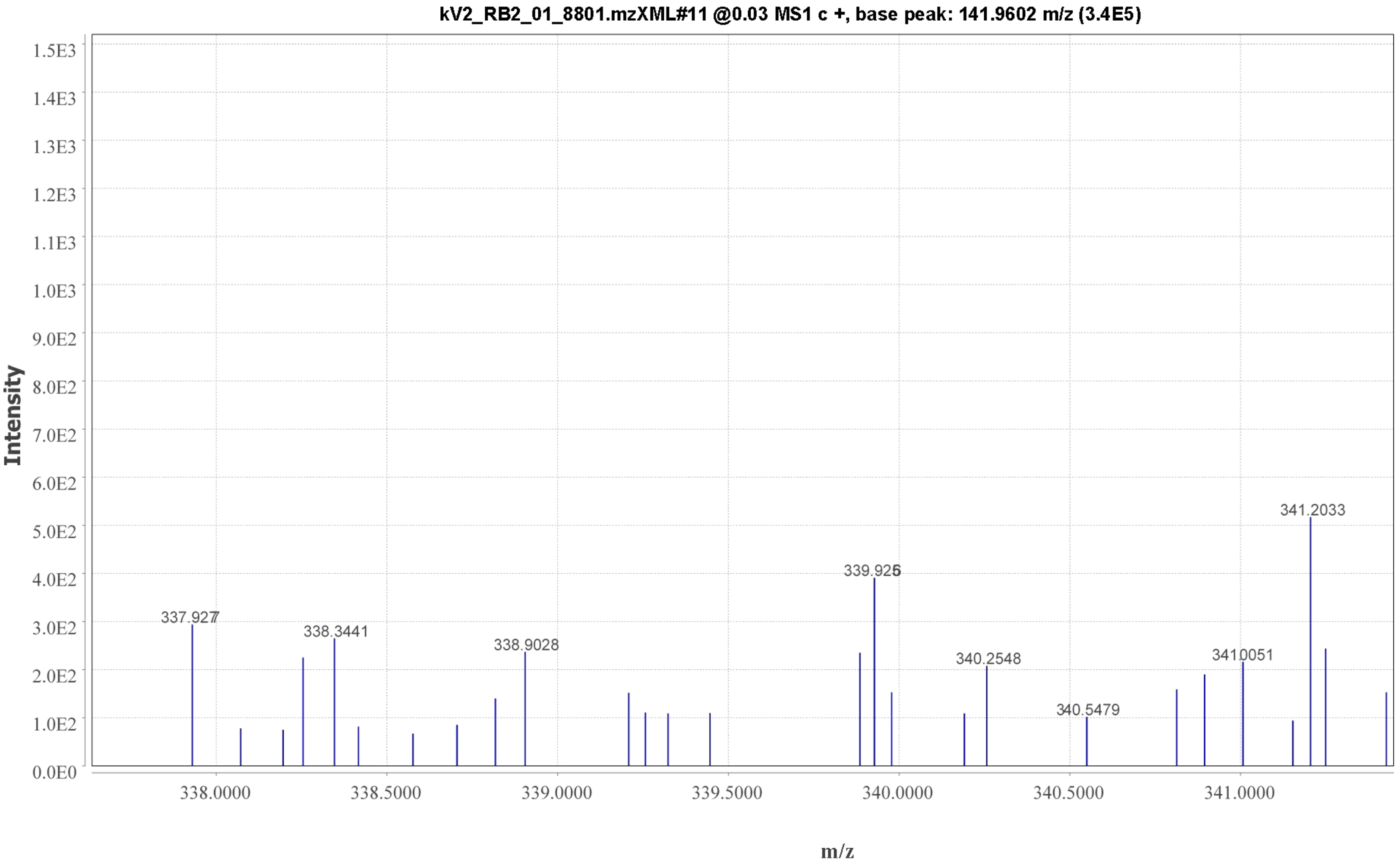

Figure S14: MS spectrum of compound 2: 12-hydroxy-8,12-abietadiene-11,14-diol (Royleanone)

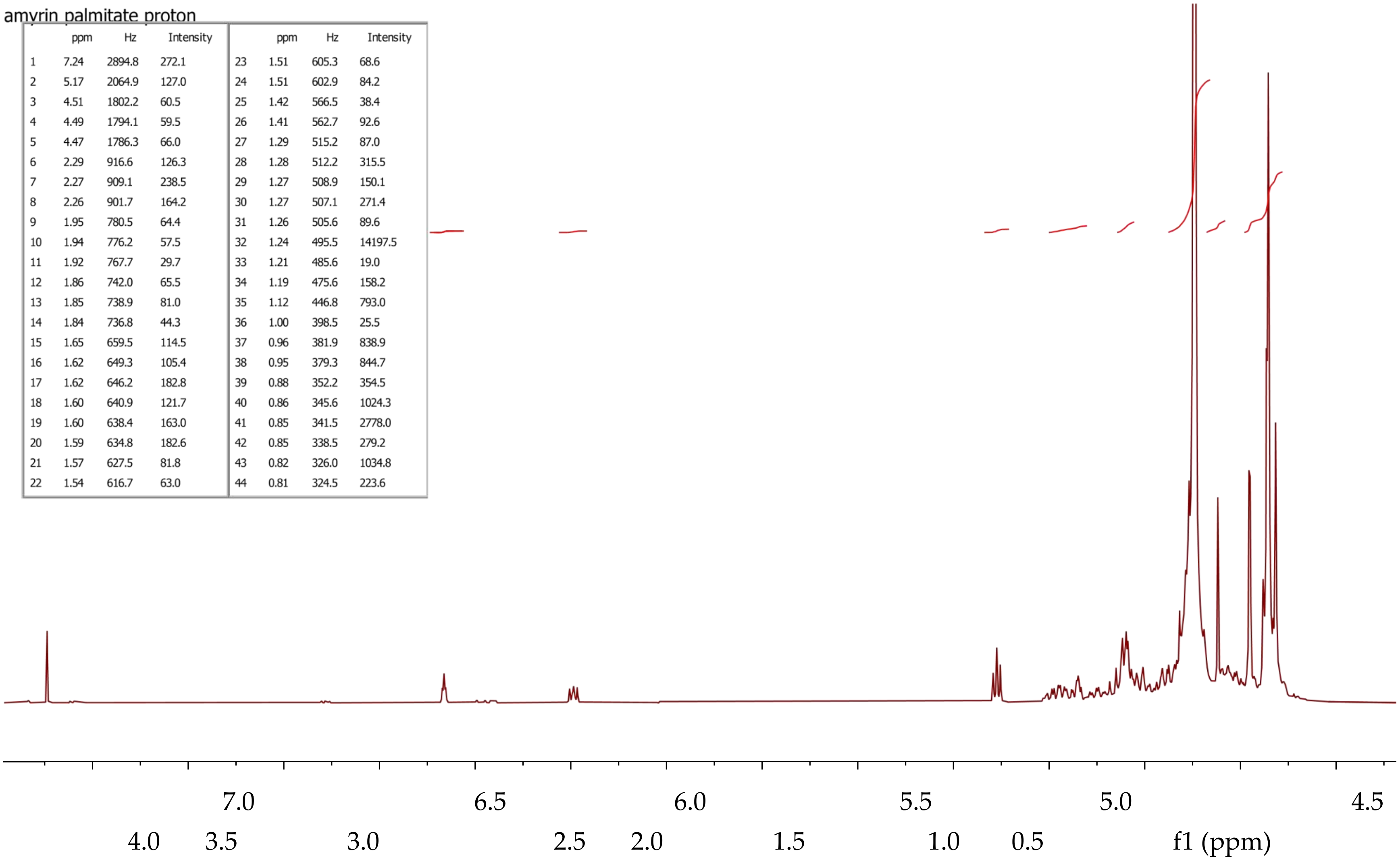

	ppm	Hz	Intensity		ppm	Hz	Intensity
1	7.24	2894.8	272.1	23	1.51	605.3	68.6
2	5.17	2064.9	127.0	24	1.51	602.9	84.2
3	4.51	1802.2	60.5	25	1.42	566.5	38.4
4	4.49	1794.1	59.5	26	1.41	562.7	92.6
5	4.47	1786.3	66.0	27	1.29	515.2	87.0
6	2.29	916.6	126.3	28	1.28	512.2	315.5
7	2.27	909.1	238.5	29	1.27	508.9	150.1
8	2.26	901.7	164.2	30	1.27	507.1	271.4
9	1.95	780.5	64.4	31	1.26	505.6	89.6
10	1.94	776.2	57.5	32	1.24	495.5	14197.5
11	1.92	767.7	29.7	33	1.21	485.6	19.0
12	1.86	742.0	65.5	34	1.19	475.6	158.2
13	1.85	738.9	81.0	35	1.12	446.8	793.0
14	1.84	736.8	44.3	36	1.00	398.5	25.5
15	1.65	659.5	114.5	37	0.96	381.9	838.9
16	1.62	649.3	105.4	38	0.95	379.3	844.7
17	1.62	646.2	182.8	39	0.88	352.2	354.5
18	1.60	640.9	121.7	40	0.86	345.6	1024.3
19	1.60	638.4	163.0	41	0.85	341.5	2778.0
20	1.59	634.8	182.6	42	0.85	338.5	279.2
21	1.57	627.5	81.8	43	0.82	326.0	1034.8
22	1.54	616.7	63.0	44	0.81	324.5	223.6

Figure S15: ^{1}H NMR spectrum of compound 3: 3β-olean-12-en-3-yl-palmitate (β-amyrin-palmitate)

amyrin-palmitate Carbon

	ppm	Hz	Intensity		ppm	Hz	Intensity		ppm	Hz	Intensity
1	173.67	17456.5	37.3	23	34.85	3502.7	61.3	45	28.03	2818.0	65.7
2	145.19	14594.0	33.0	24	34.71	3489.3	52.6	46	27.07	2721.1	24.5
3	121.63	12225.8	57.7	25	33.31	3347.9	62.7	47	26.91	2704.9	59.1
4	80.55	8096.7	66.1	26	32.74	3290.8	17.1	48	26.72	2685.7	13.5
5	77.30	7770.0	477.3	27	32.57	3274.2	57.2	49	26.12	2625.2	55.7
6	77.25	7765.2	144.6	28	32.47	3263.8	49.6	50	25.94	2607.1	62.8
7	77.01	7741.1	34.6	29	31.94	3210.6	21.7	51	25.16	2529.1	64.7
8	76.98	7737.9	484.9	30	31.91	3207.4	164.5	52	23.70	2381.8	6.9
9	76.93	7733.1	141.7	31	31.06	3122.2	51.2	53	23.67	2379.1	63.8
10	76.69	7709.0	79.6	32	30.15	3030.6	20.8	54	23.59	2371.2	53.8
11	76.66	7706.1	463.9	33	30.02	3017.9	29.4	55	23.52	2363.8	55.0
12	76.62	7701.7	187.5	34	29.69	2984.0	1414.6	56	22.70	2282.2	22.6
13	55.24	5552.4	63.4	35	29.65	2980.0	526.0	57	22.67	2279.0	153.6
14	47.53	4778.0	64.8	36	29.57	2972.3	40.9	58	19.70	1980.6	17.6
15	47.21	4745.8	60.7	37	29.45	2960.4	59.6	59	18.24	1833.7	56.7
16	46.77	4701.1	62.2	38	29.38	2952.8	12.8	60	16.79	1687.4	57.3
17	41.70	4191.1	37.4	39	29.34	2949.6	162.2	61	16.75	1684.0	85.6
18	39.79	3999.7	43.9	40	29.24	2939.2	56.7	62	15.53	1560.6	66.6
19	38.23	3843.1	57.3	41	29.16	2930.9	70.0	63	14.12	1419.7	25.5
20	37.73	3792.9	45.6	42	28.94	2908.9	8.4	64	14.09	1416.6	157.2
21	37.11	3730.3	63.8	43	28.41	2855.3	9.0				
22	36.82	3701.5	42.1	44	28.37	2852.1	66.7				

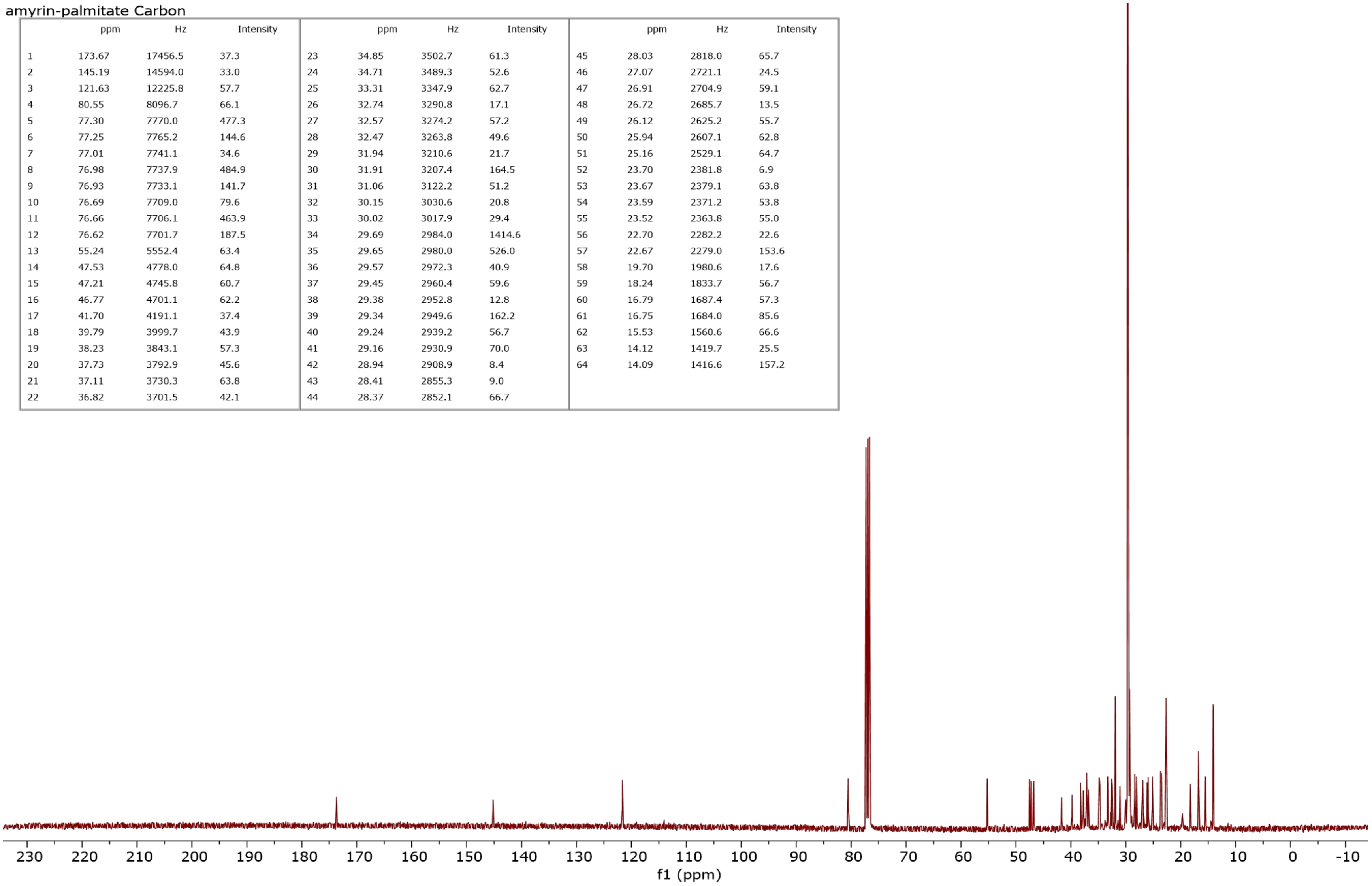

Figure S16: ^{13}C NMR spectrum of compound 3: 3β-olean-12-en-3-yl-palmitate (β-amyrin-palmitate)

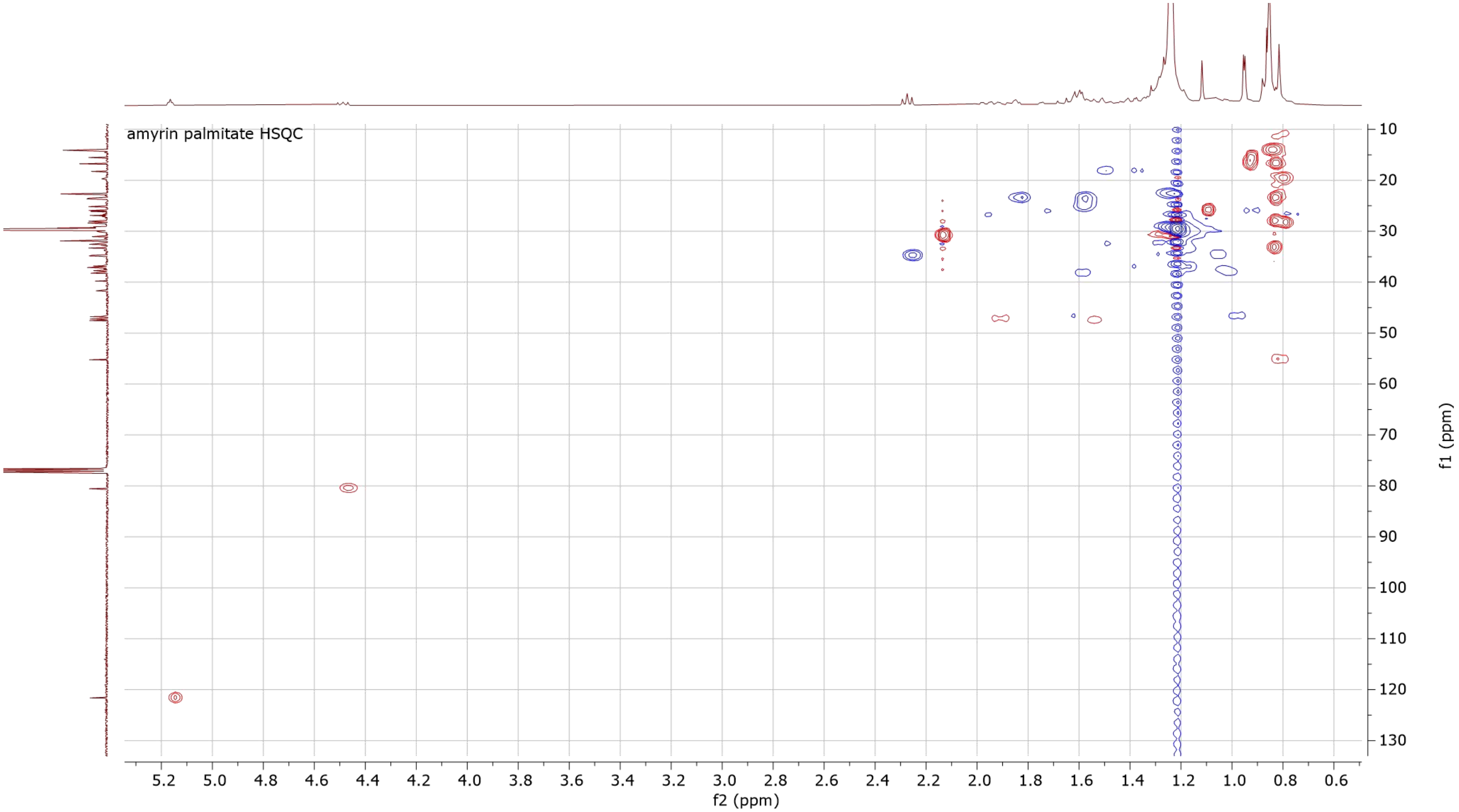

Figure S17: HSQC spectrum of compound 3: 3β-olean-12-en-3-yl-palmitate (β-amyrin-palmitate)

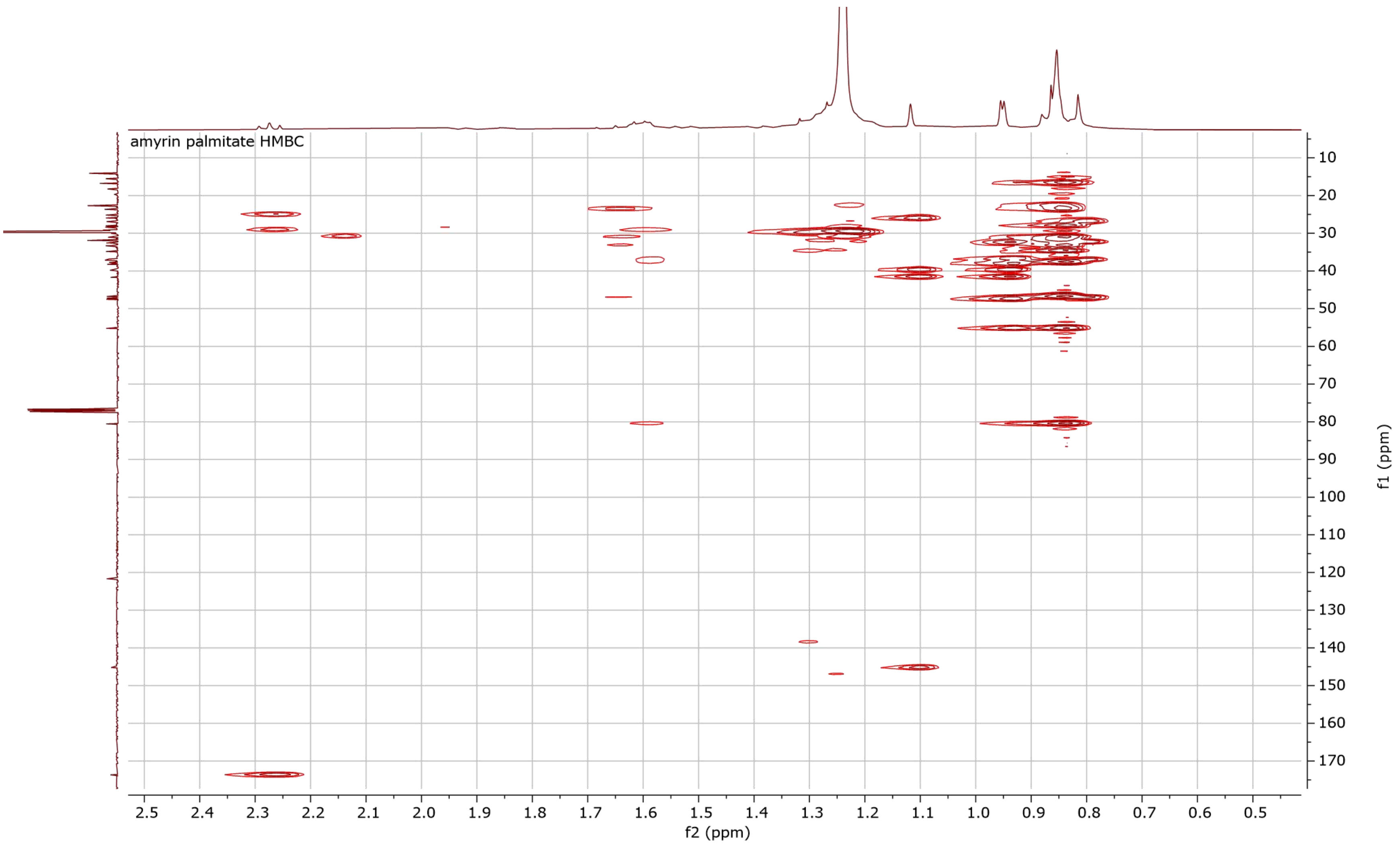

Figure S18: HMBC spectrum of compound 3: 3β-olean-12-en-3-yl-palmitate (β-amyrin-palmitate)

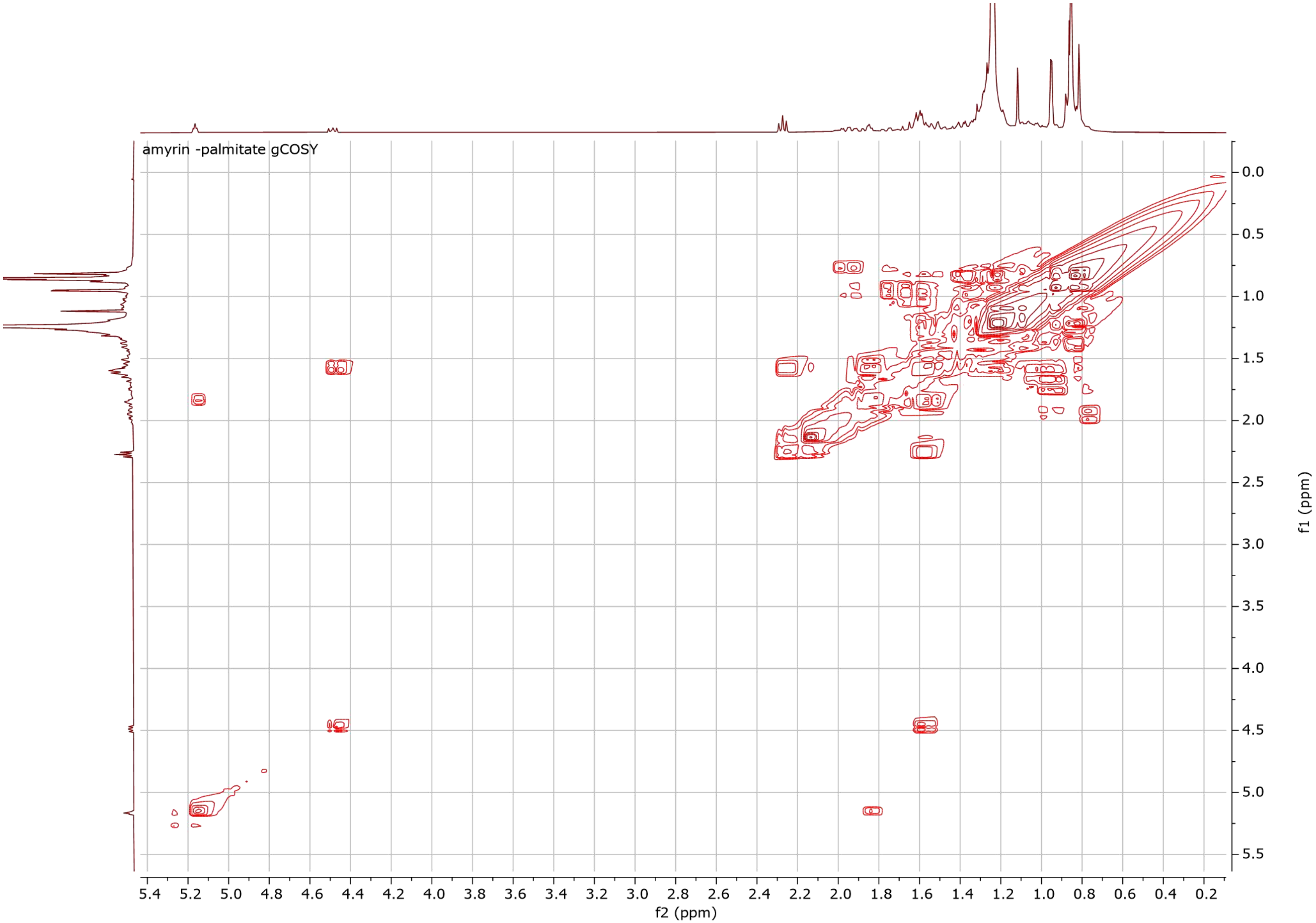

Figure S19: COSY spectrum of compound 3: 3β-olean-12-en-3-yl-palmitate (β-amyrin-palmitate)

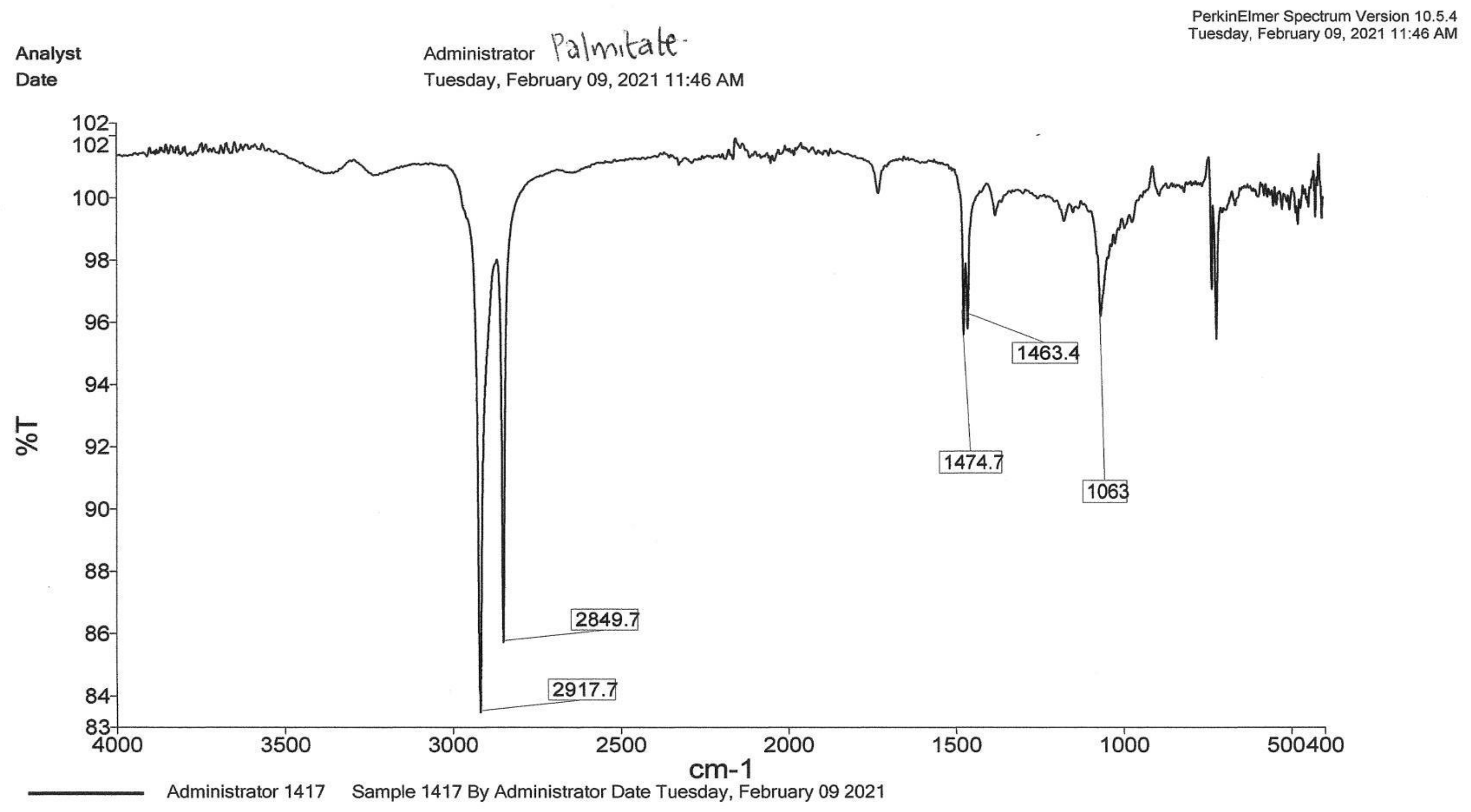

Figure S20: IR spectrum of compound 3: 3β-olean-12-en-3-yl-palmitate (β-amyrin-palmitate)

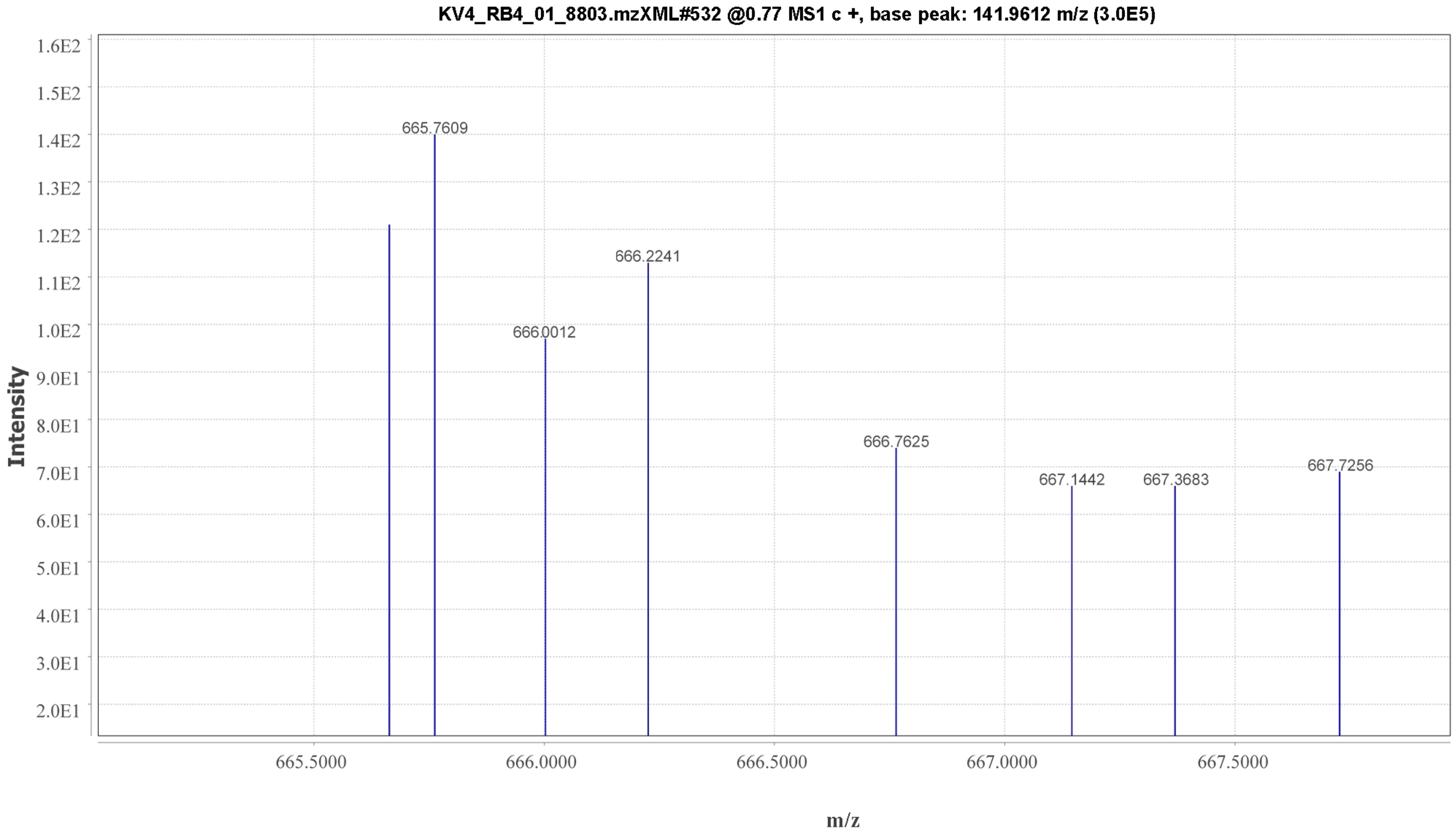

Figure S21: MS spectrum of compound 3: 3β-olean-12-en-3-yl-palmitate (β-amyrin-palmitate)

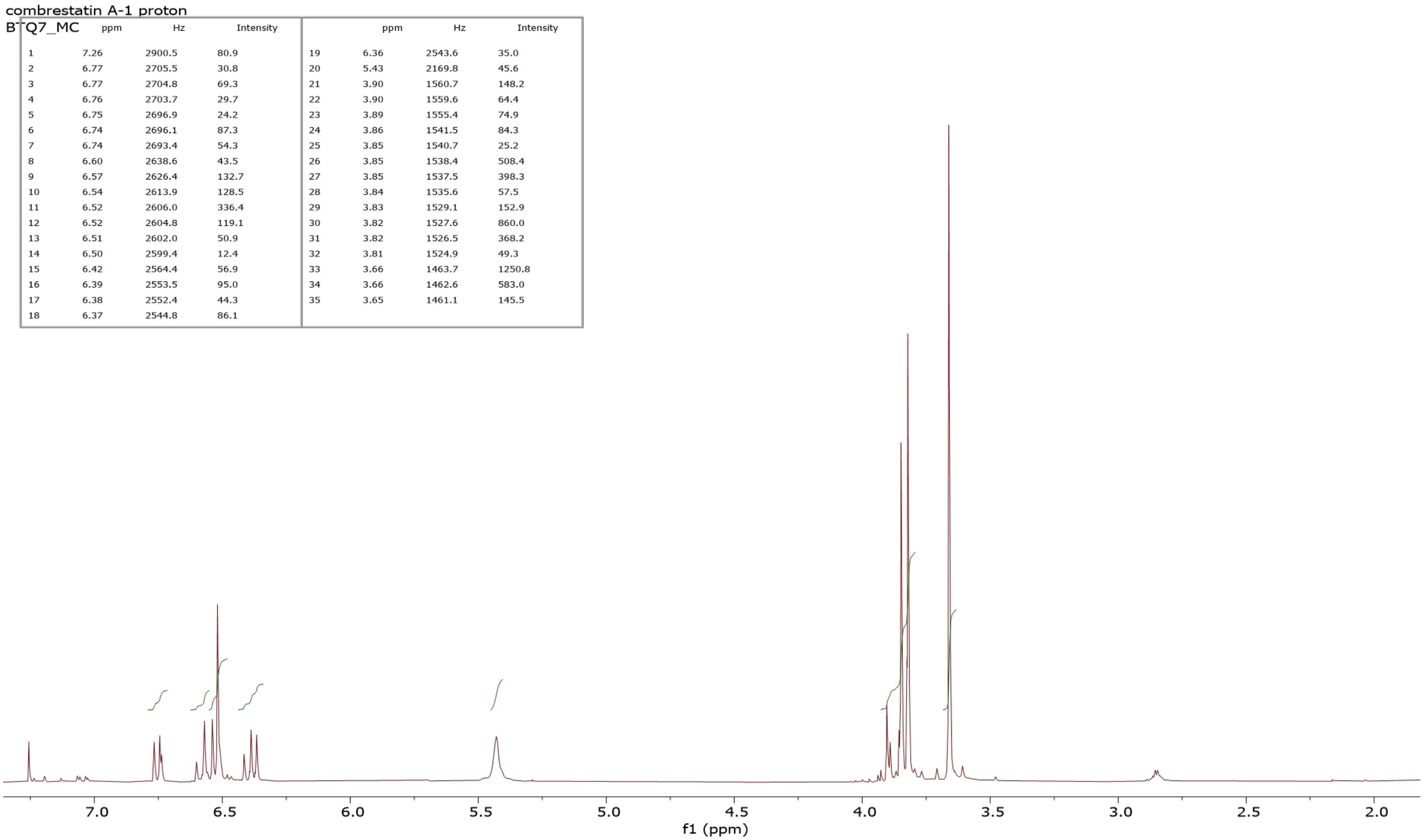

	ppm	Hz	Intensity		ppm	Hz	Intensity
1	7.26	2900.5	80.9	19	6.36	2543.6	35.0
2	6.77	2705.5	30.8	20	5.43	2169.8	45.6
3	6.77	2704.8	69.3	21	3.90	1560.7	148.2
4	6.76	2703.7	29.7	22	3.90	1559.6	64.4
5	6.75	2696.9	24.2	23	3.89	1555.4	74.9
6	6.74	2696.1	87.3	24	3.86	1541.5	84.3
7	6.74	2693.4	54.3	25	3.85	1540.7	25.2
8	6.60	2638.6	43.5	26	3.85	1538.4	508.4
9	6.57	2626.4	132.7	27	3.85	1537.5	398.3
10	6.54	2613.9	128.5	28	3.84	1535.6	57.5
11	6.52	2606.0	336.4	29	3.83	1529.1	152.9
12	6.52	2604.8	119.1	30	3.82	1527.6	860.0
13	6.51	2602.0	50.9	31	3.82	1526.5	368.2
14	6.50	2599.4	12.4	32	3.81	1524.9	49.3
15	6.42	2564.4	56.9	33	3.66	1463.7	1250.8
16	6.39	2553.5	95.0	34	3.66	1462.6	583.0
17	6.38	2552.4	44.3	35	3.65	1461.1	145.5
18	6.37	2544.8	86.1				

Figure S22: ^{1}H NMR spectrum of compound 4: 3,4,5-trimetoxy-2′,3′hydroxyl-4′methoxy-stilbene (Combrestatin A-1)

Combretastatin A-1 Carbon

BTQ7_MC

	ppm	Hz	Intensity
1	152.98	15377.0	3.2
2	152.81	15360.3	16.1
3	146.32	14708.2	8.7
4	141.63	14236.5	10.7
5	137.31	13802.1	4.2
6	132.62	13330.5	8.6
7	132.49	13317.5	10.6
8	130.27	13094.1	14.4
9	124.05	12469.3	14.1
10	120.34	12095.8	18.3
11	117.86	11847.2	9.1
12	105.98	10652.6	20.4
13	102.92	10345.6	11.3
14	77.32	7771.6	60.8
15	77.00	7739.7	60.3
16	76.68	7707.8	58.8
17	60.87	6118.0	5.4
18	60.84	6115.2	6.3
19	56.19	5648.3	6.5
20	56.16	5645.4	8.3
21	55.86	5614.6	13.2
22	55.83	5611.7	16.5

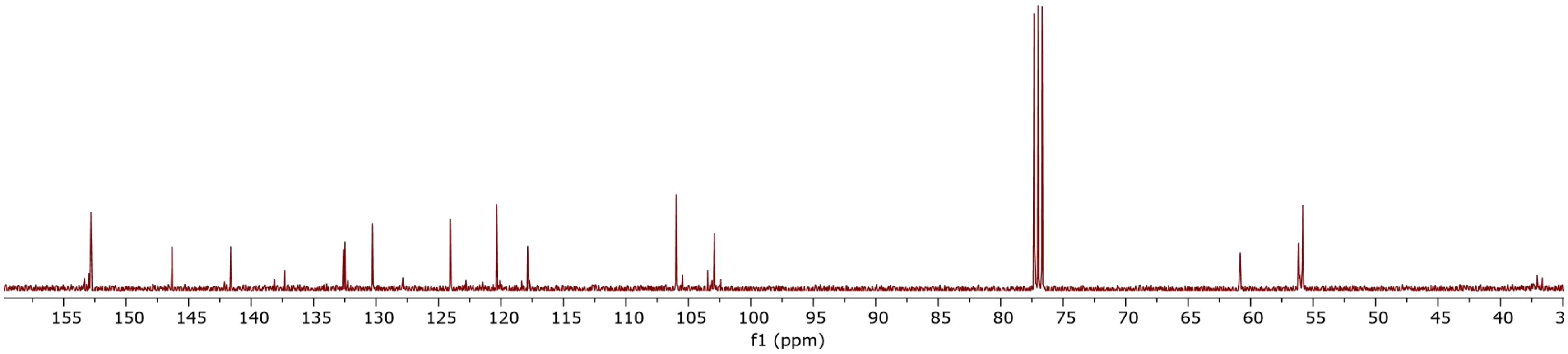

Figure S23: ^{13}C NMR spectrum of compound 4: 3,4,5-trimetoxy-2′,3′hydroxyl-4′methoxy-stilbene (Combrestatin A-1)

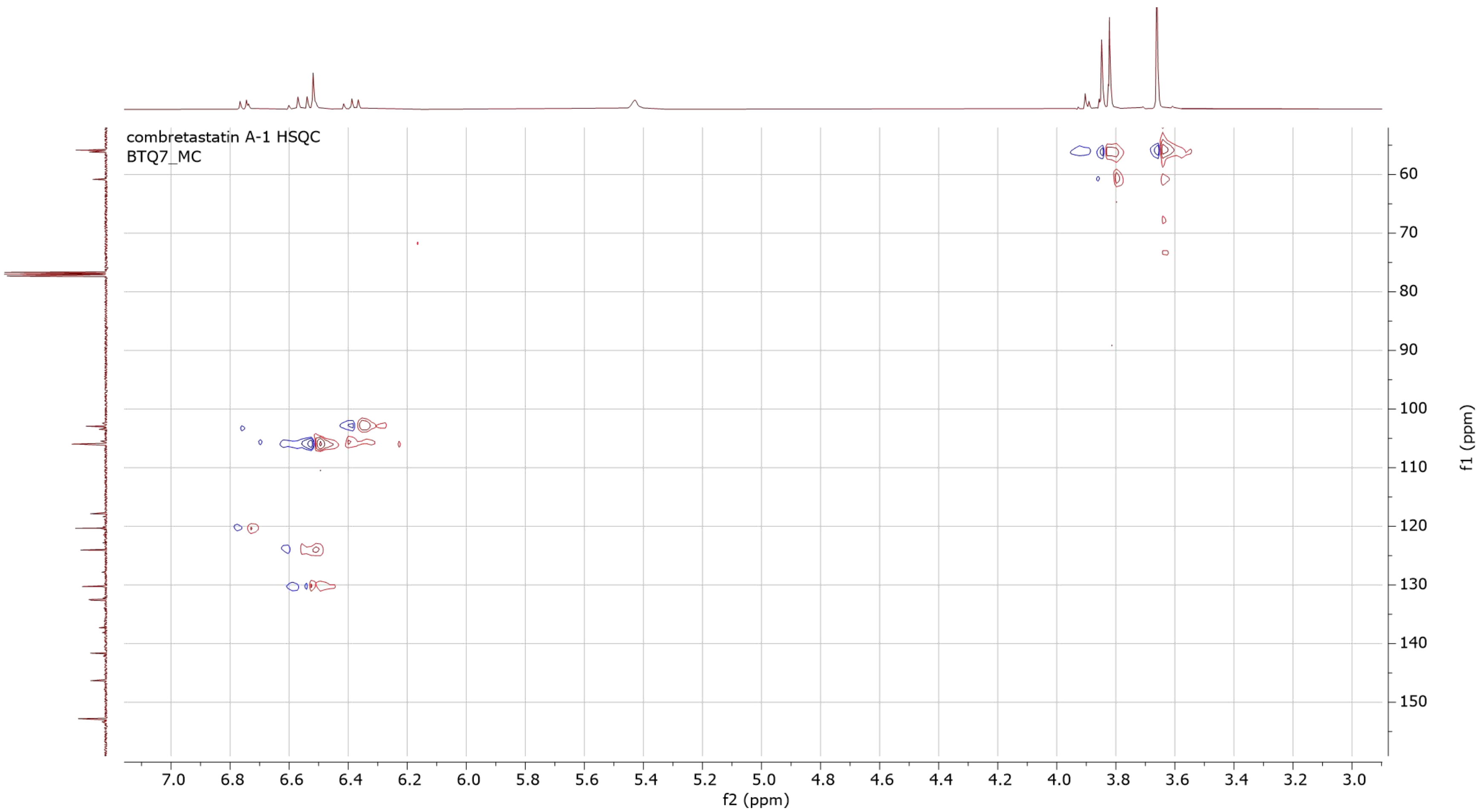

Figure S24: HSQC spectrum of compound 4: 3,4,5-trimetoxy-2’,3’hydroxyl-4’methoxy-stilbene (Combrestatin A-1)

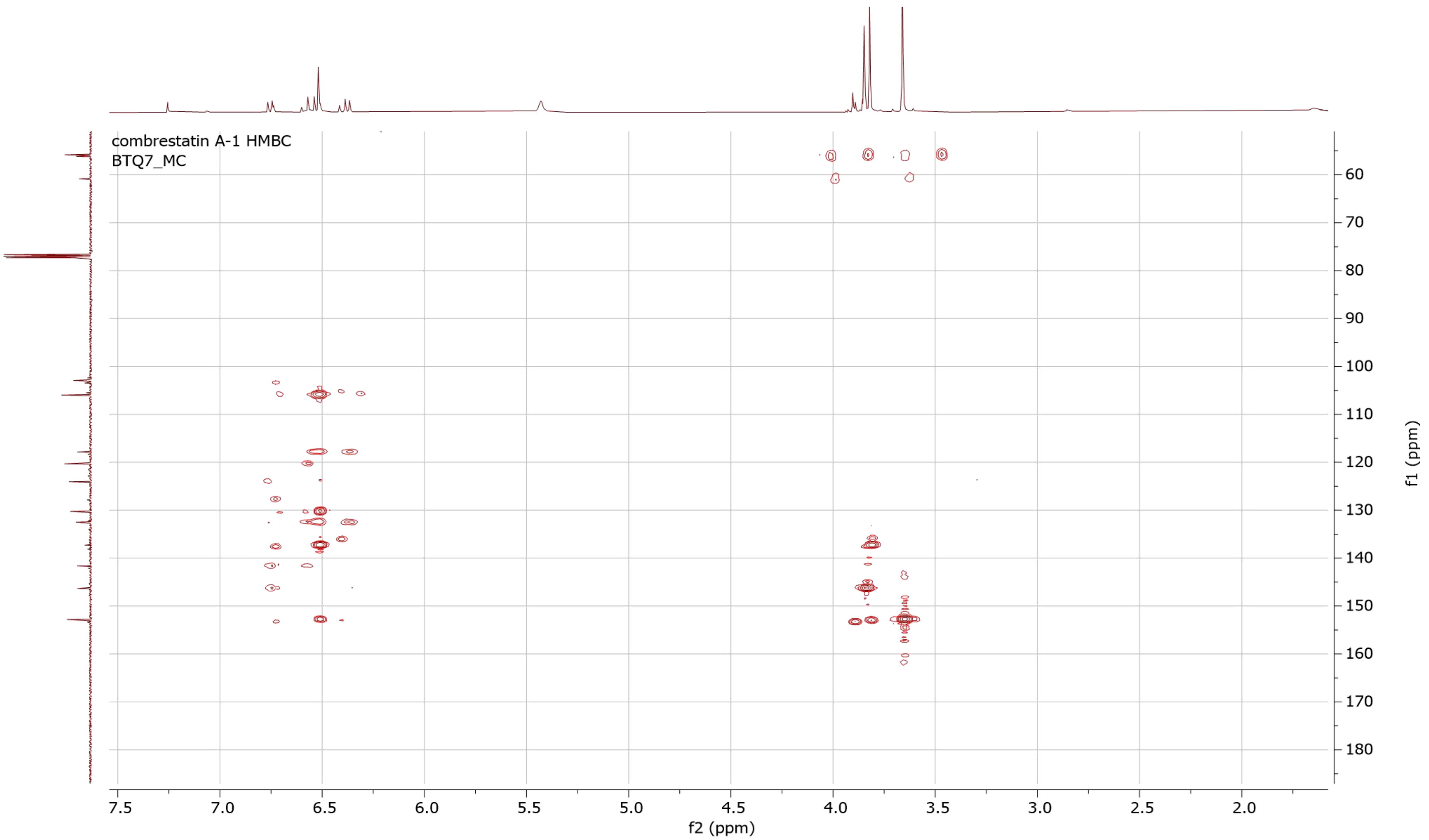

Figure S25: HMBC spectrum of compound **4**: 3,4,5-trimetoxy-2′,3′hydroxyl-4′methoxy-stilbene (Combrestatin A-1)

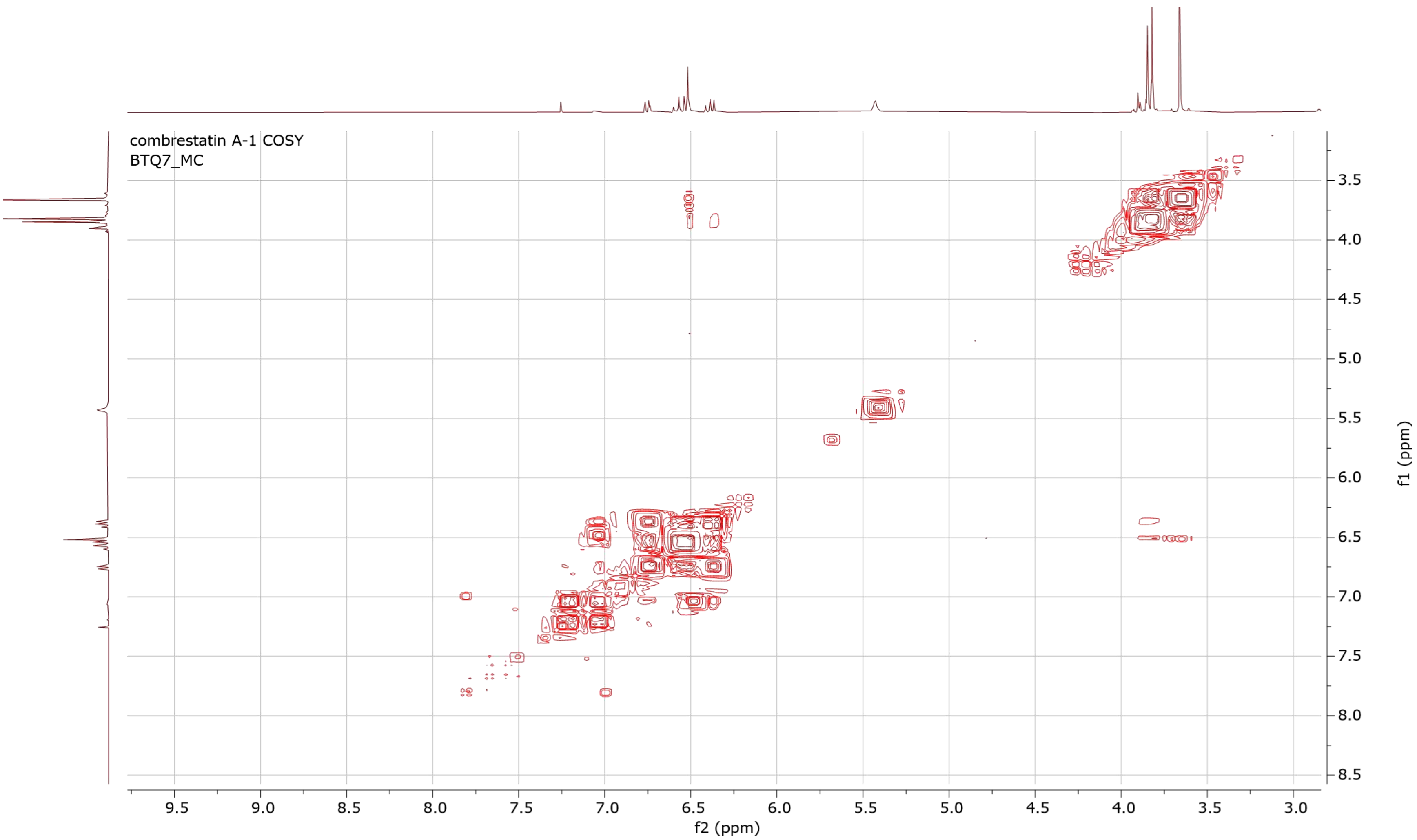

Figure S26: COSY spectrum of compound **4**: 3,4,5-trimetoxy-2',3'hydroxyl-4'methoxy-stilbene (Combrestatin A-1)

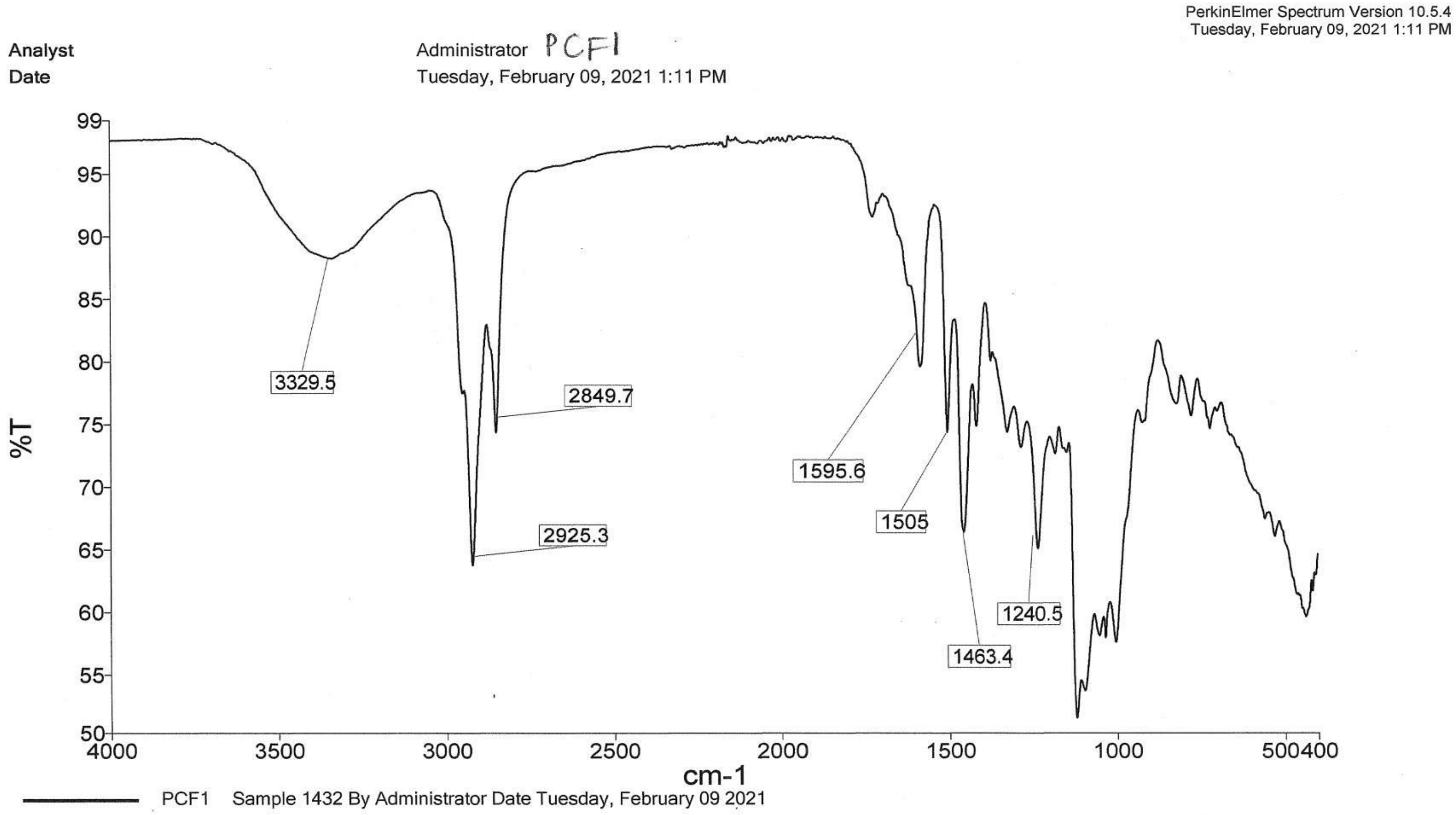

Figure S27: IR spectrum of compound **4**: 3,4,5-trimetoxy-2′,3′hydroxyl-4′methoxy-stilbene (Combrestatin A-1)

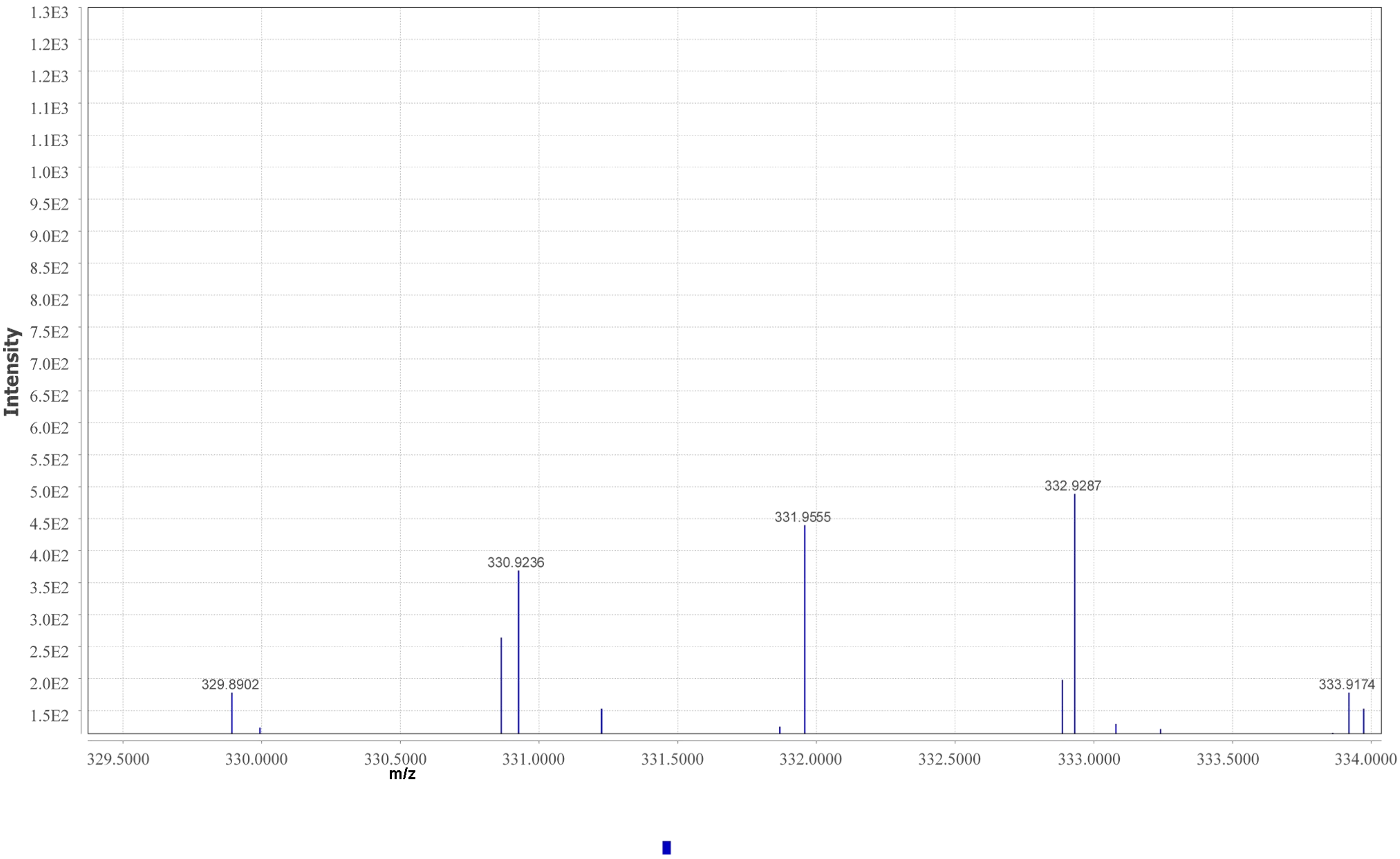

Figure S28: MS spectrum of compound **4**: 3,4,5-trimetoxy-2’,3’hydroxyl-4’methoxy-stilbene (Combrestatin A-1)

Combretastatin Mixture Proton

	ppm	Hz	Intensity		ppm	Hz	Intensity		ppm	Hz	Intensity
1	8.10	3236.4	50.2	31	4.42	1768.2	33.7	61	3.37	1349.1	4.0
2	7.94	3173.6	43.8	32	4.14	1655.8	8.7	62	3.21	1284.9	123.5
3	7.49	2994.5	5.7	33	4.13	1652.9	137.0	63	2.95	1177.9	18.4
4	7.25	2899.6	169.5	34	4.12	1645.8	325.3	64	2.93	1170.7	17.9
5	6.98	2789.5	5.9	35	4.11	1643.9	22.1	65	2.91	1164.5	16.2
6	6.96	2781.0	3.8	36	4.10	1638.7	278.3	66	2.82	1125.3	19.4
7	6.70	2676.7	13.9	37	4.08	1631.6	84.6	67	2.80	1118.3	11.8
8	6.66	2661.0	103.2	38	3.94	1575.6	7.1	68	2.76	1104.9	4.0
9	6.64	2653.8	117.6	39	3.92	1568.1	5.9	69	2.71	1084.8	81.7
10	6.59	2632.9	3.1	40	3.91	1561.6	8.0	70	2.70	1077.7	66.8
11	6.57	2626.5	4.4	41	3.89	1555.5	2.7	71	2.20	880.5	3.8
12	6.55	2617.8	3.5	42	3.85	1537.9	7.7	72	2.19	875.3	7.1
13	6.52	2607.1	8.1	43	3.83	1531.7	51.2	73	2.15	861.2	4.7
14	6.49	2592.7	93.3	44	3.82	1528.1	54.5	74	2.09	835.5	5.9
15	6.46	2582.7	85.4	45	3.81	1524.9	67.2	75	2.03	811.5	961.8
16	6.44	2574.4	101.4	46	3.81	1522.9	9.5	76	1.87	745.9	6.2
17	6.37	2548.3	168.2	47	3.78	1511.4	14.1	77	1.40	560.2	5.0
18	6.36	2542.8	371.6	48	3.76	1503.8	308.3	78	1.32	528.9	4.2
19	6.31	2523.8	156.7	49	3.75	1497.9	735.2	79	1.28	511.8	26.4
20	6.30	2517.9	339.0	50	3.74	1494.7	206.4	80	1.28	510.3	108.5
21	5.93	2370.5	3.9	51	3.73	1491.0	438.3	81	1.26	504.7	446.7
22	5.59	2235.9	32.9	52	3.73	1489.1	825.3	82	1.25	501.0	177.0
23	5.49	2192.9	61.1	53	3.71	1484.5	989.7	83	1.25	497.7	652.7
24	5.30	2118.6	3.3	54	3.70	1480.0	432.8	84	1.23	490.5	270.9
25	5.28	2112.3	8.9	55	3.69	1474.5	766.0	85	0.89	354.0	16.4
26	5.11	2042.6	43.7	56	3.64	1457.1	5.5	86	0.87	347.4	28.6
27	4.67	1867.5	71.9	57	3.57	1427.7	477.2	87	0.85	340.5	3.3
28	4.65	1860.0	35.6	58	3.56	1422.0	946.1	88	0.84	336.5	30.4
29	4.56	1821.9	5.3	59	3.46	1382.5	3.2	89	0.83	333.5	3.8
30	4.44	1775.6	73.3	60	3.44	1377.0	10.7				

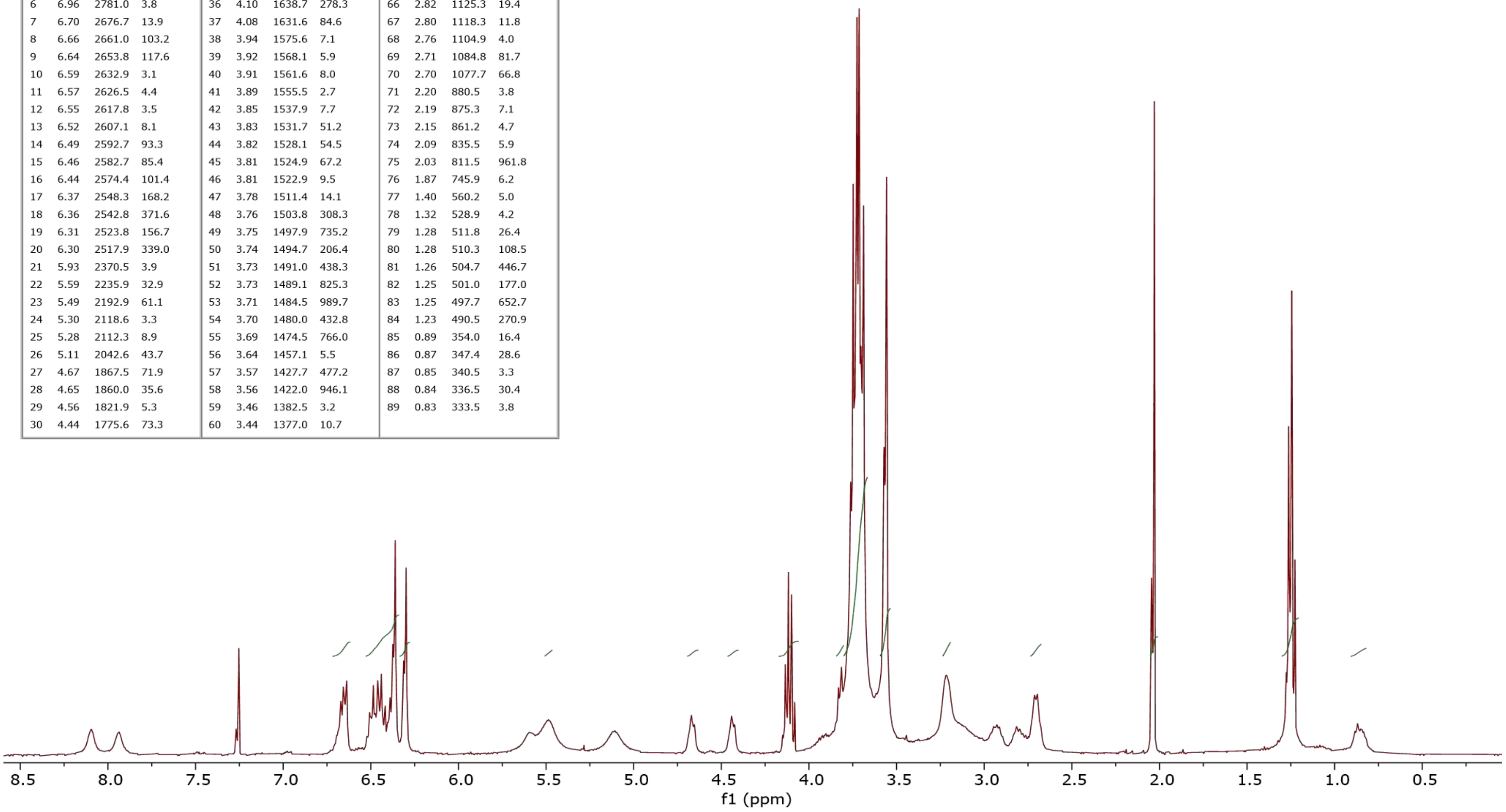

Figure S29: ^{1}H NMR spectrum of compound **5a** and **5b**: combretastatin A-1-2′*O*-β-*D*-glucopyranoside and combretastatin B-1-2′*O*-β-*D*-glucopyranoside

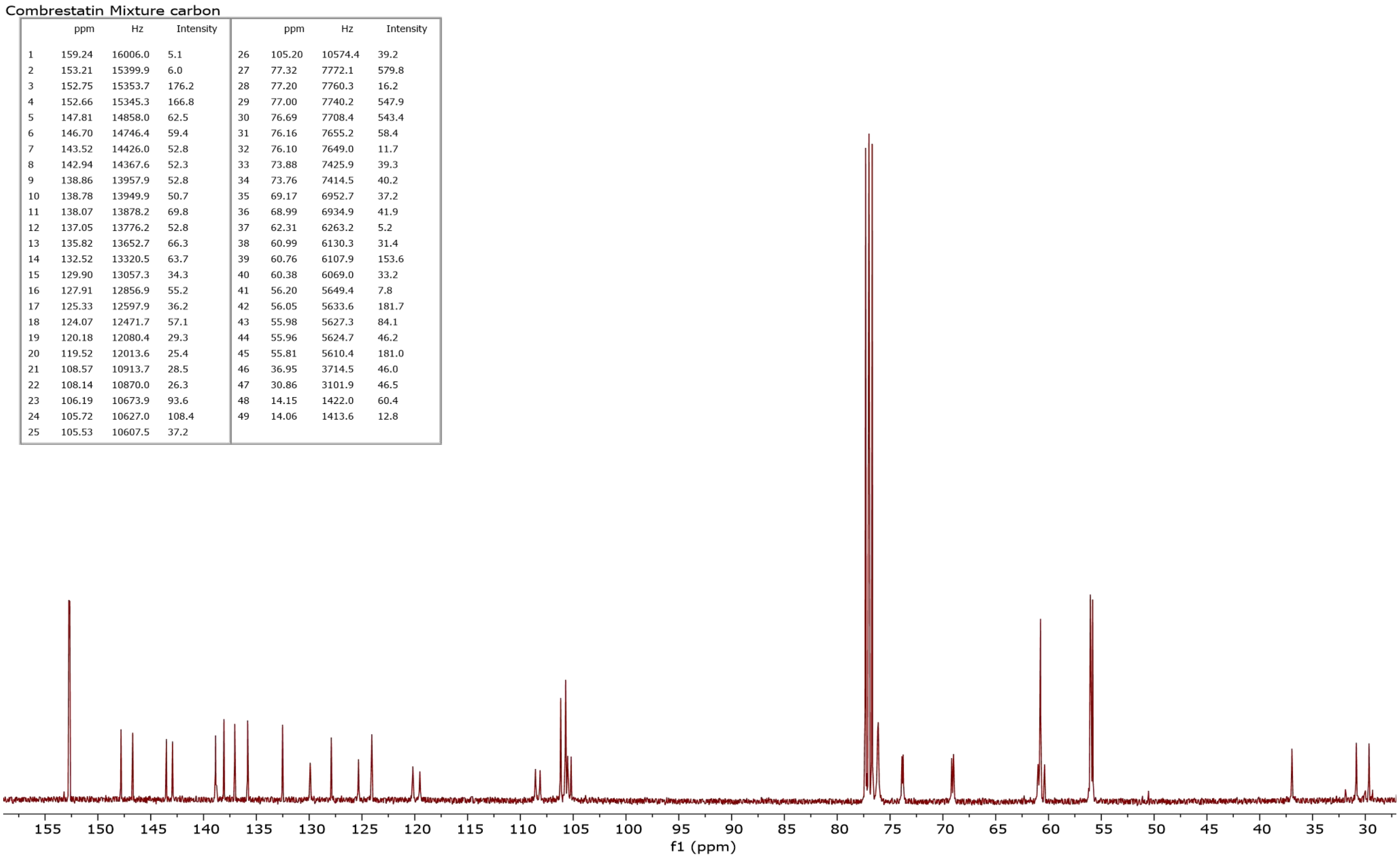

	ppm	Hz	Intensity		ppm	Hz	Intensity
1	159.24	16006.0	5.1	26	105.20	10574.4	39.2
2	153.21	15399.9	6.0	27	77.32	7772.1	579.8
3	152.75	15353.7	176.2	28	77.20	7760.3	16.2
4	152.66	15345.3	166.8	29	77.00	7740.2	547.9
5	147.81	14858.0	62.5	30	76.69	7708.4	543.4
6	146.70	14746.4	59.4	31	76.16	7655.2	58.4
7	143.52	14426.0	52.8	32	76.10	7649.0	11.7
8	142.94	14367.6	52.3	33	73.88	7425.9	39.3
9	138.86	13957.9	52.8	34	73.76	7414.5	40.2
10	138.78	13949.9	50.7	35	69.17	6952.7	37.2
11	138.07	13878.2	69.8	36	68.99	6934.9	41.9
12	137.05	13776.2	52.8	37	62.31	6263.2	5.2
13	135.82	13652.7	66.3	38	60.99	6130.3	31.4
14	132.52	13320.5	63.7	39	60.76	6107.9	153.6
15	129.90	13057.3	34.3	40	60.38	6069.0	33.2
16	127.91	12856.9	55.2	41	56.20	5649.4	7.8
17	125.33	12597.9	36.2	42	56.05	5633.6	181.7
18	124.07	12471.7	57.1	43	55.98	5627.3	84.1
19	120.18	12080.4	29.3	44	55.96	5624.7	46.2
20	119.52	12013.6	25.4	45	55.81	5610.4	181.0
21	108.57	10913.7	28.5	46	36.95	3714.5	46.0
22	108.14	10870.0	26.3	47	30.86	3101.9	46.5
23	106.19	10673.9	93.6	48	14.15	1422.0	60.4
24	105.72	10627.0	108.4	49	14.06	1413.6	12.8
25	105.53	10607.5	37.2				

Figure S30: ^{13}C NMR spectrum of compound **5a** and **5b**: combretastatin A-1-2′*O*-β-*D*-glucopyranoside and combretastatin B-1-2′*O*-β-*D*-glucopyranoside

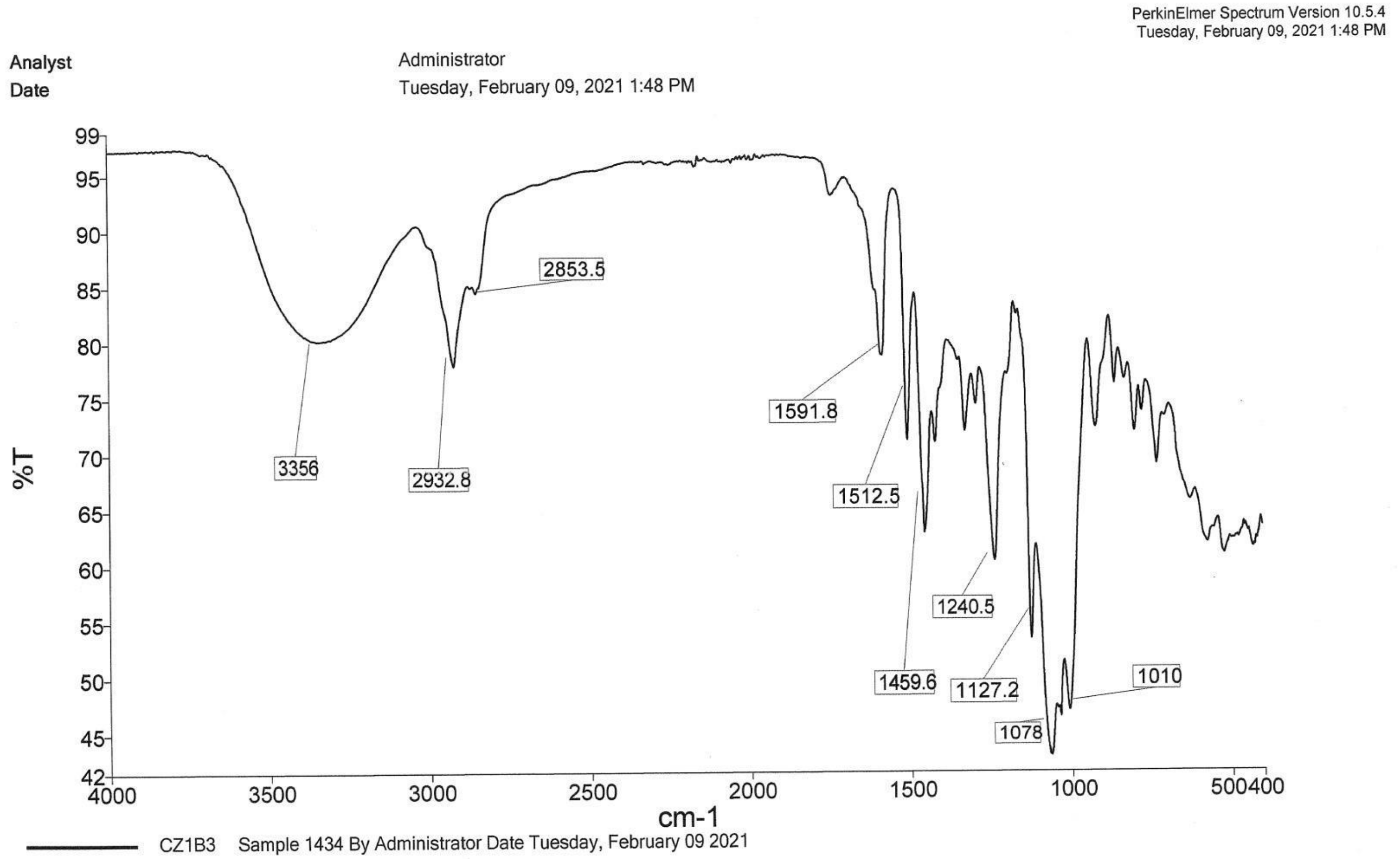

Figure S31: IR spectrum of compound **5a** and **5b**: combretastatin A-1-2′*O*-β-*D*-glucopyranoside and combretastatin B-1-2′*O*-β-*D*-glucopyranoside

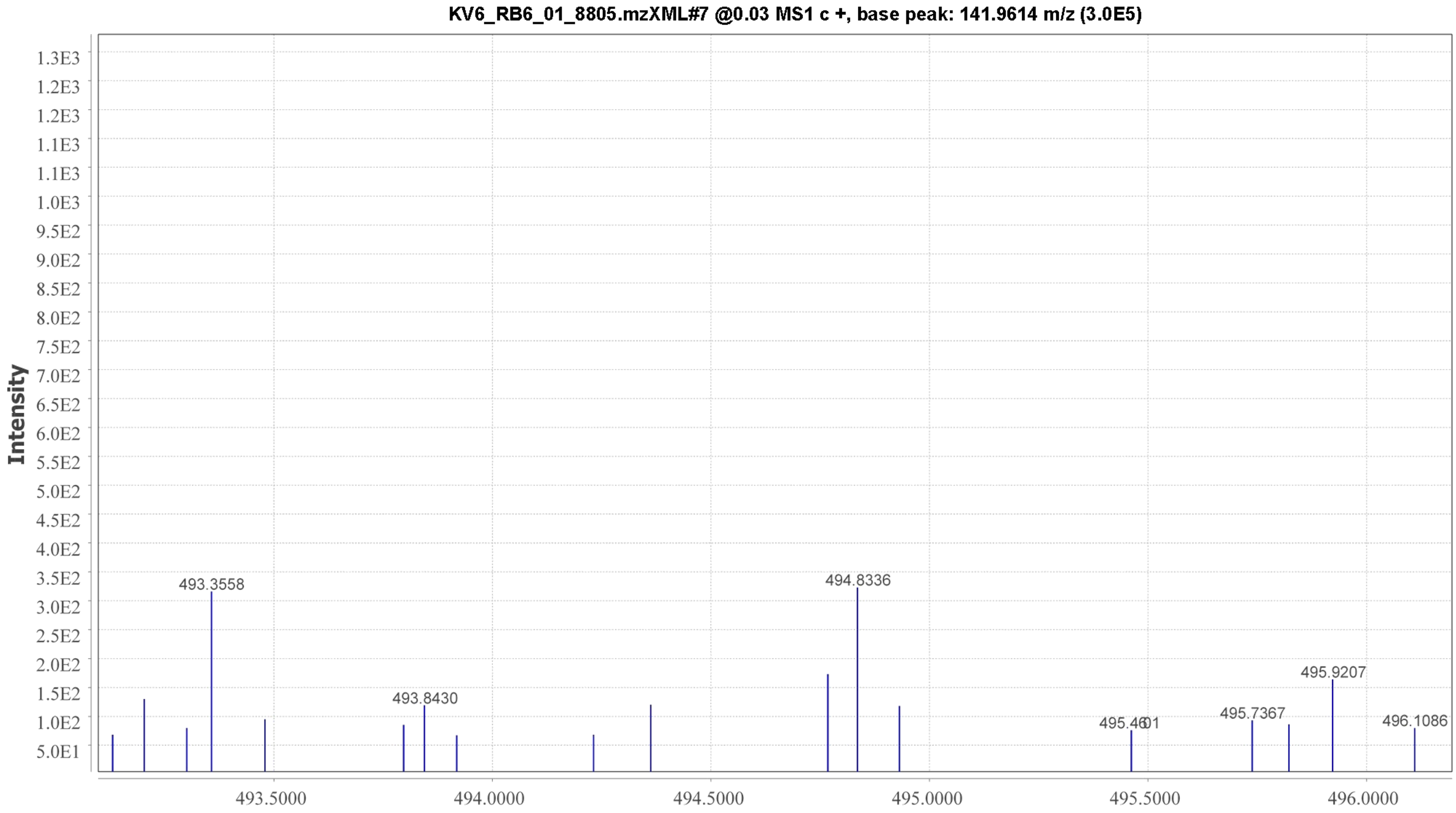

Figure S32: MS spectrum of compound **5a**: combretastatin A-1-2′*O*-β-*D*-glucopyranoside and combretastatin B-1-2′*O*-β-*D*glucopyranoside

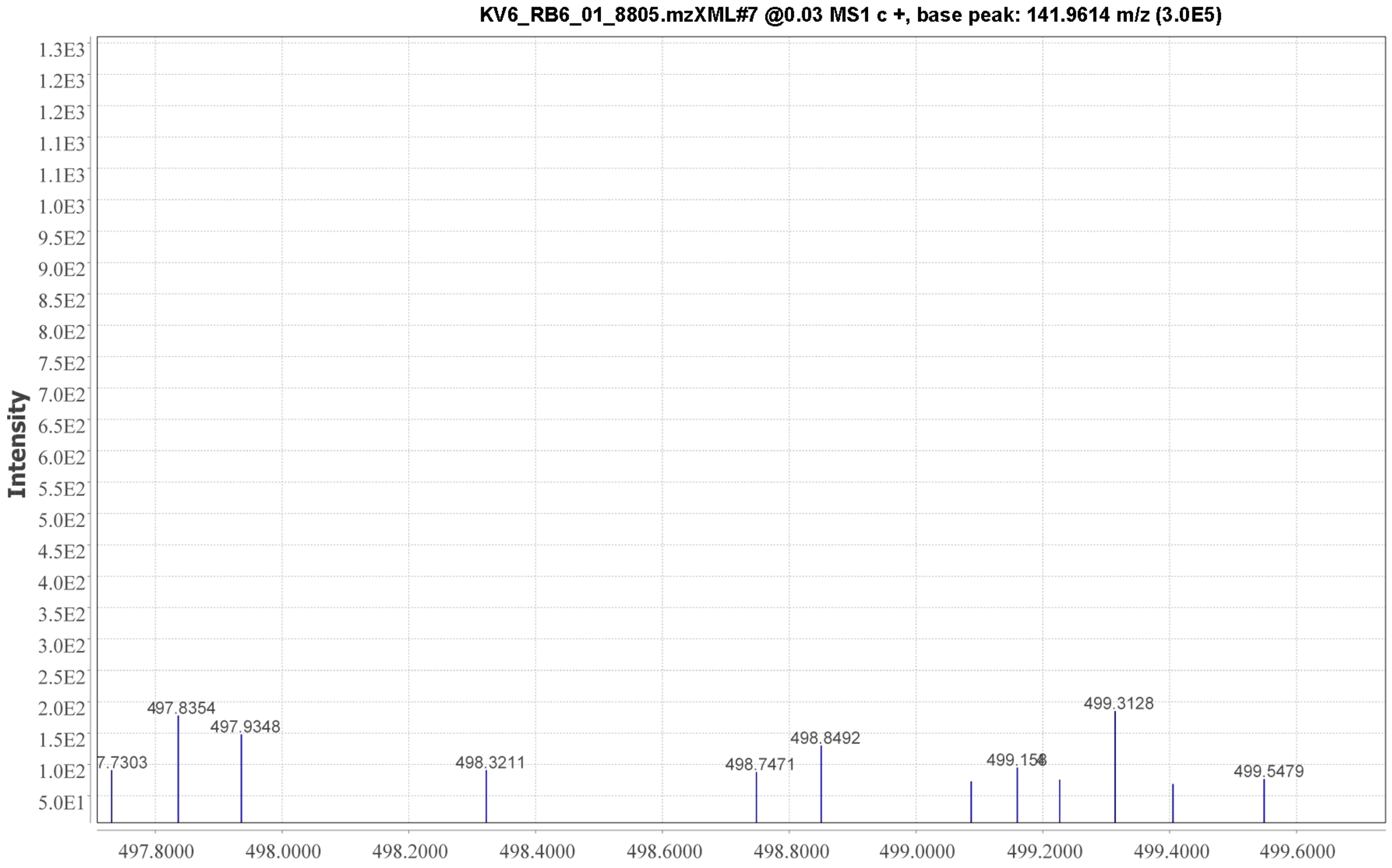

Figure S33: MS spectrum of compound **5b**: combretastatin A-1-2′*O*-β-*D*-glucopyranoside and combretastatin B-1-2′*O*-β-*D*glucopyranoside

Stigmasterol proton

	ppm	Hz	Intensity
1	7.24	2895.3	51.7
2	5.28	2109.3	13.8
3	5.17	2068.1	13.9
4	5.16	2064.4	17.3
5	5.16	2060.8	7.2
6	5.12	2046.0	14.6
7	5.11	2042.4	17.9
8	5.10	2038.7	7.9
9	4.67	1866.1	5.9
10	4.55	1819.1	4.7
11	4.55	1817.5	2.8
12	4.54	1816.4	3.3
13	3.22	1289.0	16.1
14	3.22	1287.5	2.2
15	3.21	1283.8	14.1
16	3.21	1282.8	2.6
17	3.20	1278.2	17.2
18	3.19	1276.7	6.1
19	3.18	1273.2	13.5
20	3.18	1271.5	2.7
21	2.20	878.9	20.9
22	2.20	878.4	76.9
23	2.17	866.0	2.2
24	2.16	864.7	7.9
25	2.16	864.6	31.4
26	2.16	862.7	28.5
27	2.15	860.0	99.8
28	2.15	858.3	818.9
29	2.10	838.4	62.0
30	1.91	764.3	18.1
31	1.91	762.4	12.2
32	1.90	758.6	3.9
33	1.89	755.2	20.8
34	1.88	751.6	19.3
35	1.86	741.6	18.2
36	1.85	737.8	16.5
37	1.68	670.9	8.2
38	1.67	666.5	10.0
39	1.66	663.7	30.8
40	1.64	657.4	13.5
41	1.62	649.4	13.5
42	1.62	646.5	16.6
43	1.62	646.4	8.6
44	1.61	643.4	16.1
45	1.59	637.1	30.5
46	1.58	633.1	14.7
47	1.58	630.5	7.3
48	1.57	629.2	2.9
49	1.57	628.0	4.1
50	1.56	622.5	12.1
51	1.55	619.6	2.2
52	1.54	615.7	17.8
53	1.53	611.3	16.3
54	1.53	609.7	6.2
55	1.52	606.5	5.6
56	1.51	605.0	11.2
57	1.51	602.3	10.3
58	1.50	599.8	7.6
59	1.48	592.0	3.8
60	1.46	584.3	1.8
61	1.45	578.3	2.1
62	1.43	572.3	5.4
63	1.42	568.2	4.8
64	1.41	564.9	10.6
65	1.40	560.8	15.0
66	1.39	557.2	19.5
67	1.39	555.8	2.9
68	1.38	551.0	7.7
69	1.37	548.1	18.9
70	1.37	546.0	3.5
71	1.36	542.6	6.8
72	1.35	537.9	15.0
73	1.33	531.6	3.4
74	1.32	529.5	2.0
75	1.32	526.1	4.1
76	1.31	525.2	8.2
77	1.30	521.4	8.8
78	1.30	519.1	29.1
79	1.27	508.4	19.9
80	1.26	505.2	8.5
81	1.24	496.3	86.0
82	1.24	494.5	54.4
83	1.23	493.6	44.1
84	1.22	488.0	9.3
85	1.21	484.6	12.3
86	1.19	474.3	14.2
87	1.18	471.7	3.9
88	1.17	468.8	1.6
89	1.17	466.0	7.1
90	1.12	446.4	110.8
91	1.11	445.6	27.4
92	1.10	440.7	6.2
93	1.09	435.0	3.1
94	1.06	424.7	26.7
95	1.05	421.1	107.5
96	1.05	420.2	35.8
97	1.04	416.1	6.5
98	1.03	411.1	32.1
99	1.02	407.1	12.9
100	1.01	404.5	49.8
101	1.00	399.9	51.2
102	0.99	396.5	147.8
103	0.98	393.0	63.8
104	0.98	391.2	239.0
105	0.97	386.8	19.5
106	0.95	379.9	179.0
107	0.94	374.4	111.4
108	0.93	373.5	33.7
109	0.93	371.0	24.1
110	0.93	370.1	16.4
111	0.92	369.1	22.7
112	0.92	367.6	110.1
113	0.91	362.9	3.7
114	0.91	361.9	5.8
115	0.90	360.1	34.8
116	0.89	357.1	65.7
117	0.88	351.5	2.9
118	0.87	347.6	21.4
119	0.86	345.1	34.3
120	0.86	342.7	40.9
121	0.85	340.4	239.0
122	0.83	332.6	16.1
123	0.82	329.2	44.4
124	0.81	325.1	166.8
125	0.80	320.4	26.7
126	0.80	318.6	13.4
127	0.79	316.1	29.1
128	0.78	312.5	229.6
129	0.77	309.6	209.5
130	0.77	308.7	90.2
131	0.76	305.7	31.1
132	0.74	296.3	34.2
133	0.73	292.7	27.9
134	0.72	289.5	13.8

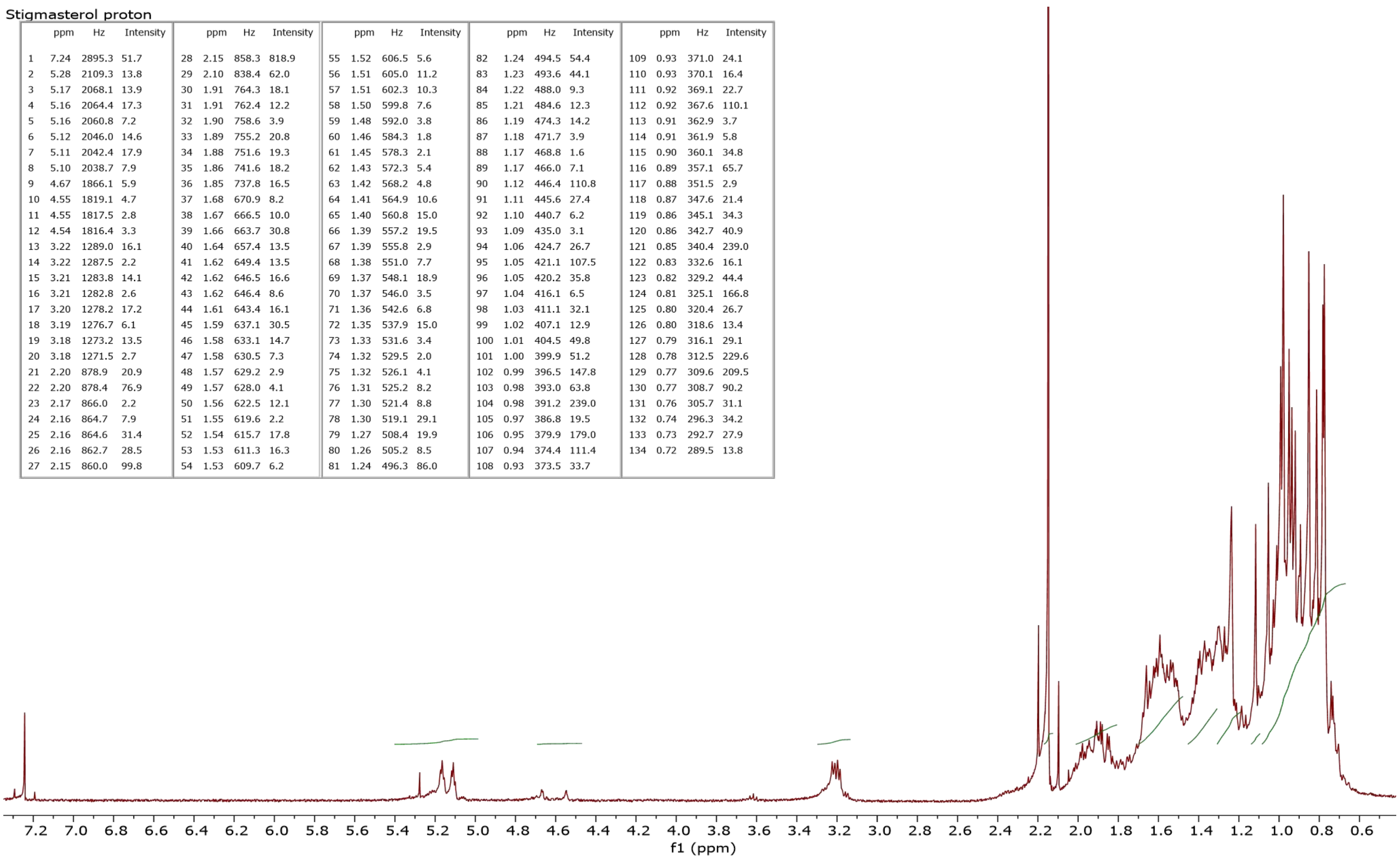

Figure S34: ^{1}H NMR spectrum of compound **6**: stigmasta-5,22-dien-3β-ol (Stigmasterol)

CARBON 03

	ppm	Hz	Intensity		ppm	Hz	Intensity
1	140.74	14147.1	48.9	20	37.21	3740.5	91.2
2	138.31	13902.7	60.2	21	36.47	3666.1	60.5
3	129.20	12987.1	57.4	22	31.87	3203.3	44.6
4	121.64	12227.5	48.0	23	31.85	3201.6	180.5
5	71.72	7209.6	87.4	24	31.61	3177.8	22.4
6	56.83	5712.7	12.9	25	31.60	3176.2	89.2
7	56.82	5711.2	65.9	26	30.93	3109.5	54.3
8	55.88	5617.1	60.7	27	30.92	3108.0	196.1
9	53.42	5369.4	32.1	28	28.91	2905.8	53.0
10	51.22	5148.1	18.7	29	25.39	2551.9	61.0
11	51.20	5146.6	68.9	30	24.33	2445.4	53.2
12	50.11	5036.7	16.5	31	21.18	2129.5	56.3
13	50.09	5035.2	72.1	32	21.08	2118.5	65.8
14	50.07	5032.6	29.0	33	21.03	2113.6	72.2
15	42.27	4248.6	29.6	34	19.38	1948.3	23.8
16	42.25	4246.7	90.0	35	19.37	1946.8	110.3
17	42.16	4238.0	50.2	36	18.94	1903.7	65.5
18	40.49	4070.5	58.9	37	12.23	1229.7	62.6
19	39.63	3983.5	57.5	38	12.01	1207.1	64.3

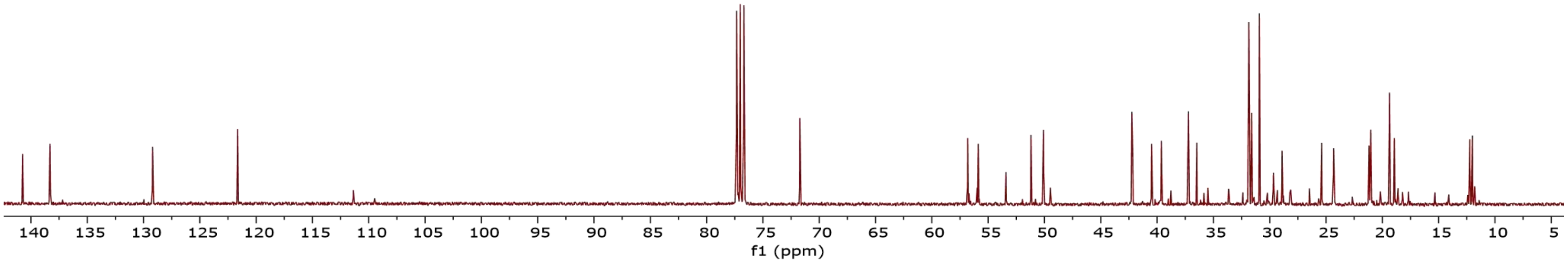

Figure S35: ^{13}C NMR spectrum of compound **6**: stigmasta-5,22-dien-3β-ol (Stigmasterol)

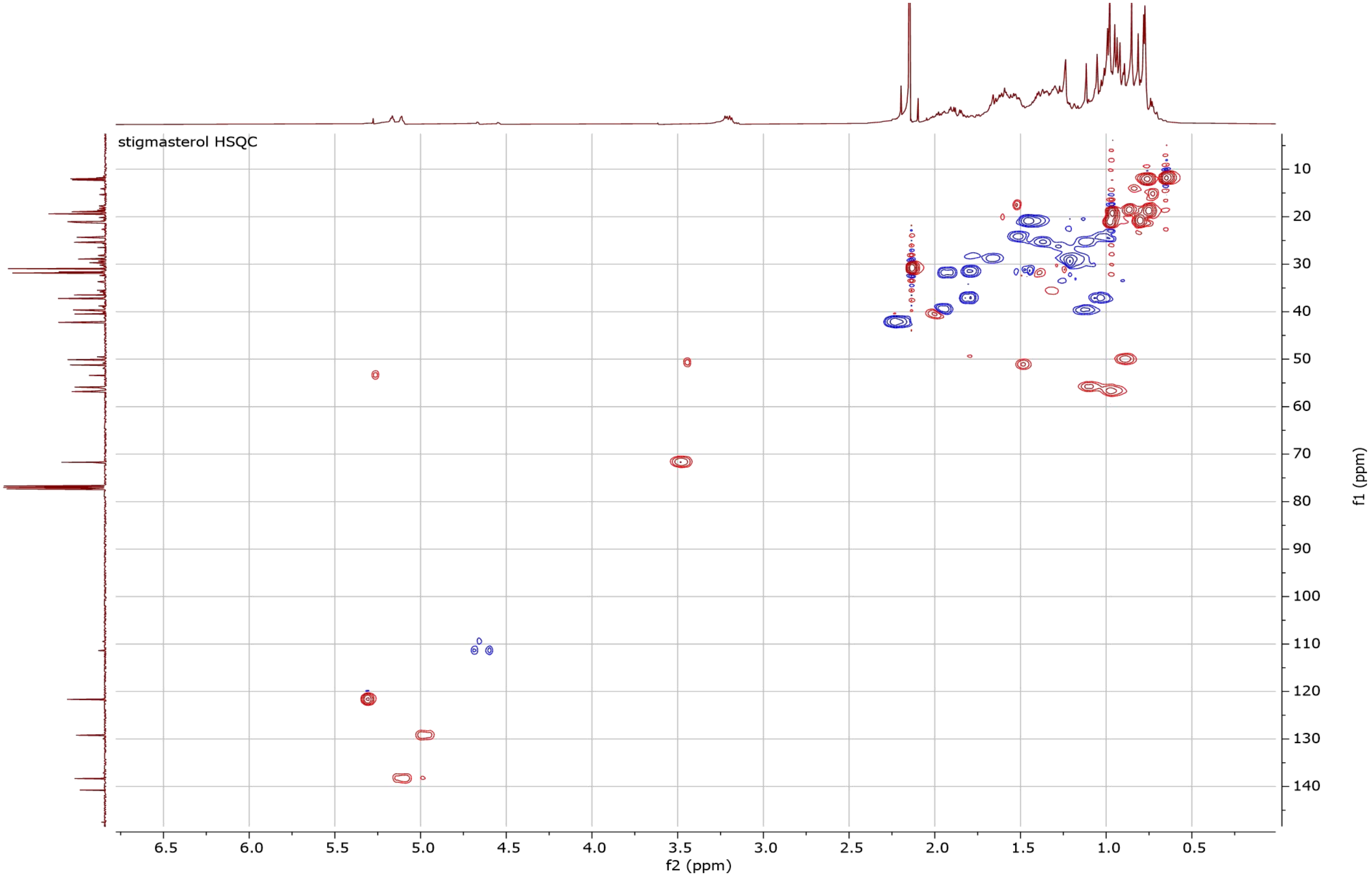

Figure S36: HSQC spectrum of compound **6**: stigmasta-5,22-dien-3β-ol (Stigmasterol)

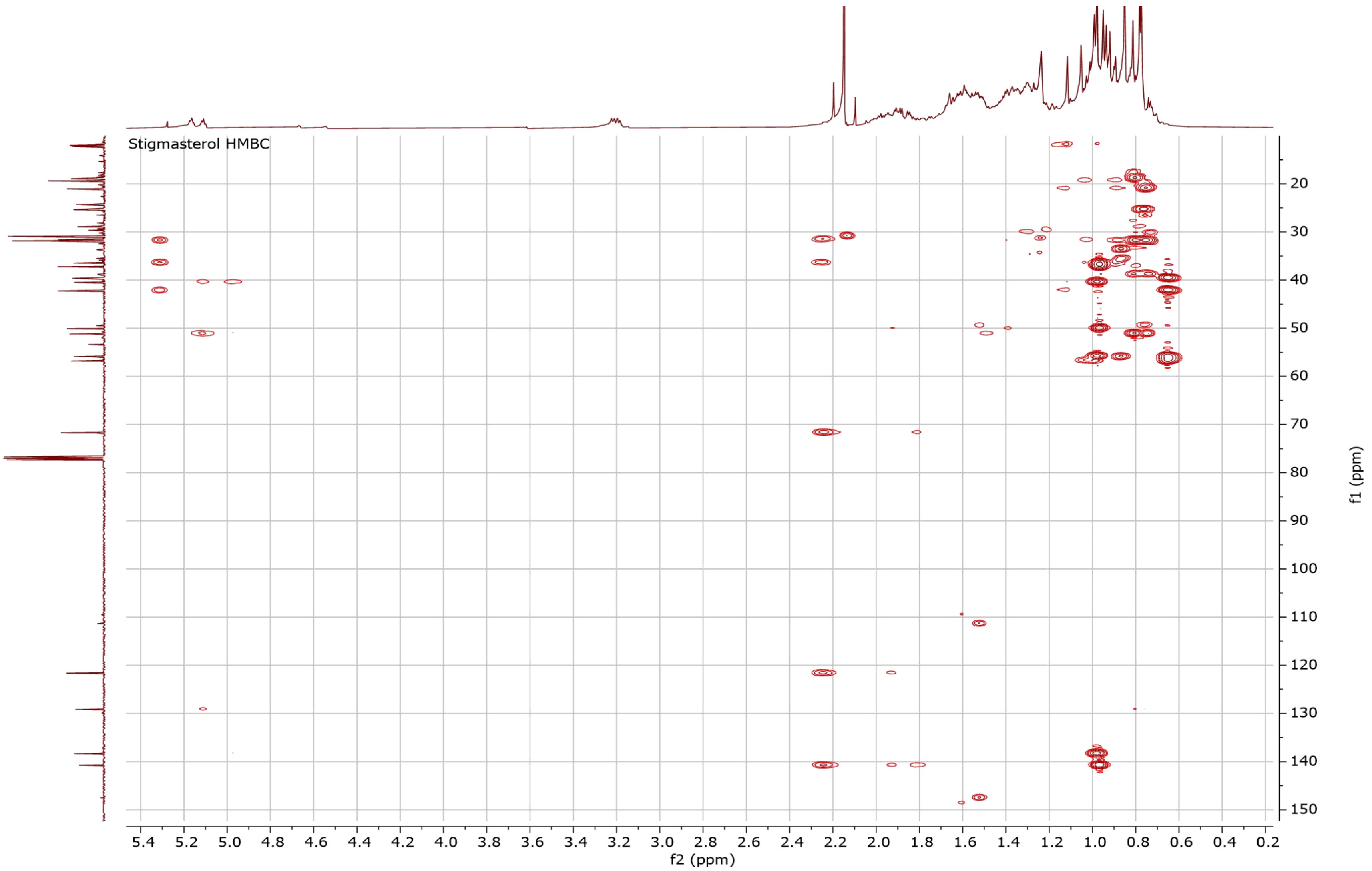

Figure S37: HMBC spectrum of compound **6**: stigmasta-5,22-dien-3β-ol (Stigmasterol)

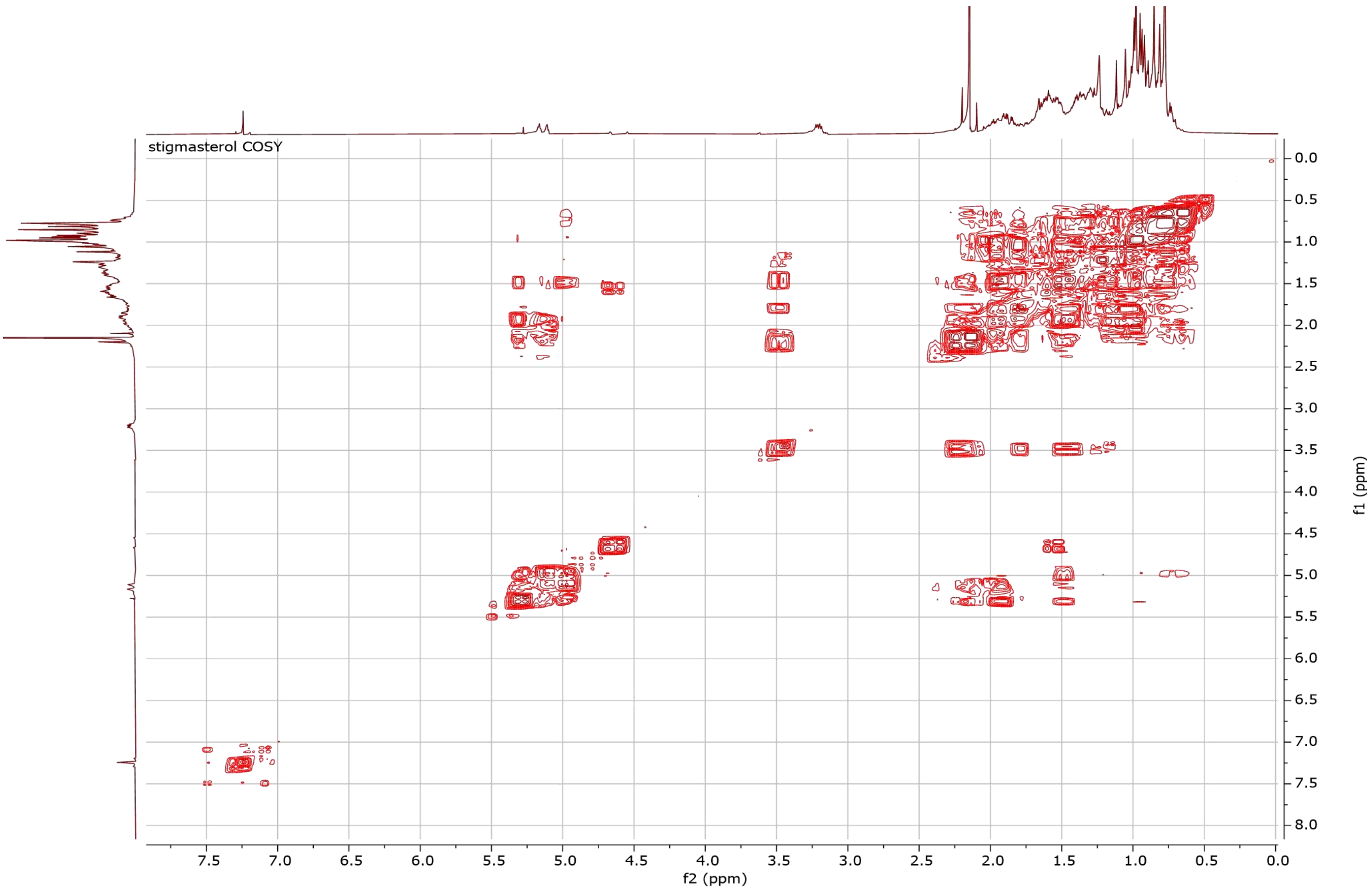

Figure S38: COSY spectrum of compound **6**: stigmasta-5,22-dien-3β-ol (Stigmasterol)

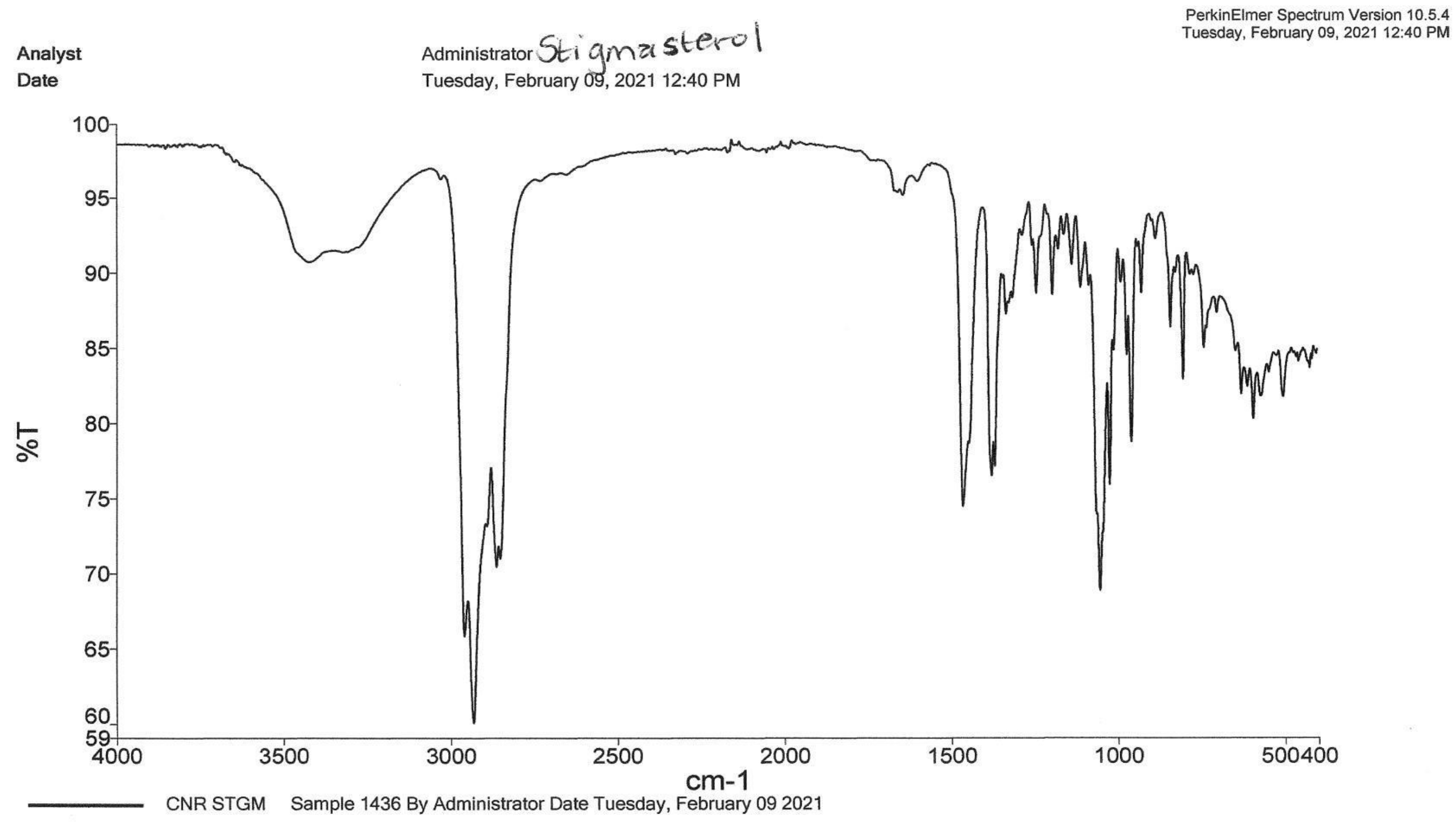

Figure S39: IR spectrum of compound **6**: stigmasta-5,22-dien-3β-ol (Stigmasterol)

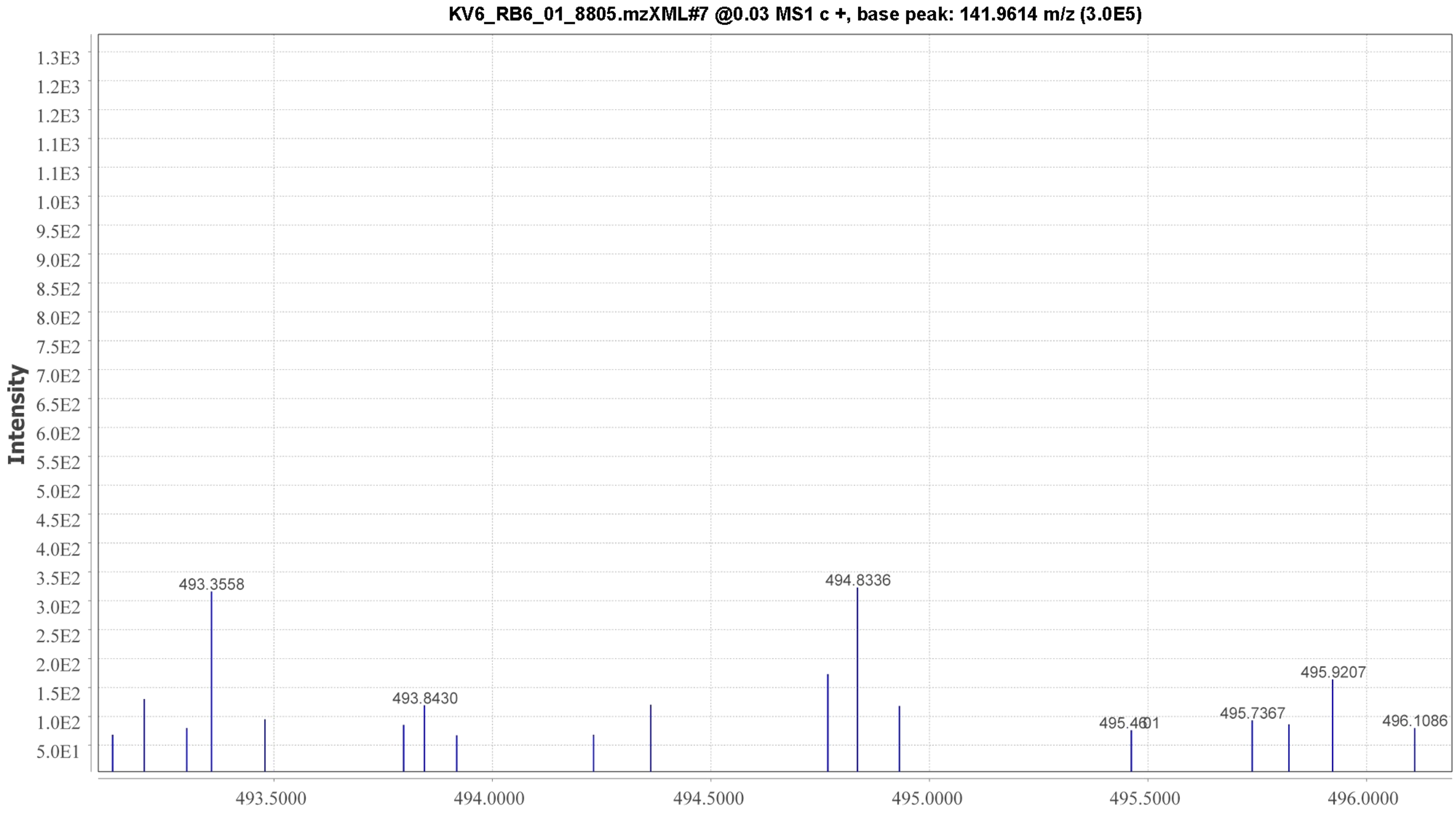

Figure S40: MS spectrum of compound **6**: stigmasta-5,22-dien-3β-ol (Stigmasterol)

Table S1: ^{1}H NMR and ^{13}C NMR spectral data of compounds **1** and **2**.

**^{1}H & ^{13}C number**	**^{1}H NMR (400 MHz, δ ppm)**		**^{13}C NMR (100 MHz, δ ppm)**	
	**Compound 1**	**Compound 2**	**Compound 1**	**Compound 2**
1α	1.37 (1H, m)	1.11 (1H, m)	38.9 (CH_2)	36.2 (CH_2)
1β	2.16 (1H, m)	2.75 (1H, m)	-	-
2α	1.62 (1H, m)	1.21 (2H, m)	19.2 (CH_2)	29.7 (CH_2)
2β	1.82 (1H, m)	-	-	-
3α	1.20 (1H, m)	1.19 (1H, m)	41.7 (CH_2)	41.3 (CH_2)
3β	1.47 (1H, m)	1.46 (1H, m)	-	-
4	-	-	33.3 (C)	33.4 (C)
5	1.32 (1H, m)	1.06 (1H, t, *J* = 21.2 Hz)	50.3 (CH)	51.7 (CH)
6α	1.70 (1H, m)	1.41 (1H, dd, *J* = 5.2, 4.4, 4.5 Hz)	19.3 (CH_2)	17.4 (CH_2)
6β	1.87 (1H, m)	1.87 (1H, q, *J* = 20.8 Hz)	-	-
7α	2.83 (2H, m) 2.37 (1H, dd, *J* = 0.8, 1.6 Hz)		29.7 (CH_2)	26.6 (CH_2)
7 β	2.71 (1H, dd, *J* = 1.2, 0.8 Hz)		-	-

8	-	-	127.2 (C)	146.5 (C)
9	-	-	148.6 (C)	146.0 (C)
10	-	-	37.5 (C)	38.4 (C)
11	6.61 (1H, s)	-	110.9 (CH)	183.4 (C)
12	-	-	150.7 (C)	150.5 (C)
13	-	-	131.4 (C)	123.7 (C)
14	6.81 (1H, s)	-	126.6 (CH)	187.5 (C)
15	3.08 (1H, m)	3.15 (1H, sept, *J*= 28.4 Hz)	26.8 (CH)	24.1 (CH)
16	1.23 (3H, d, *J* = 4.0 Hz)	1.24 (3H, d, *J* = 8.0 Hz)	22.5 (CH_3)	19.9 (CH_3)
17	1.22 (3H, d, *J* = 4.0 Hz)	1.22 (3H, d, *J* = 8.0 Hz)	22.7 (CH_3)	19.8 (CH_3)
18	0.92 (3H, s)	0.91 (3H, s)	33.3 (CH_3)	21.7 (CH_3)
19	0.90 (3H, s)	0.93 (3H, s)	21.6 (CH_3)	33.4 (CH_3)
20	1.16 (3H, s)	1.24 (3H, s)	24.7 (CH_3)	20.0 (CH_3)
12-OH	4.52 (OH, s)	7.20 (OH, s)	-	-

Table S2: ^{1}H NMR and ^{13}C NMR spectral data of compounds **3** and **6**.

**^{1}H & ^{13}C number**	**^{1}H NMR (400 MHz, δ ppm)**		**^{13}C NMR (100 MHz, δ ppm)**	
	**Compound 3**	**Compound 6**	**Compound 3**	**Compound 6**
1α	1.27 (1H, m)	1.04 (1H, dd, *J* = 7.6, 4.0 Hz)	38.2 (CH_2)	37.2 (CH_2)
1β	1.62 (1H, m)	-		
2α	1.60 (1H, m)	1.90 (1H, m)	23.6 (CH_2)	31.7 (CH_2)
2β	1.85 (1H, m)	1.94 (1H, m)		
3	4.49 (1H, t, *J* = 4.0 Hz)	3.22 (1H, m)	80.6 (CH)	71.7 (CH)
4	-	2.20 (1H, m)	37.7 (C)	42.3 (CH_2)
5	0.86 (1H, t, *J* = 10.8 Hz)	-	55.2 (CH)	140.7 (C)
6α	1.42 (1H, m)	5.32 (1H, d, *J* = 3.2, Hz)	18.2 (CH_2)	121.6 (CH)
6β	-	-		

7α	1.21 (1H, m)	<1.80> (2H, *J* = 4.0, 3.2 Hz)	32.6 (CH_2)	31.9 (CH_2)
7β	-			
8	-	2.15 (1H, m)	39.8 (C)	31.0 (CH)
9	1.54 (1H, t, *J* = 10.8 Hz	0.92 (1H, dt, *J*= 6.8, 5.8 Hz)	47.5 (CH)	50.1 (CH)
10	-	-	36.8 (C)	36.5 (C)
11α	1.86 (1H, m)	1.46 (2H, m)	23.5 (CH_2)	21.0 (CH_2)
11β	-			
12α	5.17 (1H, s)	1.17 (1H, m)	121.6 (CH)	39.7 (CH_2)
12β	-	1.98 (1H, m)		
13	-	-	145.2 (C)	42.2 (C)
14	-	1.01 (1H, m)	41.7 (C)	56.9 (CH)
15α	-	1.03 (1H, m)	26.9 (CH_2)	24.4 (CH_2)
15β	1 24 (2H, m)	1.51 (1H, m)		
16	1.12 (1H, m)	1.21 (1H, m)	26.1(CH_2)	24.4 (CH_2)
17	-	1.10 (1H, m)	32.5 (C)	55.9 (CH)
18	1.94 (1H, t, *J* = 1.3 Hz	0.68 (3H, s)	46.8 (CH_2)	12.0 (CH_3)

19α	1.00 (1H, m)	0.95 (3H, s)	47.2 (CH_2)	19.4 (CH_3)
19β	1.26 (1H, m)	-		
20	-	2.00 (1H, dd, *J* = 4.8, 8.8 Hz)	31.1 (CH_2)	40.5 (CH)
21α	2.26 (1H, m)	0.98 (3H, s)	34.7 (CH_2)	21.2 (CH_3)
21β	-	-		
22α	1.23 (1H, m)	5.05 (1H, d, *J* = 8.8 Hz)	37.1 (CH_2)	138.3 (CH)
22β	-	-		
23	0.85 (3H, s)	4.97 (1H, d, *J* = 4.0Hz)	37.1 (CH_2)	129.3 (CH)
24	0.85 (3H, s)	1.48 (1H, m)	16.7 (CH_3)	51.2 (CH)
25	0.82 (3H, s)	1.18 (1H, m)	19.7 (CH_3)	29.7 (CH)
26	0.96 (3H, s)	0.81 (3H, d, *J*= 6.0 Hz	15.5 (CH_3)	21.0 (CH_3)
27	1.22 (3H, s)	0.78 (3H, d, *J*= 6.0 Hz)	25.9 (CH_3)	21.0 (CH_3)

28α	0.82 (3H, s)	1.12 (1H, m)	28.0 (CH_3)	25.4 (CH_2)
28β	-	1.37 (1H, m)		
29	0.85 (3H, s)	0.74 (3H, t, *J*= 15.6 Hz)	33.3 (CH_3)	12.2 (CH_3)
30	0.81 (3H, s)	-	23.7 (CH_3)	-
1′	-	-	173.7 (COO)	-
2′	2.29 (1H, m)	-	34.8 (CH_2)	-
3′	1.92 (1H, m)	-	25.2 (CH_2)	-
4′-13′	1.18-1.27		29.1- 29.7 $(CH_2)_n$	-
14′	1.27 (2H, m)	-	31.9 (CH_2)	-
15′	1.27 (2H, m)	-	22.7 (CH_2)	-
16′	0.85 (3H, s)	-	14.1 (CH_3)	-

Table S3: ^{1}H NMR and ^{13}C NMR spectral data f compounds **4**, **5a** and **5b**.

**^{1}H & ^{13}C number**	**^{1}H NMR (400 MHz, δ ppm)**			**^{13}C NMR (100 MHz, δ ppm)**		
	**Compound 4**	**Compound 5a**	**Compound 5b**	**Compound 4**	**Compound 5a**	**Compound 5b**
1	-	-	-	132.5 (C)	132.5 (C)	138.1 (C)
2	-	-	-	106.0 (CH)	106.2 (CH)	105.5 (CH)
3	-	-	-	152.8 (C)	152.7 (C)	152.7 (C)
4	-	-	-	132.6 (C)	135.8 (C)	135.8 (C)
5	-	-	-	153.0 (C)	152.8(C)	152.8 (C)
6	-	-	-	105.5 (CH)	105.7 (CH)	105.7 (CH)
1a	6.39 (1H, d, *J* = 3.6 Hz)	6.30 (1H, d, *J*= 5.2 Hz)	2.70 (2H, m)	130.3 (CH)	127.9 (CH)	37.0 (CH2)
1′a	6.52 (1H, d, *J* = 3.6 Hz)	6.36 (1H, d) *J*= 5.6 Hz	2.71 (2H, d) *J*= 6.4 Hz	124.1 (CH)	125.3 (CH)	31.0 (CH_2)
1′	-	-	-	117.9 (C)	124.1 (C)	129.9 (C)
2′	-	-	-	141.6 (C)	143.5 (C)	143.5 (C)
3′	-	-	-	137.3 (C)	138.9 (C)	138.8 (C)

4′	-	-	-	146.3 (C)	147.8 (C)	146.7 (C)
5′	6.36 (1H, d, *J* = 8.8 Hz)	-	-	102.9 (CH)	108.6 (CH)	108.2 (CH)
6′	6.75 (1H, d, *J* = 2.8 Hz)	-	-	120.3 (CH)	119.5 (CH)	119.5 (CH)
3-OCH_3	3.66 (3H, s)	3.71 (6H, s)	3.73 (6H, s)	55.9	55.8	55.8
4-OCH_3	3.70 (3H, s)	3.69 (3H, s)	3.70 (3H, s)	60.9	60.4	60.8
5-OCH_3	3.66 (3H, s)	3.71 (6H, s)	3.73 (6H, s)	55.9	55.8	55.8
4′-OCH_3	3.70 (3H, s)	3.56 (3H, s)	3.56 (3H, s)	56.2	56.0	56.0
1′′	-	4.44 (1H, d, *J*= 6.8 Hz)	4.67 (1H, d, *J*= 0.8 Hz)	-	105.0 (CO)	105.2 (CO)
2′′	-	-	-	-	73.8	73.8
3′′	-	-	-	-	76.2	76.1
4′′	-	-	-	-	69.2	69.2
5′′	-	-	-	-	76.7	76.7
6′′	-	-	-	-	61.0	61.0
2 x OH	5.43 (2H, bs)	5.49 (OH, bs)	5.49 (OH, bs)	-	-	-
